# The repeated evolution of stripe patterns is correlated with body morphology in the adaptive radiations of East African cichlid fishes

**DOI:** 10.1002/ece3.8568

**Published:** 2022-02-07

**Authors:** Sabine Urban, Jan Gerwin, C. Darrin Hulsey, Axel Meyer, Claudius F. Kratochwil

**Affiliations:** ^1^ 26567 Chair in Zoology and Evolutionary Biology Department of Biology University of Konstanz Konstanz Germany; ^2^ Present address: School of Biology and Environmental Science University College Dublin Belfield Ireland; ^3^ Present address: Institute of Biotechnology, HiLIFE University of Helsinki Helsinki Finland

**Keywords:** body morphology, convergence, CRISPR‐Cas9 cichlid, motion dazzle, pigmentation, trait correlation

## Abstract

Color patterns are often linked to the behavioral and morphological characteristics of an animal, contributing to the effectiveness of such patterns as antipredatory strategies. Species‐rich adaptive radiations, such as the freshwater fish family Cichlidae, provide an exciting opportunity to study trait correlations at a macroevolutionary scale. Cichlids are also well known for their diversity and repeated evolution of color patterns and body morphology. To study the evolutionary dynamics between color patterns and body morphology, we used an extensive dataset of 461 species. A phylogenetic supertree of these species shows that stripe patterns evolved ~70 times independently and were lost again ~30 times. Moreover, stripe patterns show strong signs of correlated evolution with body elongation, suggesting that the stripes’ effectiveness as antipredatory strategy might differ depending on the body shape. Using pedigree‐based analyses, we show that stripes and body elongation segregate independently, indicating that the two traits are not genetically linked. Their correlation in nature is therefore likely maintained by correlational selection. Lastly, by performing a mate preference assay using a striped CRISPR‐Cas9 mutant of a nonstriped species, we show that females do not differentiate between striped CRISPR mutant males and nonstriped wild‐type males, suggesting that these patterns might be less important for species recognition and mate choice. In summary, our study suggests that the massive rates of repeated evolution of stripe patterns are shaped by correlational selection with body elongation, but not by sexual selection.

## Graphical abstract

Across the ~1200 cichlid species of the East African rift lakes, melanic horizontal stripes have evolved numerous times. By applying comparative analyses, hybrid crosses, and a behavioral experiment using a CRISPR/Cas9 knockout mutant, our study aims at understanding the ecological function of these horizontal stripe patterns. We suggest that the massive rates of repeated evolution of stripe patterns are shaped by correlational selection with body elongation, but not by sexual selection.

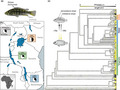

## 1 INTRODUCTION

Across the more than 1200 cichlid species of the East African rift lakes, melanic horizontal stripes have evolved numerous times and are a prime example of convergent evolution (Kratochwil et al., 2018). Different functions of stripes have been proposed including camouflage (Cott, 1940; Longley, 1916; Stevens & Merilaita, 2009; Thayer, 1918) and social signaling (Barlow, 2008; Seehausen et al., 1999). The repeated evolution of stripes suggests that they evolved by natural selection and constitute adaptations to similar ecological niches (Harvey & Pagel, 1991) with shared selection pressures (Arendt & Reznick, 2008; Losos, 2011; McGhee, 2011; Ord & Summers, 2015). Alternatively, it has been hypothesized that the evolution of coloration in cichlids is driven by sexual selection (i.e., nuptial coloration) (Allender et al., 2003; Couldridge & Alexander, 2002; Knight & Turner, 1999; Maan et al., 2004). Yet, a comparative study of East African cichlid fishes has shown that strength of sexual selection has no detectable effect on stripe pattern evolution (Seehausen et al., 1999), and to the best of our knowledge, an influence of stripe patterns for mate choice or preference has never been formally tested. From an ecological viewpoint, the evolution of stripes has been mainly linked to shoaling and piscivorous feeding, suggesting that these trait combinations might be effective strategies to decrease predation risk or to avoid being seen by prey (Seehausen et al., 1999).

Cryptic color patterns (e.g., spots, blotches, vertical bars) allow individuals to blend in with the background and reduce the probability of detection by predators, but they may no longer be advantageous when the prey moves (Cott, 1940; Thayer, 1918) because motion‐sensitive visual circuits of a stationary predator can easily detect movement against a stationary background. Conversely, horizontal stripes that might be considered being conspicuous in many environments may become beneficial when the individual moves (Allen et al., 2013; Brodie, 1992; Jackson et al., 1976). This special type of camouflage is called motion dazzle as it prevents successful capture during motion by causing predators to misjudge the direction or speed of prey movement. Several studies showed that such motion dazzle patterns might be involved in hampering a predator's ability to intercept a moving prey (Allen et al., 2013; Brodie, 1992; von Helversen et al., 2013; Jackson et al., 1976; Kelley & Kelley, 2014; Rojas et al., 2014). It is generally accepted that motion dazzle patterns are advantageous for mobile species that are highly detectable against the stationary background, while cryptic pigmentation patterns are advantageous for less‐mobile species that rely on camouflage to reduce detection (Halperin et al., 2016). A beneficial trait correlation of stripe patterns with body length was first shown for several reptile species (Allen et al., 2013; Murali & Kodandaramaiah, 2017). This trait correlation redirects predator attacks to the tail and thereby reduces the probability of individuals being captured (von Helversen et al., 2013) suggesting that the effectiveness of dazzle patterns depends on body shape (Murali & Kodandaramaiah, 2017).

In fish, body elongation is a major axis of body shape divergence (Astudillo‐Clavijo et al., 2015; Clabaut et al., 2007; Claverie & Wainwright, 2014; Kautt et al., 2020; Muschick et al., 2012) and has implications for a fish's susceptibility to predators (Chivers et al., 2008; Price et al., 2015), swimming performance (Rouleau et al., 2010), and attractiveness to mates (Head et al., 2013). Thereby, a combination of stripes and an elongated body shape could represent an adaptation to predator–prey interactions, for example, high swimming performance while impairing a predators’ perception of its prey through motion dazzle.

Understanding how correlations of traits (e.g., body shape and color patterns) evolve, neutrally or under different types of selection, and identifying the underlying genetic correlations can contribute to the understanding of the evolutionary effects of trait correlations (Dingemanse et al., 2012; Lande & Arnold, 1983; Sih et al., 2004). Current models of rapid divergence emphasize the importance of linkage and pleiotropy in adaptation and speciation (Noor & Bennett, 2009; Seehausen et al., 2014; Via, 2012). Hence, horizontal stripes could have evolved together with other phenotypic traits such as body shape due to a number of adaptive or nonadaptive reasons like genetic constraints such as linkage, in which two or more loci each affecting different traits are genetically linked and therefore tend to be inherited together. Pleiotropy, in which a single locus causally affects two or more traits (McKinnon & Pierotti, 2010; Saltz et al., 2017), could lead to a similar outcome. In general, trait correlations caused by pleiotropy are not expected to break down simply through neutral processes (Jones et al., 2003). On the other hand, trait correlations caused by genetic linkage and built up by correlational selection are normally expected to erode eventually through recombination, unless (correlational) selection is strong and persistent. This causes trait correlations that are generated by genetic linkage to be transient, and it was argued that they contribute little to evolutionary change (Saltz et al., 2017).

Cichlids are ideal for the investigation of trait correlations both from an evolutionary (looking across whole phylogenies) and a genetic viewpoint, since many species can be hybridized and most often even produce fertile offspring. Such hybrid crosses for example permitted the identification of the genetic basis of horizontal stripe patterns in East African cichlids. A single gene, *agouti*‐*related peptide 2* (*agrp2)*, was identified to facilitate the repeated evolution of stripes (Henning et al., 2014; Kratochwil et al., 2018). High expression of the “stripe inhibitor” gene *agrp2* prevents stripe pattern formation, and low expression permits their appearance (Kratochwil et al., 2018, 2019). Agouti family genes have been shown to control both color pattern divergence (Henning et al., 2014; Kratochwil et al., 2018) and growth, obesity, and energy metabolisms (Duhl et al., 1994; Song & Cone, 2007). Therefore, this locus is of particular interest to investigate trait correlations, since *agrp2* might act pleiotropically.

Knowledge of the genetic basis of stripes, a potentially pleiotropic function of the underlying gene(s), and the availability of large comparative datasets allow us now to take a further step toward understanding the ecological function of horizontal stripe patterns. Moreover, technological advances in genome editing methods introduce the potential to solely examine the effect of coloration traits in a behavioral experiment. By applying novel CRISPR/Cas9 tools, we can effectively remove stripe patterns and thus examine the role of sexual selection in response to the altered color pattern.

Here, we investigate (1) whether there is a correlation between body elongation and stripe patterns using comparative analyses, (2) whether the two traits are genetically constrained by linkage or pleiotropy for which we analyze two sets of hybrid crosses, and (3) whether there are signatures of selection on these traits. (4) Moreover, we explore whether stripes potentially play a role in sexual selection by using a classic two‐choice behavioral experiment. To do so, we let females of a nonstriped species choose between a nonstriped wild‐type male and a striped *agrp2* knockout mutant of the same species which was generated using the CRISPR/Cas9 genome editing technique.

## 2 METHODS

### 2.1 Data collection

We compiled a large photographic dataset of 461 species from all major East African cichlid lineages. Images were compiled from various online image databases and textbooks (listed in Table S2). In striped species, stripe patterns are usually present in both sexes but they are sometimes covered by the male's nuptial coloration. We therefore scored a species as striped when either the male or the female possessed a stripe pattern and measured the elongation index of the striped individual. However, only a small percentage of species shows stripes in only one sex and, if this is the case, it mostly affects how clear the pattern is.

For many species, there is only a single photograph available. All images were required to be in lateral view so that standard length and body elongation could be measured reliably. We further analyzed individuals for the presence of stripe pattern in three different ways (Figure S2): binary (0/no stripes, 1/stripes), categorical (0/no stripes, 1/spotted, 2/partially striped, 3/fully striped, 4/oblique stripe; only used for Table S1), and continuous (percentage of the midlateral stripe region covered by melanin). To calculate the elongation index, we measured the standard length, from the tip of the snout to the base of the caudal fin, of each fish as well as body depth, maximum distance between the most anterior part of the pelvic fin and dorsal fin. Since most photographs did not have a scale bar, the measures of standard length and body depth do not account for absolute size. The elongation index was then calculated as the standard length divided by body depth. For all measurements, we used the software Fiji (Schindelin et al., 2012). The raw data are available in the electronic (Table S1).

To examine the evolution of stripes in cichlids, we built a supertree combining trees from several different studies (Dunz & Schliewen, 2013; Hulsey et al., 2017, 2018, 2019; Hulsey, Zheng, et al., 2018; Irisarri et al., 2018; Kratochwil et al., 2018; Malinsky et al., 2018; McGee et al., 2016; Meier et al., 2017). Because it facilitated including the most species of cichlids into a single tree, we first generated a phylogeny of cichlid species using the mitochondrial *nd2* gene (NCBI accession numbers listed in Table S3) as this is the most comprehensive dataset available at this time. For this, we used the evolutionary model GTR + Gamma + Inv. The codon positions were examined as separate partitions. For tree building, we used BEAST v1.10.1 (Suchard et al., 2018) that generates trees with branch lengths in units of time. The recovered set of *nd2* based phylogenetic hypotheses was highly similar to previous results for this gene (Salzburger et al., 2005; Wagner et al., 2012).

After examining the results with the *nd2* gene tree alone, we combined this tree with ten other published phylogenies of African cichlids that incorporated information from the nuclear genome (Dunz & Schliewen, 2013; Hulsey et al., 2019; Hulsey, Holzman, et al., 2018; Hulsey, Zheng, et al., 2017, 2018; Irisarri et al., 2018; Kratochwil et al., 2018; Malinsky et al., 2018; McGee et al., 2016; Meier et al., 2017). Consensus phylogenies from these studies were combined using the “mrp.supertree” function with the “pratchet” method as implemented in the R program “phytools” (Revell, 2012) which estimates the MRP (matrix representation parsimony) supertree from a set of input trees. We used the “optim.parsimony” method as implemented in the R package “phangorn” (Schliep, 2011). The function “optim.parsimony” tries to find the maximum parsimony tree nearest neighbor interchange rearrangements as well as subtree pruning and regrafting.

Our final dataset, which covered phylogenetic as well as phenotypic information, included 461 species of East African cichlids (Data S1). To infer transitions from having no stripe to gaining stripes, q_01_, as well as from having stripes to losing them, q_10_, we imported a set of trees into the program Mesquite and then used the “Summarize State Changes Over Trees” function.

### 2.2 Comparative analyses

To determine whether there was an association between the presence of horizontal stripes and body elongation, we performed phylogenetic ANOVAs across the African cichlid supertree using “phytools” (Revell, 2012). We performed phylogenetic ANOVAs running simulations (nsim = 1000) with three stripe phenotypes as grouping factors: 1. presence of both midlateral and dorsolateral stripes versus species lacking either of these stripes. 2. Dorsolateral stripe versus species with no stripe. 3. Midlateral stripe versus species with no stripe.

To determine whether there was macroevolutionary evidence of stabilizing selection on body elongation in association with the presence or absence of stripes, we also fit a series of Brownian motion (BM) and Ornstein–Uhlenbeck (OU) models to body elongation evolution using the package “OUwie” (Beaulieu et al., 2012).

Prior to the evolutionary model fitting, we generated stochastic character maps (simmaps) using the function “make.simmap” implemented in the R package “phytools” (Revell, 2012). For each input tree simulated, we conducted a single simmap simulation using the “sym” transition model that treats the transition between striped and nonstriped as equal. We chose the “sym” function because the range of inferred transitions between striped and nonstriped overlapped, and we did not want to bias the results.

The support for more parameter‐rich models over models with fewer parameters was assessed using AIC scores. We asked first whether there was more support for a model containing a single optimum value, *α*, as compared to a simple model of BM for body elongation evolution in the cichlids examined. This first OU model did not incorporate any differences between striped and nonstriped species. So, we next asked whether there was support for an OU process with different optima associated with the presence and absence of stripes. Support for this two‐optima model over a model with a single optimum would further support the hypothesis that stripes are evolutionarily associated with a difference in body elongation. Finally, we asked whether there was support for different selective pulls toward the morphological optimum for nonstriped, *α*_1_, and striped, *α*_2_, cichlid lineages.

### 2.3 Experimental crosses

All data on obtaining experimental crosses and quantification of stripes are described in Kratochwil et al. (2018). Here, we phenotyped F_2_ individuals from a Lake Malawi cross between a striped species (*Pseudotropheus cyaneorhabdos)* and a nonstriped species (*Chindongo demasoni*; previously: *Pseudotropheus demasoni*) and a second cross of Lake Victoria cichlids again consisting of a striped species (*Haplochromis sauvagei)* and a nonstriped species (*Pundamilia nyererei*). For all fishes, we estimated the elongation index and three scores to quantify the number and/or continuity of stripes (binary, categorical, continuous). We checked for normal distribution of data using the Shapiro–Wilk test of normality and decided to use a nonparametric test to calculate trait correlation. We used Kendall's tau‐b correlation coefficient to test whether body elongation and stripes (continuous measurement) are correlated. Additionally, we compared the striped and nonstriped individuals (based on binary scoring) using a Wilcoxon test.

### 2.4 Mate preference experiments

To test for a role of stripe patterns in species recognition and sexual selection, we tested ten *P*. *nyererei* females for a preference in a classical two‐choice experimental setup. Females were allowed to choose between two males of the same species: a nonstriped wild‐type male and a striped sibling CRISPR‐Cas9 *agrp2* mutant (Kratochwil et al., 2018) of the same size. The mutant stimulus male differed genetically only from its sibling stimulus male in that the *agrp2* had a loss of function mutation induced by CRISPR‐Cas9. The two male individuals (the same mutant and the same wild‐type male were used with all females) used for the experiment have successfully sired offspring before entering the experiment. The experimental setup consisted of three separate tanks placed next to each other to reduce the test to visual cues only because the mutation in *agrp2* rendered the stripe pattern phenotype of the mutant male. It was shown that species‐isolating female choice is likely based primarily on such visual information (Jordan et al., 2003).

A single female was placed in the middle, while the left and right tanks each contained one stimulus male. The central tank was delineated into three areas (the left choice area in front of the left male, the neutral area in the center, and the right choice area in front of the right male). The aquarium was lit by one daylight neon lamp situated next to the camera, while the room was maintained in the dark to avoid reflections. The room temperature was constantly kept at 25°C under a 12:12‐h light:dark cycle.

All fish were acclimatized overnight (minimum 12 h) and in groups with other fish to reduce stress levels. After acclimatization, all other fish were carefully removed from the setup, and we allowed another acclimatization of 20 min. We then recorded females’ positions continuously with a digital video camera positioned above the setup for 40 min. Then, we switched the two stimuli males to correct for female side preference and filmed the female again for 40 min. For tracking, we used the R package “trackR” (https://github.com/swarm‐lab/trackR). From this, we calculated a preference index as the amount of time spent in front of male A divided by the total amount of time spent in the two choice areas. A female was assumed to prefer *male A* over *male B* when the preference index (amount of time spent in front of *male A* divided by the total amount of time spent in the two choice areas) was above 50%. All calculations were performed using R 3.0 software (R Development Core Team, 2019).

## 3 RESULTS

### 3.1 Horizontal stripes evolved repeatedly across East African radiations

**FIGURE 1:**
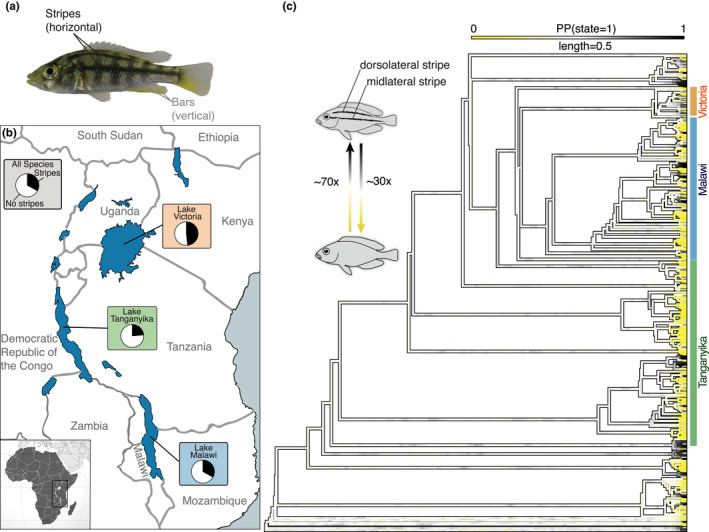
Repeated evolution of horizontal stripes across East African cichlid radiations. (a) Picture of a female *Haplochromis chilotes* showing both stripe (black) and bar patterns (grey). (b) Map of East Africa highlighting Lakes Victoria, Tanganyika, and Malawi that encompass the three main radiations. Pie charts give the percentage of striped species (overall ~30%, in gray). The percentage of striped species was inferred from the presented data (461 species). Color codes show the major radiations of Lakes Tanganyika (green), Malawi (blue), and Victoria (orange). (c) Phylogenetic supertree of 461 East African cichlids with likelihood reconstructions of ancestral states (yellow–white–black gradient with yellow indicating a nonstriped state and black a striped state) of the stripe phenotype that evolved ~70 times independently. White branches moving away from the tips of the tree represent the inherent uncertainty in our ancestral state reconstructions

Of the 461 species in our dataset, 33% (152 species) show either a midlateral or a dorsolateral stripe (Figure 1a,c). In total, 32% (149 species) show only a midlateral stripe and 26% (119 species) show a dorsolateral stripe (Figure 1b). There are some species that only have a midlateral but no dorsolateral stripe (*N* = 32), but only three species show exclusively a dorsolateral but no midlateral stripe. Ancestral state reconstruction of the presence of horizontal stripes (binary scoring) revealed that stripes evolved on average 73 (range: 64–82) times independently and were lost on average 28 (range: 19–37) times (Figure 1c). Based on these, many frequent gains and losses the ancestral state cannot be reliably determined (PP = 0.5).

### 3.2 Stabilizing selection on body elongation is acting stronger in striped species

**FIGURE 2:**
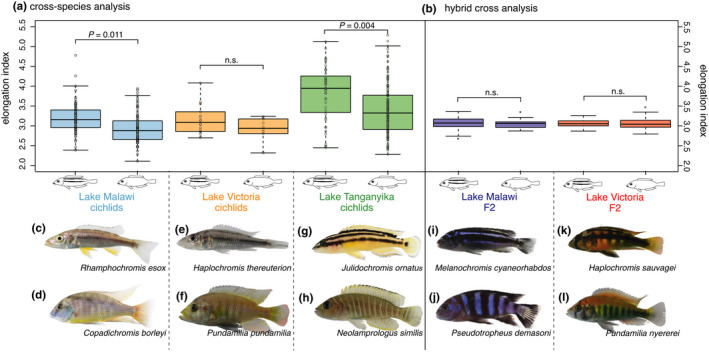
Striped fishes show an elongated body shape across cichlid radiations of the East African Great Lakes. (a) While both Lake Malawi (blue) and Lake Tanganyika (green) cichlids show a substantially elongated body shape if they have any horizontal stripes (phylogenetic ANOVA), this pattern is not supported in Lake Victoria cichlids (orange; phylogenetic ANOVA; *p* = .199). (b) There is no evidence for genetic linkage of horizontal stripes and body elongation since the elongation index, measured in *F*_2_ offspring from two hybrid crosses, is not significantly correlated with a stripe phenotype. Importantly, the two parental species of the Lake Malawi (*i* and *j*) and Lake Victoria (*k* and *l*) hybrid crosses show different elongation indices which is given below the species' names. Photographs show striped species endemic to Lake Malawi (c and d), Lake Victoria (e and f), and Lake Tanganyika (g and h)

To determine whether there is macroevolutionary evidence of stabilizing selection on body elongation in association with horizontal stripes, we fit a series of Brownian motion (BM) and Ornstein–Uhlenbeck (OU) models to the evolution of body elongation (Beaulieu et al., 2012). These comparative analyses revealed that stabilizing selection is supported for body elongation in association with stripe patterns. The OUMA model (Table 1) is the most supported model. While this model supports a different mean (optima) between striped and nonstriped species, it supports greater stabilizing selection (*α*) of body elongation in striped as compared to nonstriped species. This suggests that stabilizing selection on body elongation is acting more strongly on striped species than on nonstriped species and striped species have a higher optimum elongation index (Table 1). Also, there is support for a different body elongation optimum, or mean value, for the striped species versus the nonstriped species which is consistent with the phylogenetic ANOVA results (Figure 2).

**TABLE 1:** Results of different models of trait evolution

	AIC	Optima		*σ*		*α*	
		No stripe	Stripe	No stripe	Stripe	No stripe	Stripe
BM1	1149.77	3.28	3.28	44.04	44.04	NA	NA
OU1	897.99	3.19	3.19	83.40	83.40	87.09	87.09
OUM	895.11	3.06	3.35	85.07	85.07	91.11	91.11
OUMA	876.98	3.06	3.31	84.66	84.66	91.90	92.24

Note

Shown are different Akaike's information criteria (AIC) values for different Brownian motion (BM) and Ornstein–Uhlenbeck (OU) models to test for macroevolutionary evidence of stabilizing selection on body elongation in association with horizontal stripes. Different elongation optima for striped and nonstriped species are reported for the different models; *σ* gives the rate of divergence and *α* is a parameter of stabilizing selection.

### 3.3 Horizontal stripes are largely associated with body elongation

To test whether horizontal stripes are associated with body shape, that is, elongation, across East African radiations, we calculated the elongation index as the standard length divided by body depth and employed a phylogenetic ANOVA. The results show that both the dorsolateral and midlateral stripe individually are significantly associated with body elongation (phylogenetic ANOVA; *p* = .018 in the case of just the dorsolateral stripe and phylogenetic ANOVA; *p* = .012 with the midlateral stripe, Table 1 and Figure 2). Showing any stripe pattern (either dorsolateral or a midlateral stripe) is significantly associated with body elongation as well (phylogenetic ANOVA; *p* = .009). While both cichlids from the older Lake Malawi and Lake Tanganyika cichlid radiations show an association between a substantially elongated body shape and horizontal stripes (phylogenetic ANOVA; *p* = .011 in Lake Malawi cichlids and phylogenetic ANOVA; *p* = .004 in Lake Tanganyika cichlids), this association is not statistically supported in Lake Victoria cichlids (phylogenetic ANOVA; *p* = .199).

To investigate whether stripes and body elongation are genetically linked traits and therefore inherited together, we analyzed two independent hybrid crosses from Lake Victoria and Lake Malawi for a correlation between body elongation and stripes. One might have expected to find the same correlation of stripes with body elongation in F_2_ offspring of those crosses (see Section 2.3). However, there was no correlation between body elongation and presence of stripes in the crosses from Lake Malawi (Kendall rank correlation; *r* = −.0024, *p* = .96) and Lake Victoria (Kendall rank correlation; *r* = .022, *p* = .73). A comparison of elongation indices between the striped and nonstriped F_2_ offspring showed that elongation does not differ significantly between individuals of the Lake Malawi cross (Wilcoxon test; *p* = .5406) as well as the Lake Victoria cross (Wilcoxon test; *p* = .7604). Hence, we conclude that, these two traits are inherited independently and are neither linked genetically (linked genes in the same genetic region controlling both traits) nor linked through pleiotropy (same gene controlling both traits).

### 3.4 No evidence for a role of sexual selection acting on stripes

To test for a role of horizontal stripes in species recognition and/or mate preference, we performed a two‐choice experiment with females of the nonstriped species *P*. *nyererei* measuring the time the female spends in the vicinity of the wild‐type or the CRISPR‐Cas9 phenotypically choice male. Females could choose between two stimulus males of the same size: a striped CRISPR‐Cas9 knockout (Kratochwil et al., 2018) and a wild‐type sibling showing no stripes from the same species. From this, we calculated a preference index as the amount of time spent in front of *male A* divided by the total amount of time spent in the two choice areas (see Section 2). The mean preference index across trials (*N* = 10) is 0.46 which is suggesting that *P*. *nyererei* females do not visually differentiate between the nonstriped wild‐type *P*. *nyererei* and the striped mutant *P*. *nyererei* (or at least do not prefer one over the other) since they spent approximately the same time with both stimulus males.

## 4 DISCUSSION

Here, we reconstructed the number of times horizontal stripes evolved as well as their phenotypic associations in a robust phylogenetic framework by generating a supertree encompassing 461 East African cichlid species. This supertree consists of several published phylogenies based on mitochondrial and nuclear data. Our results enhance our understanding of the association of stripes with the evolution of body shape and mate choice in African cichlids.

Comparative analyses showed that stripes are present in one third of East African cichlid species and evolved many times independently with stripes having been gained ~70 times and having been lost again ~30 times. While the exact number of transitions might be influenced by gene flow and lack of treelike structure within the Lake Malawi and Victoria radiations, we can conclude that stripes evolved many times independently and more frequently than one might have expected.

Moreover, stripes are not confined to specific clades in the molecular phylogeny (Figure 1c) reflecting the high degree of evolutionary lability of stripes. Based on our phylogeny, we were not able to estimate the ancestral state of African cichlids, but based on previous work suggests that the frequent loss of stripes is explained by the “stripe inhibitor” gene *agrp2* (Kratochwil et al., 2018), it seems more parsimonious that stripe presence is the ancestral state of East African cichlids (although it was likely not as exaggerated as in extant striped cichlids from the Great Lakes) and that the independent loss of stripes was caused by independent mutations affecting *agrp2* expression (Kratochwil, 2019; Kratochwil et al., 2018; Urban et al., 2021) and possibly other genes (Gerwin et al., 2021).

Exploring the selective forces which have led to the repeated gains and losses of stripe patterns, comparative analyses revealed that the presence of stripe patterns is significantly correlated with body elongation in most East African cichlids (Figure 2). While the mean elongation index is slightly higher in striped Lake Victoria cichlids (3.134) than in nonstriped species (3.047), an association between stripes and an elongated body shape is not supported statistically. One possible explanation is the relatively small sample size of Lake Victoria cichlids (*N* = 29) compared to Lake Malawi (*N* = 137) and Lake Tanganyika cichlids (*N* = 174) in which this association was significant. Also, as evident from Figure 2, the Lake Tanganyika radiation, the oldest lake, shows the most variation, followed by the Lake Malawi radiation and the youngest radiation of Lake Victoria. Further analyses revealed that the trait correlation between stripe pattern and body elongation is subject to stabilizing selection that is acting more strongly on striped species than on nonstriped species. Notably, different elongation optima are supported for striped species than for nonstriped species (Table 1). In reptiles, a combination of body length and stripes reduces the probability with which moving prey is captured by affecting the predators’ perception of speed (von Helversen et al., 2013). This motion dazzle effect of stripes is a form of defensive color pattern suggested to prevent successful capture during motion by causing predators to misjudge the direction or speed of prey movement. It has been hypothesized to play a role in motion camouflage in a variety of animals such as snakes (Brodie, 1989, 1992, 1993; Creer, 2005; Jackson et al., 1976) and lizards (Murali et al., 2018), and many studies have demonstrated its effect in humans (Hogan et al., 2016, 2017; Stevens et al., 2011). While in fish, body elongation has key implications for fitness, such as susceptibility to predators (Chivers et al., 2008; Price et al., 2015) and swimming performance (Rouleau et al., 2010), stripe patterns in cichlids were previously associated with shoaling behavior as well as a piscivorous feeding mode (Seehausen et al., 1999). The evolution of vertical bar patterns, on the other hand, was associated with structurally complex habitats, such as rocky substrates and vegetation. Vertically barred snakes were shown to be more secretive and rely on crypsis or aggression as their primary mechanism of defense (Jackson et al., 1976). While vertical bars and horizontal stripes can be easily obtained by hybridization (Gerwin et al., 2021) in nature, they rarely occur in the same species, which indicates that vertical bars and horizontal stripes might have different ecological roles.

We propose that the association of body elongation and stripes in cichlids could similarly reduce the probability with which individuals are captured by predators (von Helversen et al., 2013) by creating a motion dazzle pattern (Murali & Kodandaramaiah, 2017) as it has been shown for reptiles (Allen et al., 2013; Murali & Kodandaramaiah, 2017). However, such a motion dazzle pattern could also be beneficial for predatory species where it could cause the prey to misjudge the predator's speed which is suggested by the association of stripes with a piscivorous feeding mode. Such a type of morphological integration has been suggested to play a role in the rapid evolution of a number of traits in cichlids and other adaptively diverging groups (Albertson et al., 2005; Hulsey, Machado‐Schiaffino, et al., 2017; Husemann et al., 2017) suggesting an important role of either pleiotropy or genetic linkage (Saltz et al., 2017). However, body elongation and stripes segregate in F_2_ offspring of the two hybrid crosses (Figure 2b). While in each hybrid crosses, the major effect “stripe suppressor gene” *argp2* separates striped cichlids from nonstriped cichlids (Henning et al., 2014; Kratochwil et al., 2018), a correlation with body elongation was not supported. This result implies that body elongation and horizontal stripes do not have a shared genetic basis or shared major effect loci and are thus not correlated due to genetic linkage or pleiotropy of *agrp2*. The correlation of the traits might therefore rather be caused by selection pressures on both body shape and color patterns. Hence, the trait correlation of body elongation and stripes is not morphologically integrated, nor do they represent genetic modularity, but evolve due to strong correlational selection which favors combinations of traits that work in concert (Brodie et al., 1995).

**FIGURE 3:**
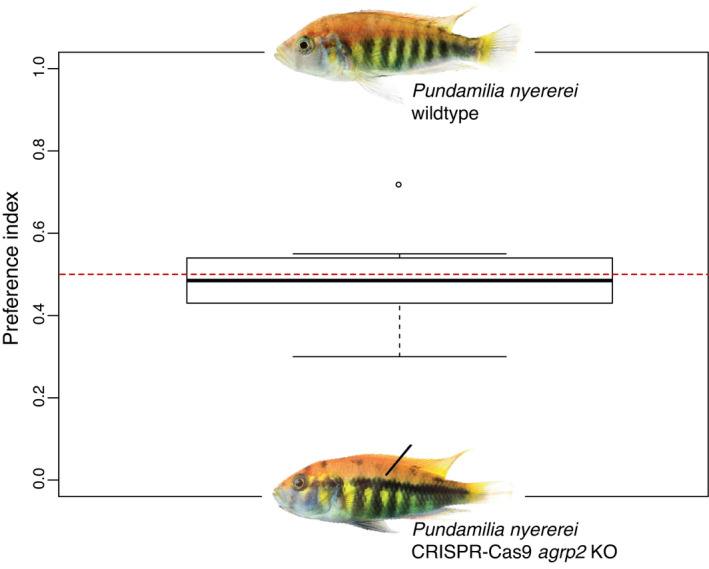
Horizontal stripes may not be relevant for species recognition and mate preference. The preference index was calculated as the amount of time spent in front of *male A* divided by the total amount of time spent in the two choice areas. The dashed red line indicates 0.5 which means no deviation from random choice. A value >0.5 would indicate a preference for the wild‐type *Pundamilia nyererei*, while a value <0.5 indicates a preference for the striped *agrp2* knockout

In cichlids, color patterns were shown to be important in species recognition (Couldridge & Alexander, 2002; Seehausen & van Alphen, 1998; Seehausen et al., 1998) and it was proposed that disruptive sexual selection is one of the main contributors to the rapid rate of speciation seen in cichlids (Maan et al., 2004). Such premating barriers are considered essential for reproductive isolation in closely related species, such as cichlids (Butlin, 1987; Coyne & Orr, 2004; Rometsch et al., 2020). However, several recent studies have shown that species divergence of reef fishes is frequently first characterized by the evolution of different color patterns, especially among breeding males (Allen et al., 2015; Hench et al., 2019; Rocha & Bowen, 2008; Victor & Randall, 2010, 2014). Yet, when testing for a role of sexual selection (Figure 3), we found that females did not differentiate between a striped and a nonstriped male. This result fits the observation that in many species, both males and females have stripes and that therefore the stripe patterns do not appear to matter in terms of mate choice. Other sensory modalities (e.g., olfaction or sound) might matter (more) in terms of mate choice but were not tested here.

The importance of the males’ nuptial coloration was highlighted in several studies on mate preference in cichlids (e.g., Maan et al., 2004; Seehausen, 1997; Seehausen & van Alphen, 1998) because it is often based on pigments such as carotenoids (red to yellow pigment) which most animals cannot synthesize *de novo* and thus have to be incorporated by diet. Thereby red/yellow coloration can have an important role in honest signaling of male quality and play an important part in sexual selection (Evans & Norris, 1996; Seehausen & van Alphen, 1998). Melanin, on the other hand, which is the pigment responsible for stripe patterns, can be synthesized *de novo* (Braasch et al., 2007) and thus may be a less informative indicator of male quality.

In summary, our results suggest that the correlated evolution between horizontal stripes and an elongated body shape is not the consequence of genetic linkage or pleiotropy, but most likely of correlational selection or a correlated response to selection. Since different elongation optima are supported for striped and nonstriped species, this trait correlation could result in an optimized optical illusion pattern. Such a pattern could potentially represent an adaptation to predator–prey interactions. Additionally, by taking advantage of a genetically modified striped CRISPR/Cas9 fish, we show that stripe patterns likely have no influence on mate preference. Therefore we suggest that the repeated evolution of stripe patterns in African cichlids has been predominantly driven by ecological selection.

## CONFLICT OF INTEREST

The authors declare that they have no competing interests.

## AUTHOR CONTRIBUTIONS

**Sabine Urban:** Formal analysis (lead); Visualization (lead); Writing – original draft (lead). **Jan Gerwin:** Data curation (equal); Writing – review & editing (supporting). **C. Darrin Hulsey:** Conceptualization (equal); Formal analysis (supporting); Funding acquisition (equal); Supervision (supporting); Writing – review & editing (supporting). **Axel Meyer:** Conceptualization (equal); Funding acquisition (equal); Writing – review & editing (supporting). **Claudius F. Kratochwil:** Conceptualization (equal); Funding acquisition (equal); Supervision (lead); Writing – review & editing (supporting).

## Supporting information

Supplementary Material

## Data Availability

Data are provided in manuscript and supplementary material.
