# Supplementary material for: The repeated evolution of stripe patterns is correlated with body morphology in the adaptive radiations of East African cichlid fishes
Source: Ecol Evol. 2022 Feb 7;12(2):e8568. doi: 10.1002/ece3.8568 (PMC8820146; doi:10.1002/ece3.8568)
Supplement: Supplementary file 1 — Supplementary Material [file ECE3-12-e8568-s001.docx]

**Supplementary Materials for**

**The repeated evolution of stripe patterns is correlated with body morphology in the adaptive radiations of East African cichlid fishes**

Sabine Urban, Jan Gerwin, C. Darrin Hulsey, Axel Meyer and Claudius F. Kratochwil

Correspondence to: claudius.kratochwil@helsinki.fi or axel.meyer@uni-konstanz.de

**Figure S1. Rooted supertree containing 461 species of African cichlids**. This tree combines information of the nuclear genome from 10 published phylogenies (Dunz and Schliewen, 2013; Hulsey et al., 2018; Hulsey et al., 2017; Hulsey et al., 2018; Hulsey et al., 2019; Irisarri et al., 2018; Kratochwil et al., 2018; Malinsky et al., 2018; McGee et al., 2016; Meier et al., 2017) as well as available information from the mitochondrial gene *nd2*.


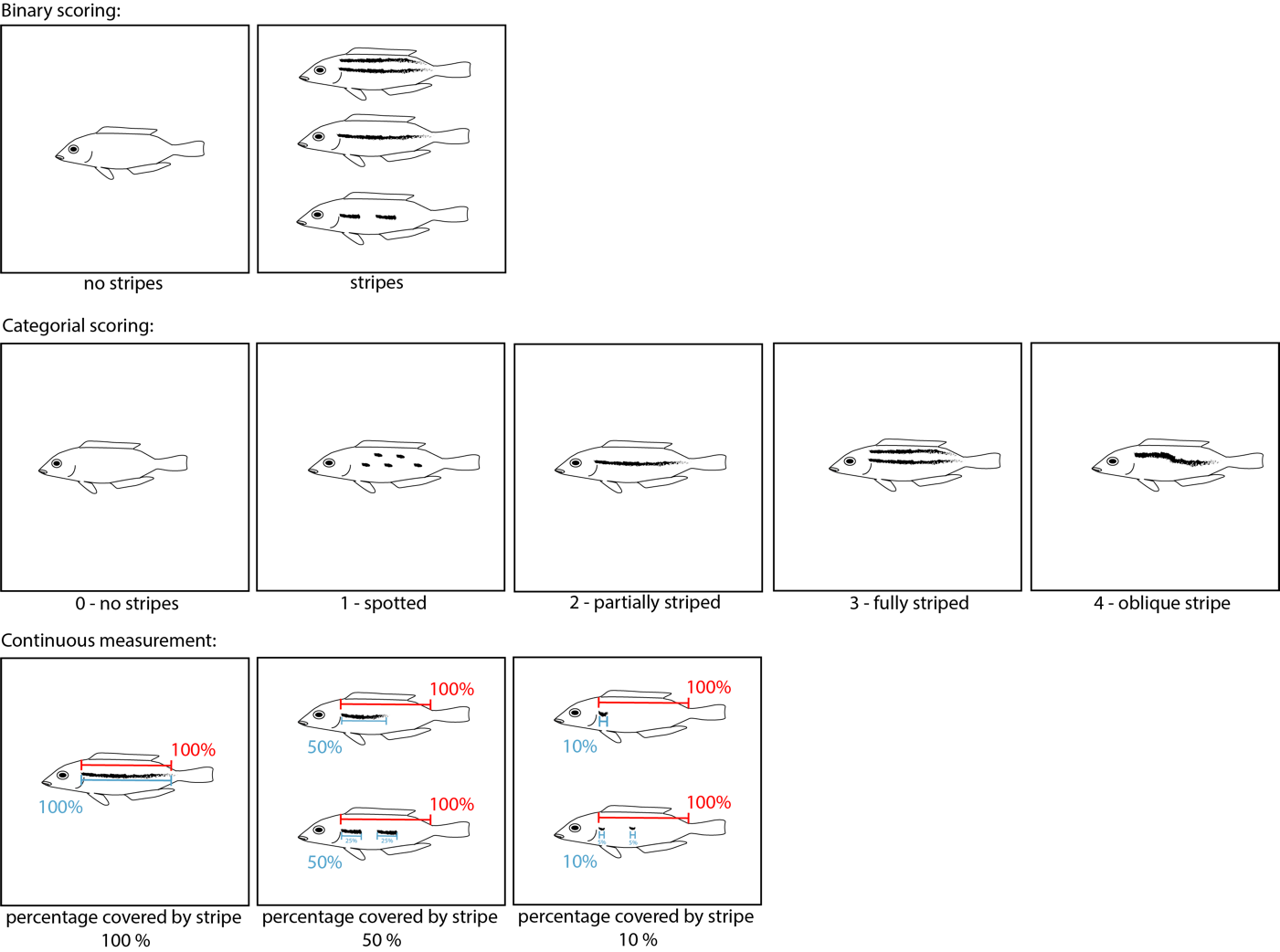


**Figure S2. Stripe measurement scheme**. To score the extend of stripe patterns three different methods were used. The binary measurement only differentiates between stripe absence (0 - no stripes) and presence (1 - stripes). The categorial scoring classifies the stripes into 5 different groups (0 - no stripes, 1 - spotted, 2 - partially striped, 3 - fully striped, 4 - oblique stripe). The continuous measurement gives the percentage of the midlateral stripe region covered by melanin.

**Table S1.** Phenotypic data of 461 species from all major East African cichlid lineages.

| **ID** | **Species** | **Current status (Eschmeyer 23/06/2020)** | **Tribus** | **Lake** | **dorsolateral stripe** | **midlateral stripe** | **any stripe** | **elongation index** | **percentage striped** | **stripe category** |
| --- | --- | --- | --- | --- | --- | --- | --- | --- | --- | --- |
|  |  |  |  |  | **0 = no; 1 = yes** | | |  |  |  |
| Alco_alca | Alcolapia alcalica | Oreochromis alcalicus | haplochromini | other | 0 | 0 | 0 | 3.62 | 0 | 0 |
| Alti_geof | Alticorpus geoffreyi | Alticorpus geoffreyi | haplochromini | malawi | 0 | 0 | 0 | 2.61 | 0 | 0 |
| Alti_macr | Alticorpus macrocleithrum | Alticorpus macrocleithrum | haplochromini | malawi | 0 | 0 | 0 | 2.6 | 0 | 0 |
| Alto_calv | Altolamprologus calvus | Altolamprologus calvus | haplochromini | tanganyika | 0 | 0 | 0 | 2.92 | 0 | 0 |
| Alto_comp | Altolamprologus compressiceps | Altolamprologus compressiceps | haplochromini | tanganyika | 0 | 0 | 0 | 2.47 | 0 | 0 |
| Aris_chri | Aristochromis christyi | Aristochromis christyi | haplochromini | malawi | 0 | 0 | 0 | 3.53 | 100 | 4 |
| Asta_allu | Astatoreochromis alluaudi | Astatoreochromis alluaudi | haplochromini | victoria | 0 | 0 | 0 | 2.98 | 0 | 0 |
| Asta_stra | Astatoreochromis straeleni | Astatoreochromis straeleni | haplochromini | tanganyika | 0 | 0 | 0 | 2.72 | 0 | 0 |
| Asta_bloy | Astatotilapia bloyeti | Astatotilapia bloyeti | haplochromini | other | 1 | 1 | 1 | 3.22 | 57 | 3 |
| Asta_burt | Astatotilapia burtoni | Astatotilapia burtoni | haplochromini | tanganyika | 1 | 1 | 1 | 2.54 | 77 | 3 |
| Asta_call | Astatotilapia calliptera | Astatotilapia calliptera | haplochromini | malawi | 0 | 0 | 0 | 2.57 | 0 | 0 |
| Asta_desf | Astatotilapia desfontainii | Astatotilapia desfontainii | haplochromini | other | 1 | 1 | 1 | 2.79 | 86 | 3 |
| Asta_flav | Astatotilapia flaviijosephi | Astatotilapia flaviijosephi | haplochromini | other | 1 | 1 | 1 | 2.5 | 74 | 3 |
| Asta_stap | Astatotilapia stappersii | Astatotilapia stappersii | haplochromini | victoria | 1 | 1 | 1 | 2.97 | 100 | 3 |
| Asta_twed | Astatotilapia tweddlei | Astatotilapia tweddlei | haplochromini | other | 0 | 0 | 0 | 3.1 | 0 | 0 |
| Aulo_baen | Aulonocara baenschi | Aulonocara baenschi | haplochromini | malawi | 0 | 0 | 0 | 3.03 | 0 | 0 |
| Aulo_hans | Aulonocara hansbaenschi | Aulonocara stuartgranti (synonym) | haplochromini | malawi | 0 | 0 | 0 | 2.7 | 0 | 0 |
| Aulo_jaco | Aulonocara jacobfreibergi | Aulonocara jacobfreibergi | haplochromini | malawi | 0 | 1 | 1 | 2.96 | 33 | 2 |
| Aulo_minu | Aulonocara sp. minutus | NA | haplochromini | malawi | 0 | 0 | 0 | 3.49 | 0 | 0 |
| Aulo_yell | Aulonocara sp. yellow | NA | haplochromini | malawi | 0 | 0 | 0 | 2.88 | 0 | 0 |
| Aulo_stev | Aulonocara steveni | Aulonocara stuartgranti (synonym) | haplochromini | malawi | 0 | 0 | 0 | 2.47 | 0 | 0 |
| Aulo_stua | Aulonocara stuartgranti | Aulonocara stuartgranti | haplochromini | malawi | 1 | 1 | 1 | 2.9 | 84 | 3 |
| Aulo_dewi | Aulonocranus dewindti | Aulonocranus dewindti | ectodini | tanganyika | 0 | 0 | 0 | 2.85 | 0 | 0 |
| Bail_cent | Baileychromis centropomoides | Baileychromis centropomoides | limnochromini | tanganyika | 0 | 0 | 0 | 5.14 | 0 | 0 |
| Bath_fasc | Bathybates fasciatus | Bathybates fasciatus | bathybatini | tanganyika | 0 | 1 | 1 | 4.61 | 100 | 2 |
| Bath_fero | Bathybates ferox | Bathybates ferox | bathybatini | tanganyika | 1 | 1 | 1 | 5.01 | 0 | 0 |
| Bath_grau | Bathybates graueri | Bathybates graueri | bathybatini | tanganyika | 1 | 1 | 1 | 3.83 | 65 | 2 |
| Bath_horn | Bathybates hornii | Bathybates hornii | bathybatini | tanganyika | 0 | 0 | 0 | 4.44 | 0 | 0 |
| Bath_leo | Bathybates leo | Bathybates leo | bathybatini | tanganyika | 1 | 1 | 1 | 4.52 | 78 | 2 |
| Bath_mino | Bathybates minor | Bathybates minor | bathybatini | tanganyika | 1 | 1 | 1 | 4.16 | 92 | 3 |
| Bath_vitt | Bathybates vittatus | Bathybates vittatus | bathybatini | tanganyika | 1 | 1 | 1 | 4.95 | 87 | 3 |
| Bent_hori | Benthochromis horii | Benthochromis horii | benthochromini | tanganyika | 0 | 0 | 0 | 3.33 | 0 | 0 |
| Bent_mela | Benthochromis melanoides | Benthochromis melanoides | benthochromini | tanganyika | 0 | 0 | 0 | 3.44 | 0 | 0 |
| Bent_tric | Benthochromis tricoti | Benthochromis tricoti | benthochromini | tanganyika | 1 | 1 | 1 | 4.03 | 98 | 3 |
| Boul_micr | Boulengerochromis microlepis | Boulengerochromis microlepis | boulengerochromini | tanganyika | 0 | 0 | 0 | 3.76 | 21 | 1 |
| Bucc_hete | Buccochromis heterotaenia | Buccochromis heterotaenia | haplochromini | malawi | 0 | 0 | 0 | 3.48 | 72 | 4 |
| Bucc_lept | Buccochromis lepturus | Buccochromis lepturus | haplochromini | malawi | 0 | 0 | 0 | 2.9 | 100 | 4 |
| Bucc_noto | Buccochromis nototaenia | Buccochromis nototaenia | haplochromini | malawi | 0 | 0 | 0 | 3.44 | 100 | 4 |
| Bucc_rhoa | Buccochromis rhoadesii | Buccochromis rhoadesii | haplochromini | malawi | 0 | 0 | 0 | 3.31 | 100 | 4 |
| Call_macr | Callochromis macrops | Callochromis macrops | ectodini | tanganyika | 1 | 0 | 1 | 3.19 | 29 | 2 |
| Call_pleu | Callochromis pleurospilus | Callochromis pleurospilus | ectodini | tanganyika | 0 | 0 | 0 | 3.54 | 0 | 0 |
| Card_scho | Cardiopharynx schoutedeni | Cardiopharynx schoutedeni | ectodini | tanganyika | 0 | 0 | 0 | 3.81 | 0 | 0 |
| Chal_bric | Chalinochromis brichardi | Chalinochromis brichardi | lamprologini | tanganyika | 0 | 0 | 0 | 3.33 | 0 | 0 |
| Chal_pope | Chalinochromis popelini | Chalinochromis popelini | lamprologini | tanganyika | 1 | 1 | 1 | 3.95 | 100 | 3 |
| Cham_caer | Champsochromis caeruleus | Champsochromis caeruleus | haplochromini | malawi | 0 | 0 | 0 | 4.26 | 100 | 4 |
| Cham_spil | Champsochromis spilorhynchus | Champsochromis spilorhynchus | haplochromini | malawi | 0 | 0 | 0 | 3.26 | 100 | 4 |
| Chei_euch | Cheilochromis euchilus | Cheilochromis euchilus | haplochromini | malawi | 1 | 1 | 1 | 2.88 | 100 | 3 |
| Chet_brev | Chetia brevicauda | Chetia brevicauda | haplochromini | other | 1 | 1 | 1 | 3.15 | 81 | 3 |
| Chet_brevis | Chetia brevis | Chetia brevis | haplochromini | other | 1 | 1 | 1 | 3.41 | 75 | 3 |
| Chet_flav | Chetia flaviventris | Chetia flaviventris | haplochromini | other | 0 | 1 | 1 | 3.49 | 100 | 3 |
| Chil_dupo | Chilochromis duponti | Chilochromis duponti | tilapiini | other | 1 | 1 | 1 | 3.02 | 40 | 2 |
| Chil_rhoa | Chilotilapia rhoadesii | Chilotilapia rhoadesii | haplochromini | malawi | 1 | 1 | 1 | 2.61 | 96 | 3 |
| Chro_gunt | Chromidotilapia guntheri | Chromidotilapia guntheri | chromidotilapiini | other | 1 | 1 | 1 | 3.02 | 100 | 3 |
| Coel_joka | Coelotilapia joka | Coelotilapia joka | coelotilapiini | other | 0 | 0 | 0 | 2.57 | 0 | 0 |
| Copa_borl | Copadichromis borleyi | Copadichromis borleyi | haplochromini | malawi | 0 | 0 | 0 | 2.67 | 25 | 1 |
| Copa_jack | Copadichromis jacksoni | Copadichromis jacksoni | haplochromini | malawi | 0 | 0 | 0 | 2.62 | 12 | 1 |
| Copa_mben | Copadichromis mbenjii | Copadichromis mbenjii | haplochromini | malawi | 0 | 0 | 0 | 2.87 | 31 | 1 |
| Copa_mlot | Copadichromis mloto | Copadichromis mloto | haplochromini | malawi | 0 | 0 | 0 | 3.76 | 0 | 0 |
| Copa_quad | Copadichromis quadrimaculatus | Copadichromis quadrimaculatus | haplochromini | malawi | 0 | 0 | 0 | 2.72 | 0 | 0 |
| Copa_trim | Copadichromis trimaculatus | Copadichromis trimaculatus | haplochromini | malawi | 0 | 0 | 0 | 2.76 | 25 | 1 |
| Copa_virg | Copadichromis virginalis | Copadichromis virginalis | haplochromini | malawi | 0 | 0 | 0 | 2.87 | 0 | 0 |
| Copt_coff | Coptodon coffea | Coptodon coffea | coptodonini | other | 0 | 0 | 0 | 2.24 | 0 | 0 |
| Copt_dage | Coptodon dageti | Coptodon dageti | coptodonini | other | 0 | 0 | 0 | 2.79 | 0 | 0 |
| Copt_disc | Coptodon discolor | Coptodon discolor | coptodonini | other | 0 | 0 | 0 | 2.54 | 0 | 0 |
| Copt_louk | Coptodon louka | Coptodon louka | coptodonini | other | 0 | 0 | 0 | 2.85 | 0 | 0 |
| Copt_nyon | Coptodon nyongana | Coptodon nyongana | coptodonini | other | 0 | 0 | 0 | 2.59 | 0 | 0 |
| Copt_rend | Coptodon rendalli | Coptodon rendalli | coptodonini | other | 0 | 0 | 0 | 2.35 | 0 | 0 |
| Copt_thol | Coptodon tholloni | Coptodon tholloni | coptodonini | other | 0 | 0 | 0 | 2.41 | 0 | 0 |
| Copt_walt | Coptodon walteri | Coptodon walteri | coptodonini | other | 0 | 0 | 0 | 2.09 | 0 | 0 |
| Copt_zill | Coptodon zillii | Coptodon zillii | coptodonini | other | 0 | 0 | 0 | 2.37 | 0 | 0 |
| Cten_hore | Ctenochromis horei | Ctenochromis horei | tropheini | tanganyika | 0 | 0 | 0 | 2.89 | 0 | 0 |
| Cten_pect | Ctenochromis pectoralis | Ctenochromis pectoralis | haplochromini | tanganyika | 0 | 0 | 0 | 2.98 | 0 | 0 |
| Cten_inte | Ctenopharynx intermedius | Ctenopharynx intermedius | haplochromini | malawi | 0 | 0 | 0 | 2.94 | 0 | 0 |
| Cten_niti | Ctenopharynx nitidus | Ctenopharynx nitidus | haplochromini | malawi | 0 | 0 | 0 | 2.96 | 0 | 1 |
| Cten_pict | Ctenopharynx pictus | Ctenopharynx pictus | haplochromini | malawi | 0 | 0 | 0 | 2.71 | 0 | 0 |
| Cunn_long | Cunningtonia longiventralis | Cunningtonia longiventralis | ectodini | tanganyika | 0 | 0 | 0 | 2.91 | 0 | 0 |
| Cyat_obli | Cyathochromis obliquidens | Cyathochromis obliquidens | haplochromini | malawi | 1 | 1 | 1 | 3.06 | 85 | 3 |
| Cyat_furc | Cyathopharynx furcifer | Cyathopharynx furcifer | ectodini | tanganyika | 0 | 0 | 0 | 3.16 | 0 | 0 |
| Cycl_schw | Cyclopharynx schwetzi | Cyclopharynx schwetzi | haplochromini | other | 0 | 0 | 0 | 2.84 | 0 | 0 |
| Cyno_afra | Cynotilapia afra | Cynotilapia afra | haplochromini | malawi | 0 | 0 | 0 | 2.9 | 0 | 0 |
| Cyno_axel | Cynotilapia axelrodi | Cynotilapia axelrodi | haplochromini | malawi | 0 | 0 | 0 | 3.24 | 0 | 0 |
| Cyph_fron | Cyphotilapia frontosa | Cyphotilapia frontosa | cyphotilapiini | tanganyika | 0 | 0 | 0 | 2.44 | 0 | 0 |
| Cyph_gibb | Cyphotilapia gibberosa | Cyphotilapia frontosa (synonym) | cyphotilapiini | tanganyika | 0 | 0 | 0 | 2.29 | 0 | 0 |
| Cypr_colo | Cyprichromis coloratus | Cyprichromis coloratus | cyprichromini | tanganyika | 0 | 0 | 0 | 3.94 | 0 | 0 |
| Cypr_lept | Cyprichromis leptosoma | Cyprichromis leptosoma | cyprichromini | tanganyika | 0 | 0 | 0 | 3.67 | 0 | 0 |
| Cypr_micr | Cyprichromis microlepidotus | Cyprichromis microlepidotus | cyprichromini | tanganyika | 0 | 0 | 0 | 4.18 | 0 | 0 |
| Cypr_pavo | Cyprichromis pavo | Cyprichromis pavo | cyprichromini | tanganyika | 0 | 0 | 0 | 4.23 | 0 | 0 |
| Cypr_zona | Cyprichromis zonatus | Cyprichromis zonatus | cyprichromini | tanganyika | 0 | 0 | 0 | 3.48 | 0 | 0 |
| Cyrt_moor | Cyrtocara moorii | Cyrtocara moorii | haplochromini | malawi | 0 | 0 | 0 | 3.14 | 0 | 1 |
| Dimi_comp | Dimidiochromis compressiceps | Dimidiochromis compressiceps | haplochromini | malawi | 1 | 1 | 1 | 3.72 | 100 | 3 |
| Dimi_dimi | Dimidiochromis dimidiatus | Dimidiochromis dimidiatus | haplochromini | malawi | 0 | 1 | 1 | 3.61 | 100 | 3 |
| Dimi_kiwi | Dimidiochromis kiwinge | Dimidiochromis kiwinge | haplochromini | malawi | 1 | 1 | 1 | 3.34 | 100 | 3 |
| Dimi_stri | Dimidiochromis strigatus | Dimidiochromis strigatus | haplochromini | malawi | 1 | 1 | 1 | 3.3 | 100 | 3 |
| Dipl_limn | Diplotaxodon limnothrissa | Diplotaxodon limnothrissa | haplochromini | malawi | 0 | 0 | 0 | 3.94 | 0 | 0 |
| Dipl_macr | Diplotaxodon macrops | Diplotaxodon macrops | haplochromini | malawi | 0 | 0 | 0 | 3.07 | 0 | 0 |
| Dipl_simi | Diplotaxodon sp. similis | NA | haplochromini | malawi | 0 | 0 | 0 | 2.94 | 0 | 0 |
| Doci_evel | Docimodus evelynae | Docimodus evelynae | haplochromini | malawi | 0 | 0 | 0 | 2.72 | 100 | 4 |
| Ecto_desc | Ectodus descampsii | Ectodus descampsii | ectodini | tanganyika | 0 | 0 | 0 | 3.66 | 0 | 0 |
| Eret_cyan | Eretmodus cyanostictus | Eretmodus cyanostictus | eretmodini | tanganyika | 0 | 0 | 0 | 3.02 | 0 | 0 |
| Eret_mark | Eretmodus marksmithi | Eretmodus marksmithi | eretmodini | tanganyika | 0 | 0 | 0 | 3.01 | 0 | 0 |
| Etia_ngut | Etia nguti | Etia nguti | etiini | other | 0 | 0 | 0 | 2.53 | 38 | 2 |
| Foss_rost | Fossorochromis rostratus | Fossorochromis rostratus | haplochromini | malawi | 1 | 1 | 1 | 3.17 | 54 | 2 |
| Geny_ment | Genyochromis mento | Genyochromis mento | haplochromini | malawi | 0 | 0 | 0 | 3.61 | 0 | 0 |
| Gnat_perm | Gnathochromis permaxillaris | Gnathochromis permaxillaris | limnochromini | tanganyika | 0 | 0 | 0 | 3.51 | 0 | 0 |
| Gnat_pfef | Gnathochromis pfefferi | Gnathochromis pfefferi | limnochromini | tanganyika | 0 | 0 | 0 | 3.13 | 0 | 0 |
| Gobi_ethe | Gobiocichla ethelwynnae | Gobiocichla ethelwynnae | gobiocichlini | other | 1 | 1 | 1 | 7.09 | 100 | 3 |
| Gobi_wond | Gobiocichla wonderi | Gobiocichla wonderi | gobiocichlini | other | 1 | 1 | 1 | 6.98 | 100 | 3 |
| Gram_lema | Grammatotria lemairii | Grammatotria lemairii | ectodini | tanganyika | 0 | 0 | 0 | 4 | 0 | 0 |
| Gree_bell | Greenwoodochromis bellcrossi | Greenwoodochromis bellcrossi | limnochromini | tanganyika | 0 | 0 | 0 | 3.29 | 0 | 1 |
| Gree_chri | Greenwoodochromis christyi | Greenwoodochromis christyi | limnochromini | tanganyika | 0 | 0 | 0 | 2.97 | 0 | 1 |
| Hapl_aene | Haplochromis aeneocolor | Haplochromis aeneocolor | haplochromini | victoria | 0 | 1 | 1 | 2.94 | 53 | 2 |
| Hapl_chil | Haplochromis chilotes | Haplochromis chilotes | haplochromini | victoria | 1 | 1 | 1 | 3.13 | 100 | 3 |
| Hapl_dege | Haplochromis degeni | Haplochromis degeni | haplochromini | victoria | 0 | 1 | 1 | 2.95 | 100 | 3 |
| Hapl_eleg | Haplochromis elegans | Haplochromis elegans | haplochromini | victoria | 0 | 0 | 0 | 2.84 | 0 | 0 |
| Hapl_fisc | Haplochromis fischeri | Haplochromis fischeri | haplochromini | victoria | 1 | 1 | 1 | 3.04 | 100 | 3 |
| Hapl_grac | Haplochromis gracilior | Haplochromis gracilior | haplochromini | victoria | 0 | 0 | 0 | 3.23 | 0 | 0 |
| Hapl_insi | Haplochromis insidiae | Haplochromis insidiae | haplochromini | victoria | 0 | 1 | 1 | 3.17 | 52 | 2 |
| Hapl_lati | Haplochromis latifasciatus | Haplochromis latifasciatus | haplochromini | victoria | 0 | 0 | 0 | 2.98 | 0 | 0 |
| Hapl_macr | Haplochromis macrops | Haplochromis macrops | haplochromini | victoria | 0 | 0 | 0 | 2.8 | 0 | 0 |
| Hapl_mela | Haplochromis melanopterus | Haplochromis melanopterus | haplochromini | victoria | 0 | 0 | 0 | 3.07 | 0 | 0 |
| Hapl_micr | Haplochromis microchrysomelas | Haplochromis microchrysomelas | haplochromini | other | 0 | 0 | 0 | 2.84 | 0 | 0 |
| Hapl_nubi | Haplochromis nubilus | Haplochromis nubilus | haplochromini | victoria | 1 | 1 | 1 | 2.84 | 100 | 3 |
| Hapl_obli | Haplochromis obliquidens | Haplochromis obliquidens | haplochromini | victoria | 0 | 0 | 0 | 3.2 | 0 | 0 |
| Hapl_palu | Haplochromis paludinosus | Astatotilapia paludinosa | haplochromini | victoria | 0 | 0 | 0 | 3.02 | 0 | 0 |
| Hapl_phyt | Haplochromis phytophagus | Haplochromis phytophagus | haplochromini | victoria | 1 | 1 | 1 | 2.86 | 100 | 3 |
| Hapl_pyrr | Haplochromis pyrrhocephalus | Haplochromis pyrrhocephalus | haplochromini | victoria | 0 | 0 | 0 | 3.24 | 0 | 0 |
| Hapl_rudo | Haplochromis rudolfianus | Haplochromis rudolfianus | haplochromini | victoria | 0 | 0 | 0 | 3.42 | 0 | 0 |
| Hapl_sauv | Haplochromis sauvagei | Haplochromis sauvagei | haplochromini | victoria | 1 | 1 | 1 | 3.25 | 0 | 3 |
| Hapl_serr | Haplochromis serranus | Haplochromis serranus | haplochromini | victoria | 1 | 1 | 1 | 3.56 | 0 | 3 |
| Hapl_snoe | Haplochromis snoeksi | Haplochromis snoeksi | haplochromini | victoria | 0 | 0 | 0 | 3.28 | 0 | 0 |
| Hapl_squa | Haplochromis squamipinnis | Haplochromis squamipinnis | haplochromini | victoria | 0 | 0 | 0 | 3.25 | 0 | 0 |
| Hapl_ther | Haplochromis thereuterion | Haplochromis thereuterion | haplochromini | victoria | 1 | 1 | 1 | 4.08 | 100 | 3 |
| Hapl_vitt | Haplochromis vittatus | Haplochromis vittatus | haplochromini | victoria | 1 | 1 | 1 | 3.36 | 0 | 3 |
| Hapl_trif | Haplotaxodon trifasciatus | Haplotaxodon microlepis (synonym) | perissodini | tanganyika | 0 | 0 | 0 | 3.51 | 0 | 1 |
| Hemi_sten | Hemibates stenosoma | Hemibates stenosoma | bathybatini | tanganyika | 1 | 1 | 1 | 3.16 | 73 | 2 |
| Hemi_cera | Hemichromis cerasogaster | Hemichromis cerasogaster | hemichromini | other | 0 | 0 | 0 | 2.67 | 0 | 0 |
| Hemi_elon | Hemichromis elongatus | Hemichromis elongatus | hemichromini | other | 0 | 0 | 0 | 2.34 | 0 | 0 |
| Hemi_oxyr | Hemitilapia oxyrhyncha | Hemitilapia oxyrhynchus | haplochromini | malawi | 0 | 0 | 0 | 3.09 | 0 | 1 |
| Hete_mult | Heterochromis multidens | Heterochromis multidens | heterochromidini | other | 0 | 1 | 1 | 2.23 | 100 | 3 |
| Hete_butt | Heterotilapia buttikoferi | Heterotilapia buttikoferi | heterotilapiini | other | 0 | 0 | 0 | 2.2 | 0 | 0 |
| Hete_cess | Heterotilapia cessiana | Heterotilapia cessiana | heterotilapiini | other | 0 | 0 | 0 | 2.16 | 0 | 0 |
| Inte_looc | Interochromis loocki | Interochromis loocki | tropheini | tanganyika | 1 | 0 | 1 | 2.86 | 100 | 2 |
| Iodo_spre | Iodotropheus sprengerae | Iodotropheus sprengerae | haplochromini | malawi | 0 | 0 | 0 | 2.72 | 0 | 0 |
| Juli_dick | Julidochromis dickfeldi | Julidochromis dickfeldi | lamprologini | tanganyika | 1 | 1 | 1 | 4.41 | 100 | 3 |
| Juli_marl | Julidochromis marlieri | Julidochromis marlieri | lamprologini | tanganyika | 1 | 1 | 1 | 3.92 | 100 | 3 |
| Juli_orna | Julidochromis ornatus | Julidochromis ornatus | lamprologini | tanganyika | 1 | 1 | 1 | 4.66 | 87 | 3 |
| Juli_rega | Julidochromis regani | Julidochromis regani | lamprologini | tanganyika | 1 | 1 | 1 | 4.31 | 100 | 3 |
| Juli_tran | Julidochromis transcriptus | Julidochromis transcriptus | lamprologini | tanganyika | 1 | 1 | 1 | 4.13 | 77 | 3 |
| Koni_diku | Konia dikume | Konia dikume | oreochromini | other | 1 | 1 | 1 | 3.06 | 27 | 2 |
| Koni_eise | Konia eisentrauti | Konia eisentrauti | oreochromini | other | 0 | 1 | 1 | 2.96 | 60 | 3 |
| Labe_fuel | Labeotropheus fuelleborni | Labeotropheus fuelleborni | haplochromini | malawi | 0 | 1 | 1 | 3.15 | 79 | 3 |
| Labe_trew | Labeotropheus trewavasae | Labeotropheus trewavasae | haplochromini | malawi | 0 | 0 | 0 | 2.84 | 0 | 0 |
| Labi_caer | Labidochromis caeruleus | Labidochromis caeruleus | haplochromini | malawi | 0 | 0 | 0 | 2.81 | 0 | 0 |
| Labi_giga | Labidochromis gigas | Labidochromis gigas | haplochromini | malawi | 0 | 0 | 0 | 2.66 | 0 | 0 |
| Labi_iant | Labidochromis ianthinus | Labidochromis ianthinus | haplochromini | malawi | 0 | 0 | 0 | 2.71 | 0 | 0 |
| Labi_mylo | Labidochromis mylodon | Labidochromis mylodon | haplochromini | malawi | 0 | 0 | 0 | 3.38 | 0 | 0 |
| Labi_pall | Labidochromis pallidus | Labidochromis pallidus | haplochromini | malawi | 0 | 0 | 0 | 3.23 | 0 | 0 |
| Labi_vell | Labidochromis vellicans | Labidochromis vellicans | haplochromini | malawi | 1 | 1 | 1 | 2.95 | 79 | 3 |
| Lamp_call | Lamprologus callipterus | Lamprologus callipterus | lamprologini | tanganyika | 0 | 0 | 0 | 3.58 | 0 | 0 |
| Lamp_cong | Lamprologus congoensis | Lamprologus congoensis | lamprologini | other | 0 | 0 | 0 | 4.36 | 0 | 0 |
| Lamp_kung | Lamprologus kungweensis | Neolamprologus kungweensis | lamprologini | tanganyika | 0 | 0 | 0 | 4.03 | 0 | 0 |
| Lamp_lapa | Lamprologus laparogramma | Neolamprologus laparogramma | lamprologini | tanganyika | 0 | 0 | 0 | 4.77 | 0 | 0 |
| Lamp_lema | Lamprologus lemairii | Lamprologus lemairii | lamprologini | tanganyika | 0 | 0 | 0 | 3.3 | 0 | 0 |
| Lamp_mele | Lamprologus meleagris | Lamprologus stappersi (synonym) | lamprologini | tanganyika | 0 | 1 | 1 | 3.95 | 100 | 3 |
| Lamp_mocq | Lamprologus mocquardi | Lamprologus mocquardi | lamprologini | other | 0 | 0 | 0 | 4.42 | 0 | 0 |
| Lamp_ocel | Lamprologus ocellatus | Lamprologus ocellatus | lamprologini | tanganyika | 0 | 0 | 0 | 3.39 | 0 | 0 |
| Lamp_orna | Lamprologus ornatipinnis | Lamprologus ornatipinnis | lamprologini | tanganyika | 1 | 1 | 1 | 3.69 | 100 | 3 |
| Lamp_sign | Lamprologus signatus | Neolamprologus signatus | lamprologini | tanganyika | 0 | 0 | 0 | 3.78 | 0 | 0 |
| Lamp_spec | Lamprologus speciosus | Lamprologus speciosus | lamprologini | tanganyika | 0 | 0 | 0 | 3.67 | 0 | 0 |
| Lamp_teug | Lamprologus teugelsi | Lamprologus teugelsi | lamprologini | other | 0 | 0 | 0 | 5.08 | 0 | 0 |
| Lamp_tigr | Lamprologus tigripictilis | Lamprologus tigripictilis | lamprologini | other | 0 | 0 | 0 | 4.26 | 0 | 0 |
| Lamp_wern | Lamprologus werneri | Lamprologus werneri | lamprologini | other | 0 | 1 | 1 | 3.67 | 62 | 2 |
| Lepi_atte | Lepidiolamprologus attenuatus | Lepidiolamprologus attenuatus | lamprologini | tanganyika | 0 | 1 | 1 | 4.63 | 0 | 1 |
| Lepi_boul | Lepidiolamprologus boulengeri | Lepidiolamprologus boulengeri | lamprologini | tanganyika | 0 | 0 | 0 | 3.27 | 0 | 0 |
| Lepi_cunn | Lepidiolamprologus cunningtoni | Neolamprologus cunningtoni | lamprologini | tanganyika | 0 | 0 | 0 | 4 | 0 | 0 |
| Lepi_elon | Lepidiolamprologus elongatus | Lepidiolamprologus elongatus | lamprologini | tanganyika | 1 | 1 | 1 | 3.59 | 27 | 2 |
| Lepi_hecq | Lepidiolamprologus hecqui | Lepidiolamprologus hecqui | lamprologini | tanganyika | 0 | 0 | 0 | 3.4 | 0 | 1 |
| Lepi_kend | Lepidiolamprologus kendalli | Lepidiolamprologus kendalli | lamprologini | tanganyika | 1 | 1 | 1 | 4.41 | 93 | 3 |
| Lepi_meel | Lepidiolamprologus meeli | Lepidiolamprologus meeli | lamprologini | tanganyika | 0 | 0 | 0 | 3.45 | 0 | 0 |
| Lepi_nkam | Lepidiolamprologus nkambae | Lepidiolamprologus kendalli (snonym) | lamprologini | tanganyika | 1 | 1 | 1 | 4.68 | 83 | 3 |
| Lepi_prof | Lepidiolamprologus profundicola | Lepidiolamprologus profundicola | lamprologini | tanganyika | 0 | 0 | 0 | 3.67 | 53 | 2 |
| Lepi_vari | Lepidiolamprologus variostigma | Neolamprologus variostigma | lamprologini | tanganyika | 0 | 0 | 0 | 3.78 | 0 | 0 |
| Lest_pers | Lestradea perspicax | Lestradea perspicax | ectodini | tanganyika | 0 | 0 | 0 | 4.03 | 0 | 0 |
| Lest_stap | Lestradea stappersii | Lestradea perspicax (synonym) | ectodini | tanganyika | 0 | 0 | 0 | 4.07 | 0 | 0 |
| Leth_albu | Lethrinops albus | Lethrinops albus | haplochromini | malawi | 0 | 0 | 0 | 3.2 | 0 | 0 |
| Leth_auri | Lethrinops auritus | Lethrinops auritus | haplochromini | malawi | 0 | 0 | 0 | 2.72 | 0 | 0 |
| Leth_furc | Lethrinops furcifer | Lethrinops furcifer | haplochromini | malawi | 0 | 0 | 0 | 2.46 | 0 | 0 |
| Leth_goss | Lethrinops gossei | Lethrinops gossei | haplochromini | malawi | 0 | 0 | 0 | 2.11 | 0 | 0 |
| Leth_leth | Lethrinops lethrinus | Lethrinops lethrinus | haplochromini | malawi | 1 | 1 | 1 | 3.28 | 88 | 3 |
| Leth_longp | Lethrinops longipinnis | Lethrinops longipinnis | haplochromini | malawi | 0 | 0 | 0 | 2.48 | 0 | 0 |
| Leth_marg | Lethrinops marginatus | Lethrinops marginatus | haplochromini | malawi | 0 | 0 | 0 | 2.93 | 0 | 0 |
| Leth_oliv | Lethrinops sp. oliveri | NA | haplochromini | malawi | 0 | 0 | 0 | 2.36 | 0 | 0 |
| Limn_abee | Limnochromis abeelei | Greenwoodochromis abeelei | limnochromini | tanganyika | 0 | 0 | 0 | 2.84 | 0 | 0 |
| Limn_auri | Limnochromis auritus | Limnochromis auritus | limnochromini | tanganyika | 0 | 0 | 0 | 3.09 | 0 | 0 |
| Limn_dard | Limnochromis dardennii | Limnotilapia dardennii | limnochromini | tanganyika | 0 | 0 | 0 | 2.96 | 41 | 2 |
| Limn_stan | Limnochromis staneri | Greenwoodochromis staneri | limnochromini | tanganyika | 0 | 0 | 0 | 2.81 | 0 | 0 |
| Lobo_labi | Lobochilotes labiatus | Lobochilotes labiata | tropheini | tanganyika | 0 | 0 | 0 | 2.89 | 0 | 0 |
| Mayl_auro | Maylandia aurora | Maylandia aurora | haplochromini | malawi | 0 | 0 | 0 | 2.96 | 0 | 0 |
| Mayl_bene | Maylandia benetos | Maylandia benetos | haplochromini | malawi | 0 | 0 | 0 | 2.98 | 0 | 0 |
| Mayl_call | Maylandia callainos | Maylandia callainos | haplochromini | malawi | 0 | 0 | 0 | 2.57 | 0 | 0 |
| Mayl_chry | Maylandia chrysomallos | Maylandia chrysomallos | haplochromini | malawi | 0 | 0 | 0 | 2.9 | 0 | 0 |
| Mayl_esth | Maylandia estherae | Maylandia estherae | haplochromini | malawi | 0 | 0 | 0 | 2.68 | 0 | 0 |
| Mayl_gres | Maylandia greshakei | Maylandia greshakei | haplochromini | malawi | 0 | 0 | 0 | 2.66 | 0 | 0 |
| Mayl_hete | Maylandia heteropicta | Chindongo heteropictus | haplochromini | malawi | 0 | 0 | 0 | 2.76 | 0 | 0 |
| Mayl_lani | Maylandia lanisticola | Maylandia lanisticola | haplochromini | malawi | 1 | 0 | 1 | 2.97 | 0 | 0 |
| Mayl_lomb | Maylandia lombardoi | Maylandia lombardoi | haplochromini | malawi | 0 | 0 | 0 | 2.86 | 0 | 0 |
| Mayl_mben | Maylandia mbenjii | Maylandia mbenjii | haplochromini | malawi | 0 | 0 | 0 | 2.63 | 0 | 0 |
| Mayl_pulp | Maylandia pulpican | Maylandia pulpican | haplochromini | malawi | 0 | 0 | 0 | 2.71 | 0 | 0 |
| Mayl_xans | Maylandia xanstomachus | Maylandia xanstomachus | haplochromini | malawi | 0 | 0 | 0 | 2.59 | 0 | 0 |
| Mayl_zebr | Maylandia zebra | Maylandia zebra | haplochromini | malawi | 0 | 0 | 0 | 3.07 | 0 | 0 |
| Mche_euci | Mchenga eucinostomus | Mchenga eucinostomus | haplochromini | malawi | 0 | 0 | 0 | 3.27 | 0 | 0 |
| Mela_aura | Melanochromis auratus | Melanochromis auratus | haplochromini | malawi | 1 | 1 | 1 | 3.57 | 100 | 3 |
| Mela_kask | Melanochromis kaskazini | Melanochromis kaskazini | haplochromini | malawi | 1 | 1 | 1 | 3.57 | 100 | 3 |
| Mela_lori | Melanochromis loriae | Melanochromis loriae | haplochromini | malawi | 1 | 1 | 1 | 3.13 | 100 | 3 |
| Mela_mela | Melanochromis melanopterus | Melanochromis melanopterus | haplochromini | malawi | 1 | 1 | 1 | 3.67 | 100 | 3 |
| Mela_verm | Melanochromis vermivorus | Melanochromis vermivorus | haplochromini | malawi | 1 | 1 | 1 | 3.15 | 100 | 3 |
| Myak_myak | Myaka myaka | Myaka myaka | tilapiini | other | 0 | 0 | 0 | 2.93 | 0 | 0 |
| Mylo_anap | Mylochromis anaphyrmus | Mylochromis anaphyrmus | haplochromini | malawi | 0 | 0 | 0 | 2.46 | 73 | 4 |
| Mylo_epic | Mylochromis epichorialis | Mylochromis epichorialis | haplochromini | malawi | 0 | 0 | 0 | 2.84 | 100 | 4 |
| Mylo_eric | Mylochromis ericotaenia | Mylochromis ericotaenia | haplochromini | malawi | 0 | 0 | 0 | 3.42 | 50 | 4 |
| Mylo_inco | Mylochromis incola | Mylochromis incola | haplochromini | malawi | 0 | 0 | 0 | 2.96 | 100 | 4 |
| Mylo_mola | Mylochromis mola | Mylochromis mola | haplochromini | malawi | 0 | 0 | 0 | 3.18 | 95 | 4 |
| Nano_pari | Nanochromis parilus | Nanochromis parilus | chromidotilapiini | other | 0 | 0 | 0 | 3.86 | 0 | 0 |
| Neol_bifa | Neolamprologus bifasciatus | Neolamprologus bifasciatus | lamprologini | tanganyika | 1 | 1 | 1 | 3.46 | 100 | 3 |
| Neol_brev | Neolamprologus brevis | Lamprologus brevis | lamprologini | tanganyika | 0 | 0 | 0 | 3.04 | 0 | 0 |
| Neol_bric | Neolamprologus brichardi | Neolamprologus pulcher (synonym) | lamprologini | tanganyika | 0 | 0 | 0 | 3.37 | 0 | 0 |
| Neol_bues | Neolamprologus buescheri | Neolamprologus buescheri | lamprologini | tanganyika | 1 | 1 | 1 | 4.43 | 100 | 3 |
| Neol_caud | Neolamprologus caudopunctatus | Neolamprologus caudopunctatus | lamprologini | tanganyika | 0 | 0 | 0 | 3.96 | 0 | 0 |
| Neol_chri | Neolamprologus christyi | Neolamprologus christyi | lamprologini | tanganyika | 0 | 0 | 0 | 3.66 | 0 | 0 |
| Neol_cyli | Neolamprologus cylindricus | Neolamprologus cylindricus | lamprologini | tanganyika | 0 | 0 | 0 | 4.94 | 0 | 0 |
| Neol_devo | Neolamprologus devosi | Neolamprologus devosi | lamprologini | tanganyika | 0 | 0 | 0 | 3.76 | 0 | 0 |
| Neol_falc | Neolamprologus falcicula | Neolamprologus falcicula | lamprologini | tanganyika | 0 | 0 | 0 | 2.8 | 0 | 0 |
| Neol_fasc | Neolamprologus fasciatus | Altolamprologus fasciatus | lamprologini | tanganyika | 0 | 0 | 0 | 4.17 | 0 | 0 |
| Neol_furc | Neolamprologus furcifer | Neolamprologus furcifer | lamprologini | tanganyika | 1 | 1 | 1 | 3.82 | 67 | 2 |
| Neol_grac | Neolamprologus gracilis | Neolamprologus gracilis | lamprologini | tanganyika | 0 | 0 | 0 | 3.88 | 0 | 0 |
| Neol_heli | Neolamprologus helianthus | Neolamprologus splendens (synonym) | lamprologini | tanganyika | 0 | 0 | 0 | 3.16 | 0 | 0 |
| Neol_lele | Neolamprologus leleupi | Neolamprologus leleupi | lamprologini | tanganyika | 0 | 0 | 0 | 3.62 | 0 | 0 |
| Neol_lelo | Neolamprologus leloupi | Neolamprologus leloupi | lamprologini | tanganyika | 0 | 0 | 0 | 3.29 | 0 | 0 |
| Neol_long | Neolamprologus longicaudatus | Neolamprologus longicaudatus | lamprologini | tanganyika | 1 | 1 | 1 | 4.2 | 90 | 3 |
| Neol_longi | Neolamprologus longior | Neolamprologus longior | lamprologini | tanganyika | 0 | 0 | 0 | 3.89 | 0 | 0 |
| Neol_maru | Neolamprologus marunguensis | Neolamprologus marunguensis | lamprologini | tanganyika | 0 | 0 | 0 | 2.94 | 0 | 0 |
| Neol_mode | Neolamprologus modestus | Neolamprologus modestus | lamprologini | tanganyika | 0 | 0 | 0 | 3.21 | 0 | 0 |
| Neol_mond | Neolamprologus mondabu | Neolamprologus mondabu | lamprologini | tanganyika | 0 | 0 | 0 | 3.23 | 0 | 0 |
| Neol_mult | Neolamprologus multifasciatus | Lamprologus multifasciatus | lamprologini | tanganyika | 0 | 0 | 0 | 3.22 | 0 | 0 |
| Neol_must | Neolamprologus mustax | Neolamprologus mustax | lamprologini | tanganyika | 0 | 0 | 0 | 3.28 | 0 | 0 |
| Neol_nige | Neolamprologus niger | Neolamprologus niger | lamprologini | tanganyika | 0 | 0 | 0 | 2.74 | 0 | 0 |
| Neol_nigr | Neolamprologus nigriventris | Neolamprologus nigriventris | lamprologini | tanganyika | 0 | 0 | 0 | 4.43 | 0 | 0 |
| Neol_obsc | Neolamprologus obscurus | Neolamprologus obscurus | lamprologini | tanganyika | 0 | 0 | 0 | 3 | 0 | 0 |
| Neol_oliv | Neolamprologus olivaceous | Neolamprologus pulcher (synonym) | lamprologini | tanganyika | 0 | 0 | 0 | 2.72 | 0 | 1 |
| Neol_pect | Neolamprologus pectoralis | Neolamprologus pectoralis | lamprologini | tanganyika | 0 | 0 | 0 | 4.22 | 0 | 0 |
| Neol_petr | Neolamprologus petricola | Neolamprologus petricola | lamprologini | tanganyika | 0 | 0 | 0 | 2.91 | 0 | 0 |
| Neol_proc | Neolamprologus prochilus | Neolamprologus prochilus | lamprologini | tanganyika | 0 | 0 | 0 | 3.3 | 61 | 2 |
| Neol_pulc | Neolamprologus pulcher | Neolamprologus pulcher | lamprologini | tanganyika | 0 | 0 | 0 | 3.48 | 0 | 0 |
| Neol_savo | Neolamprologus savoryi | Neolamprologus savoryi | lamprologini | tanganyika | 0 | 0 | 0 | 2.65 | 0 | 0 |
| Neol_sexf | Neolamprologus sexfasciatus | Neolamprologus sexfasciatus | lamprologini | tanganyika | 0 | 0 | 0 | 3.13 | 0 | 1 |
| Neol_simi | Neolamprologus similis | Neolamprologus similis | lamprologini | tanganyika | 0 | 0 | 0 | 3.1 | 0 | 0 |
| Neol_sple | Neolamprologus splendens | Neolamprologus splendens | lamprologini | tanganyika | 0 | 0 | 0 | 3.42 | 0 | 0 |
| Neol_tetr | Neolamprologus tetracanthus | Neolamprologus tetracanthus | lamprologini | tanganyika | 0 | 0 | 0 | 3.46 | 0 | 0 |
| Neol_timi | Neolamprologus timidus | Neolamprologus timidus | lamprologini | tanganyika | 1 | 1 | 1 | 3.95 | 68 | 3 |
| Neol_toae | Neolamprologus toae | Neolamprologus toae | lamprologini | tanganyika | 0 | 0 | 0 | 2.71 | 0 | 0 |
| Neol_tret | Neolamprologus tretocephalus | Neolamprologus tretocephalus | lamprologini | tanganyika | 0 | 0 | 0 | 3.15 | 0 | 0 |
| Neol_vent | Neolamprologus ventralis | Neolamprologus ventralis | lamprologini | tanganyika | 0 | 0 | 0 | 3.59 | 0 | 0 |
| Neol_walt | Neolamprologus walteri | Neolamprologus walteri | lamprologini | tanganyika | 0 | 0 | 0 | 3.83 | 0 | 0 |
| Neol_waut | Neolamprologus wauthioni | Neolamprologus wauthioni | lamprologini | tanganyika | 0 | 0 | 0 | 4.02 | 0 | 0 |
| Nimb_fusc | Nimbochromis fuscotaeniatus | Nimbochromis fuscotaeniatus | haplochromini | malawi | 1 | 1 | 1 | 3.54 | 100 | 3 |
| Nimb_linn | Nimbochromis linni | Nimbochromis linni | haplochromini | malawi | 0 | 0 | 0 | 3.26 | 0 | 1 |
| Nimb_livi | Nimbochromis livingstoni | Nimbochromis livingstoni | haplochromini | malawi | 1 | 1 | 1 | 3.1 | 90 | 3 |
| Nimb_poly | Nimbochromis polystigma | Nimbochromis polystigma | haplochromini | malawi | 1 | 1 | 1 | 2.76 | 82 | 3 |
| Nimb_venu | Nimbochromis venustus | Nimbochromis venustus | haplochromini | malawi | 0 | 0 | 0 | 2.47 | 0 | 0 |
| Nyas_pros | Nyassachromis prostoma | Nyassachromis prostoma | haplochromini | malawi | 0 | 1 | 1 | 3.57 | 100 | 3 |
| Opht_boop | Ophthalmotilapia boops | Ophthalmotilapia boops | ectodini | tanganyika | 0 | 0 | 0 | 3.33 | 0 | 0 |
| Opht_hete | Ophthalmotilapia heterodonta | Ophthalmotilapia heterodonta | ectodini | tanganyika | 0 | 0 | 0 | 3.43 | 0 | 0 |
| Opht_nasu | Ophthalmotilapia nasuta | Ophthalmotilapia nasuta | ectodini | tanganyika | 0 | 0 | 0 | 3.42 | 0 | 0 |
| Opht_vent | Ophthalmotilapia ventralis | Ophthalmotilapia ventralis | ectodini | tanganyika | 0 | 0 | 0 | 3.38 | 0 | 0 |
| Oreo_amph | Oreochromis amphimelas | Oreochromis amphimelas | oreochromini | other | 0 | 0 | 0 | 3.23 | 0 | 1 |
| Oreo_ande | Oreochromis andersonii | Oreochromis andersonii | oreochromini | other | 0 | 0 | 0 | 2.77 | 0 | 0 |
| Oreo_aure | Oreochromis aureus | Oreochromis aureus | oreochromini | other | 0 | 0 | 0 | 2.34 | 0 | 0 |
| Oreo_chun | Oreochromis chungruruensis | Oreochromis chungruruensis | oreochromini | other | 0 | 0 | 0 | 2.51 | 0 | 0 |
| Oreo_escu | Oreochromis esculentus | Oreochromis esculentus | oreochromini | victoria | 1 | 1 | 1 | 3.03 | 100 | 3 |
| Oreo_karo | Oreochromis karomo | Oreochromis karomo | oreochromini | other | 0 | 1 | 1 | 2.65 | 40 | 2 |
| Oreo_leuc | Oreochromis leucostictus | Oreochromis leucostictus | oreochromini | other | 0 | 0 | 0 | 2.45 | 0 | 0 |
| Oreo_macr | Oreochromis macrochir | Oreochromis macrochir | oreochromini | other | 0 | 0 | 0 | 2.63 | 0 | 0 |
| Oreo_moss | Oreochromis mossambicus | Oreochromis mossambicus | oreochromini | other | 1 | 1 | 1 | 2.74 | 67 | 3 |
| Oreo_mwer | Oreochromis mweruensis | Oreochromis mweruensis | oreochromini | other | 0 | 0 | 0 | 2.03 | 0 | 0 |
| Oreo_nilo | Oreochromis niloticus | Oreochromis niloticus | oreochromini | other | 0 | 0 | 0 | 2.29 | 0 | 0 |
| Oreo_schw | Oreochromis schwebischi | Oreochromis schwebischi | oreochromini | other | 0 | 1 | 1 | 2.49 | 55 | 2 |
| Oreo_shir | Oreochromis shiranus | Oreochromis shiranus | oreochromini | other | 0 | 0 | 0 | 3.09 | 0 | 0 |
| Oreo_squa | Oreochromis squamipinnis | Oreochromis squamipinnis | oreochromini | malawi | 0 | 0 | 0 | 2.57 | 0 | 0 |
| Oreo_tang | Oreochromis tanganicae | Oreochromis tanganicae | oreochromini | tanganyika | 1 | 1 | 1 | 2.48 | 93 | 3 |
| Oreo_urol | Oreochromis urolepis | Oreochromis urolepis | oreochromini | other | 1 | 1 | 1 | 2.8 | 100 | 3 |
| Oreo_vari | Oreochromis variabilis | Oreochromis variabilis | oreochromini | victoria | 0 | 0 | 0 | 2.32 | 0 | 0 |
| Orth_kalu | Orthochromis kalungwishiensis | Orthochromis kalungwishiensis | oreochromini | other | 1 | 1 | 1 | 3.83 | 100 | 3 |
| Orth_kasu | Orthochromis kasuluensis | Orthochromis kasuluensis | oreochromini | other | 1 | 1 | 1 | 3.97 | 100 | 3 |
| Orth_luic | Orthochromis luichensis | Orthochromis luichensis | oreochromini | other | 0 | 1 | 1 | 3.59 | 0 | 0 |
| Orth_mach | Orthochromis machadoi | Orthochromis machadoi | oreochromini | other | 1 | 1 | 1 | 3.54 | 100 | 3 |
| Orth_mala | Orthochromis malagaraziensis | Orthochromis malagaraziensis | oreochromini | other | 1 | 1 | 1 | 3.64 | 75 | 3 |
| Orth_mazi | Orthochromis mazimeroensis | Orthochromis mazimeroensis | oreochromini | other | 0 | 0 | 0 | 3.32 | 0 | 1 |
| Orth_moso | Orthochromis mosoensis | Orthochromis mosoensis | oreochromini | other | 0 | 0 | 0 | 3.75 | 0 | 0 |
| Orth_poly | Orthochromis polyacanthus | Orthochromis polyacanthus | oreochromini | other | 0 | 1 | 1 | 2.53 | 100 | 3 |
| Orth_rubr | Orthochromis rubrolabialis | Orthochromis rubrolabialis | oreochromini | other | 0 | 0 | 0 | 3.57 | 0 | 0 |
| Orth_rugu | Orthochromis rugufuensis | Orthochromis rugufuensis | oreochromini | other | 0 | 0 | 0 | 3.6 | 0 | 0 |
| Orth_stor | Orthochromis stormsi | Orthochromis stormsi | oreochromini | other | 1 | 1 | 1 | 3.34 | 100 | 3 |
| Orth_torr | Orthochromis torrenticola | Orthochromis torrenticola | oreochromini | other | 0 | 0 | 0 | 3.77 | 0 | 0 |
| Orth_uvin | Orthochromis uvinzae | Orthochromis uvinzae | oreochromini | other | 1 | 1 | 1 | 3.56 | 100 | 3 |
| Otop_broo | Otopharynx brooksi | Otopharynx brooksi | haplochromini | malawi | 1 | 1 | 1 | 3.1 | 71 | 3 |
| Otop_hete | Otopharynx heterodon | Otopharynx heterodon | haplochromini | malawi | 0 | 0 | 0 | 2.99 | 0 | 0 |
| Otop_lith | Otopharynx lithobates | Otopharynx lithobates | haplochromini | malawi | 0 | 1 | 1 | 3.38 | 48 | 2 |
| Otop_spec | Otopharynx speciosus | Otopharynx speciosus | haplochromini | malawi | 0 | 0 | 0 | 2.86 | 0 | 0 |
| Otop_tetr | Otopharynx tetrastigma | Otopharynx tetrastigma | haplochromini | malawi | 0 | 0 | 0 | 2.46 | 0 | 1 |
| Pall_toko | Pallidochromis tokolosh | Pallidochromis tokolosh | haplochromini | malawi | 0 | 0 | 0 | 3.84 | 0 | 0 |
| Para_brie | Paracyprichromis brieni | Paracyprichromis brieni | cyprichromini | tanganyika | 0 | 0 | 0 | 4.44 | 0 | 0 |
| Para_nigr | Paracyprichromis nigripinnis | Paracyprichromis nigripinnis | cyprichromini | tanganyika | 0 | 0 | 0 | 4.48 | 0 | 0 |
| Pelm_buet | Pelmatochromis buettikoferi | Pelmatochromis buettikoferi | pelmatochromini | other | 0 | 0 | 0 | 2.2 | 0 | 0 |
| Pelm_nigr | Pelmatochromis nigrofasciatus | Pelmatochromis nigrofasciatus | pelmatochromini | other | 1 | 1 | 1 | 2.94 | 67 | 3 |
| Pelm_cabr | Pelmatolapia cabrae | Pelmatolapia cabrae | pelmatolapiini | other | 0 | 0 | 0 | 2.27 | 0 | 0 |
| Pelm_mari | Pelmatolapia mariae | Pelmatolapia mariae | pelmatolapiini | other | 0 | 0 | 0 | 2.33 | 0 | 1 |
| Pelv_pulc | Pelvicachromis pulcher | Pelvicachromis pulcher | chromidotilapiini | other | 1 | 1 | 1 | 3.45 | 100 | 3 |
| Peri_ecce | Perissodus eccentricus | Perissodus eccentricus | perissodini | tanganyika | 0 | 0 | 0 | 3.42 | 0 | 0 |
| Peri_micr | Perissodus microlepis | Perissodus microlepis | perissodini | tanganyika | 0 | 1 | 1 | 3.89 | 100 | 3 |
| Petr_ephi | Petrochromis ephippium | Petrochromis ephippium | tropheini | tanganyika | 0 | 0 | 0 | 2.67 | 0 | 0 |
| Petr_famu | Petrochromis famula | Petrochromis famula | tropheini | tanganyika | 0 | 0 | 0 | 2.67 | 0 | 0 |
| Petr_fasc | Petrochromis fasciolatus | Petrochromis fasciolatus | tropheini | tanganyika | 0 | 1 | 1 | 2.5 | 76 | 2 |
| Petr_macr | Petrochromis macrognathus | Petrochromis macrognathus | tropheini | tanganyika | 0 | 0 | 0 | 2.47 | 0 | 0 |
| Petr_orth | Petrochromis orthognathus | Petrochromis orthognathus | tropheini | tanganyika | 0 | 0 | 0 | 3 | 0 | 0 |
| Petr_poly | Petrochromis polyodon | Petrochromis polyodon | tropheini | tanganyika | 0 | 0 | 0 | 2.42 | 0 | 0 |
| Petr_trew | Petrochromis trewavasae | Petrochromis trewavasae | tropheini | tanganyika | 0 | 0 | 0 | 2.39 | 0 | 0 |
| Petr_gena | Petrotilapia genalutea | Petrotilapia genalutea | haplochromini | malawi | 0 | 0 | 0 | 2.79 | 0 | 0 |
| Petr_nigr | Petrotilapia nigra | Petrotilapia nigra | haplochromini | malawi | 1 | 1 | 1 | 2.64 | 65 | 3 |
| Phar_acut | Pharyngochromis acuticeps | Pharyngochromis acuticeps | haplochromini | other | 0 | 0 | 0 | 2.95 | 0 | 0 |
| Plac_john | Placidochromis johnstoni | Placidochromis johnstoni | haplochromini | malawi | 1 | 1 | 1 | 3.09 | 71 | 3 |
| Plac_elec | Placidochromis electra | Placidochromis electra | haplochromini | malawi | 0 | 0 | 0 | 2.62 | 0 | 0 |
| Plac_long | Placidochromis longimanus | Placidochromis longimanus | haplochromini | malawi | 0 | 0 | 0 | 2.65 | 0 | 0 |
| Plac_milo | Placidochromis milomo | Placidochromis milomo | haplochromini | malawi | 0 | 0 | 0 | 2.46 | 0 | 0 |
| Plec_elav | Plecodus elaviae | Plecodus elaviae | perissodini | tanganyika | 0 | 0 | 0 | 3.53 | 0 | 0 |
| Plec_mult | Plecodus multidentatus | Plecodus multidentatus | perissodini | tanganyika | 0 | 0 | 0 | 4.19 | 0 | 0 |
| Plec_para | Plecodus paradoxus | Plecodus paradoxus | perissodini | tanganyika | 0 | 1 | 1 | 4.09 | 81 | 3 |
| Plec_stra | Plecodus straeleni | Plecodus straeleni | perissodini | tanganyika | 0 | 0 | 0 | 2.48 | 0 | 0 |
| Prot_anne | Protomelas annectens | Protomelas annectens | haplochromini | malawi | 0 | 1 | 1 | 3.26 | 100 | 3 |
| Prot_fene | Protomelas fenestratus | Protomelas fenestratus | haplochromini | malawi | 0 | 1 | 1 | 2.97 | 68 | 2 |
| Prot_orna | Protomelas ornatus | Protomelas ornatus | haplochromini | malawi | 1 | 1 | 1 | 2.8 | 100 | 3 |
| Prot_simi | Protomelas similis | Protomelas similis | haplochromini | malawi | 1 | 1 | 1 | 3.27 | 100 | 3 |
| Prot_spil | Protomelas spilonotus | Protomelas spilonotus | haplochromini | malawi | 1 | 1 | 1 | 2.81 | 48 | 2 |
| Prot_spilo | Protomelas spilopterus | Protomelas spilopterus | haplochromini | malawi | 1 | 1 | 1 | 2.81 | 100 | 3 |
| Prot_taen | Protomelas taeniolatus | Protomelas taeniolatus | haplochromini | malawi | 1 | 1 | 1 | 2.98 | 100 | 3 |
| Pseu_mult | Pseudocrenilabrus multicolor | Pseudocrenilabrus multicolor | haplochromini | victoria | 1 | 1 | 1 | 2.7 | 100 | 3 |
| Pseu_nich | Pseudocrenilabrus nicholsi | Pseudocrenilabrus nicholsi | haplochromini | other | 1 | 1 | 1 | 2.66 | 100 | 3 |
| Pseu_phil | Pseudocrenilabrus philander | Pseudocrenilabrus philander | haplochromini | other | 1 | 1 | 1 | 2.81 | 100 | 3 |
| Pseu_baba | Pseudosimochromis babaulti | Pseudosimochromis babaulti | tropheini | tanganyika | 0 | 0 | 0 | 2.75 | 0 | 0 |
| Pseu_curv | Pseudosimochromis curvifrons | Pseudosimochromis curvifrons | tropheini | tanganyika | 0 | 1 | 1 | 2.45 | 100 | 3 |
| Pseu_marg | Pseudosimochromis marginatus | Pseudosimochromis marginatus | tropheini | tanganyika | 0 | 0 | 0 | 2.72 | 0 | 0 |
| Pseu_crab | Pseudotropheus crabro | Pseudotropheus crabro | haplochromini | malawi | 1 | 1 | 1 | 3.18 | 78 | 3 |
| Pseu_elon | Pseudotropheus elongatus | Chindongo elongatus | haplochromini | malawi | 0 | 0 | 0 | 3.65 | 0 | 0 |
| Pseu_flav | Pseudotropheus flavus | Chindongo flavus | haplochromini | malawi | 0 | 0 | 0 | 3.56 | 0 | 0 |
| Pseu_joha | Pseudotropheus johannii | Pseudotropheus johannii | haplochromini | malawi | 1 | 1 | 1 | 3.26 | 100 | 3 |
| Pseu_soco | Pseudotropheus socolofi | Chindongo socolofi | haplochromini | malawi | 0 | 0 | 0 | 3.7 | 0 | 0 |
| Pter_cong | Pterochromis congicus | Pterochromis congicus | pelmatochromini | other | 1 | 1 | 1 | 2.72 | 100 | 3 |
| Pund_nyer | Pundamilia nyererei | Pundamilia nyererei | haplochromini | victoria | 0 | 0 | 0 | 3.17 | 0 | 0 |
| Pund_pund | Pundamilia pundamilia | Pundamilia pundamilia | haplochromini | victoria | 0 | 0 | 0 | 2.9 | 0 | 0 |
| Pung_macl | Pungu maclareni | Pungu maclareni | oreochromini | other | 0 | 1 | 1 | 2.95 | 87 | 3 |
| Rega_call | Reganochromis calliurus | Reganochromis calliurus | limnochromini | tanganyika | 1 | 1 | 1 | 5.12 | 100 | 3 |
| Rham_esox | Rhamphochromis esox | Rhamphochromis esox | haplochromini | malawi | 1 | 1 | 1 | 4.78 | 100 | 3 |
| Rham_long | Rhamphochromis longiceps | Rhamphochromis longiceps | haplochromini | malawi | 1 | 1 | 1 | 3.89 | 81 | 3 |
| Rham_wood | Rhamphochromis woodi | Rhamphochromis woodi | haplochromini | malawi | 0 | 0 | 0 | 3.52 | 0 | 0 |
| Sarg_carl | Sargochromis carlottae | Sargochromis carlottae | haplochromini | other | 0 | 0 | 0 | 2.42 | 0 | 0 |
| Sarg_codr | Sargochromis codringtonii | Sargochromis codringtonii | haplochromini | other | 1 | 1 | 1 | 2.61 | 53 | 2 |
| Sarg_giar | Sargochromis giardi | Sargochromis giardi | haplochromini | other | 0 | 0 | 0 | 2.23 | 0 | 0 |
| Sarg_mell | Sargochromis mellandi | Sargochromis mellandi | haplochromini | other | 1 | 1 | 1 | 2.88 | 69 | 3 |
| Saro_caro | Sarotherodon caroli | Sarotherodon caroli | oreochromini | other | 0 | 0 | 0 | 2.86 | 0 | 0 |
| Saro_caud | Sarotherodon caudomarginatus | Sarotherodon caudomarginatus | oreochromini | other | 1 | 1 | 1 | 2.68 | 71 | 3 |
| Saro_gali | Sarotherodon galilaeus | Sarotherodon galilaeus | oreochromini | other | 0 | 0 | 0 | 2.26 | 0 | 0 |
| Saro_linn | Sarotherodon linnelli | Sarotherodon linnellii | oreochromini | other | 0 | 0 | 0 | 2.69 | 0 | 0 |
| Saro_lohb | Sarotherodon lohbergeri | Sarotherodon lohbergeri | oreochromini | other | 0 | 1 | 1 | 2.85 | 100 | 3 |
| Saro_mela | Sarotherodon melanotheron | Sarotherodon melanotheron | oreochromini | other | 0 | 0 | 0 | 2.19 | 9 | 1 |
| Saro_mvog | Sarotherodon mvogoi | Sarotherodon mvogoi | oreochromini | other | 0 | 0 | 0 | 2.68 | 0 | 0 |
| Saro_nigr | Sarotherodon nigripinnis | Sarotherodon nigripinnis | oreochromini | other | 0 | 0 | 0 | 2.3 | 0 | 0 |
| Saro_occi | Sarotherodon occidentalis | Sarotherodon occidentalis | oreochromini | other | 0 | 0 | 0 | 2.15 | 0 | 0 |
| Saro_stei | Sarotherodon steinbachi | Sarotherodon steinbachi | oreochromini | other | 0 | 0 | 0 | 2.91 | 0 | 0 |
| Schw_neod | Schwetzochromis neodon | Schwetzochromis neodon | haplochromini | other | 0 | 0 | 0 | 3.39 | 100 | 3 |
| Scia_bent | Sciaenochromis benthicola | Sciaenochromis benthicola | haplochromini | malawi | 1 | 1 | 1 | 3.4 | 46 | 2 |
| Scia_frye | Sciaenochromis fryeri | Sciaenochromis fryeri | haplochromini | malawi | 0 | 0 | 0 | 3.48 | 0 | 0 |
| Scia_psam | Sciaenochromis psammophilus | Sciaenochromis psammophilus | haplochromini | malawi | 0 | 0 | 0 | 3.45 | 10 | 1 |
| Serr_altu | Serranochromis altus | Serranochromis altus | haplochromini | other | 0 | 0 | 0 | 2.51 | 0 | 0 |
| Serr_angu | Serranochromis angusticeps | Serranochromis angusticeps | haplochromini | other | 0 | 0 | 0 | 2.96 | 100 | 3 |
| Serr_macr | Serranochromis macrocephalus | Serranochromis macrocephalus | haplochromini | other | 1 | 1 | 1 | 2.97 | 100 | 3 |
| Serr_robu | Serranochromis robustus | Serranochromis robustus | haplochromini | malawi | 1 | 1 | 1 | 2.91 | 100 | 3 |
| Serr_stap | Serranochromis stappersi | Serranochromis stappersi | haplochromini | other | 0 | 0 | 0 | 3.24 | 0 | 0 |
| Serr_thum | Serranochromis thumbergi | Serranochromis thumbergi | haplochromini | other | 1 | 1 | 1 | 3.14 | 100 | 3 |
| Simo_diag | Simochromis diagramma | Simochromis diagramma | tropheini | tanganyika | 0 | 1 | 1 | 2.8 | 100 | 3 |
| Spat_eryt | Spathodus erythrodon | Spathodus erythrodon | eretmodini | tanganyika | 0 | 0 | 0 | 3.22 | 0 | 0 |
| Spat_marl | Spathodus marlieri | Spathodus marlieri | eretmodini | tanganyika | 0 | 0 | 0 | 3.23 | 0 | 0 |
| Stea_bleh | Steatocranus bleheri | Steatocranus bleheri | steatocranini | other | 1 | 1 | 1 | 3.39 | 53 | 2 |
| Stea_casu | Steatocranus casuarius | Steatocranus casuarius | steatocranini | other | 1 | 1 | 1 | 3.37 | 87 | 3 |
| Stea_gibb | Steatocranus gibbiceps | Steatocranus gibbiceps | steatocranini | other | 0 | 0 | 0 | 3.25 | 0 | 0 |
| Stea_glab | Steatocranus glaber | Steatocranus glaber | steatocranini | other | 1 | 1 | 1 | 2.93 | 67 | 3 |
| Stea_irvi | Steatocranus irvinei | Paragobiocichla irvinei | steatocranini | other | 1 | 1 | 1 | 3.19 | 100 | 3 |
| Stea_tina | Steatocranus tinanti | Steatocranus tinanti | steatocranini | other | 1 | 1 | 1 | 4.08 | 100 | 3 |
| Stea_uban | Steatocranus ubanguiensis | Steatocranus ubanguiensis | steatocranini | other | 1 | 1 | 1 | 4.97 | 100 | 3 |
| Stig_gutt | Stigmatochromis guttatus | Stigmatochromis macrorhynchos | haplochromini | malawi | 0 | 0 | 0 | 4.01 | 53 | 4 |
| Stig_mode | Stigmatochromis modestus | Stigmatochromis modestus | haplochromini | malawi | 0 | 0 | 0 | 3.11 | 0 | 0 |
| Stig_wood | Stigmatochromis woodi | Stigmatochromis woodi | haplochromini | malawi | 0 | 0 | 0 | 3.32 | 66 | 4 |
| Stom_mari | Stomatepia mariae | Stomatepia mariae | oreochromini | other | 1 | 1 | 1 | 3.43 | 91 | 3 |
| Stom_mong | Stomatepia mongo | Stomatepia mongo | oreochromini | other | 0 | 0 | 0 | 3.88 | 0 | 0 |
| Stom_pind | Stomatepia pindu | Stomatepia pindu | oreochromini | other | 0 | 1 | 1 | 2.79 | 100 | 3 |
| Taen_holo | Taeniochromis holotaenia | Taeniochromis holotaenia | haplochromini | malawi | 0 | 1 | 1 | 3.19 | 100 | 3 |
| Taen_furc | Taeniolethrinops furcicauda | Taeniolethrinops furcicauda | haplochromini | malawi | 0 | 0 | 0 | 3.16 | 85 | 4 |
| Taen_lati | Taeniolethrinops laticeps | Taeniolethrinops laticeps | haplochromini | malawi | 0 | 0 | 0 | 3.21 | 100 | 4 |
| Taen_prae | Taeniolethrinops praeorbitalis | Taeniolethrinops praeorbitalis | haplochromini | malawi | 0 | 0 | 0 | 2.99 | 0 | 0 |
| Tang_irsa | Tanganicodus irsacae | Tanganicodus irsacae | eretmodini | tanganyika | 0 | 0 | 0 | 3.13 | 0 | 0 |
| Telm_bifr | Telmatochromis bifrenatus | Telmatochromis bifrenatus | lamprologini | tanganyika | 1 | 1 | 1 | 4.2 | 100 | 3 |
| Telm_brac | Telmatochromis brachygnathus | Telmatochromis brachygnathus | lamprologini | tanganyika | 1 | 1 | 1 | 4.08 | 65 | 3 |
| Telm_bric | Telmatochromis brichardi | Telmatochromis brichardi | lamprologini | tanganyika | 1 | 1 | 1 | 4.15 | 100 | 3 |
| Telm_dhon | Telmatochromis dhonti | Telmatochromis dhonti | lamprologini | tanganyika | 0 | 0 | 0 | 3.07 | 0 | 0 |
| Telm_temp | Telmatochromis temporalis | Telmatochromis temporalis | lamprologini | tanganyika | 0 | 0 | 0 | 3.22 | 0 | 0 |
| Telm_vitt | Telmatochromis vittatus | Telmatochromis vittatus | lamprologini | tanganyika | 0 | 1 | 1 | 4.71 | 100 | 3 |
| Thor_albo | Thoracochromis albolabris | Thoracochromis albolabris | haplochromini | other | 0 | 0 | 0 | 3.45 | 0 | 0 |
| Thor_brau | Thoracochromis brauschi | Haplochromis brauschi | haplochromini | other | 1 | 1 | 1 | 2.85 | 78 | 3 |
| Thor_buys | Thoracochromis buysi | Thoracochromis buysi | haplochromini | other | 0 | 0 | 0 | 3.36 | 0 | 0 |
| Thor_call | Thoracochromis callichromus | Haplochromis callichromus | haplochromini | other | 1 | 1 | 1 | 2.86 | 100 | 3 |
| Thor_deme | Thoracochromis demeusii | Haplochromis demeusii | haplochromini | other | 0 | 0 | 0 | 2.63 | 0 | 0 |
| Thor_fasc | Thoracochromis fasciatus | Haplochromis fasciatus | haplochromini | other | 0 | 0 | 0 | 2.56 | 0 | 0 |
| Thor_stig | Thoracochromis stigmatogenys | Haplochromis stigmatogenys | haplochromini | other | 0 | 0 | 0 | 3.02 | 0 | 0 |
| Thor_wing | Thoracochromis wingatii | Thoracochromis wingatii | haplochromini | other | 0 | 1 | 1 | 3.03 | 63 | 2 |
| Thys_anso | Thysochromis ansorgii | Thysochromis ansorgii | chromidotilapiini | other | 1 | 1 | 1 | 2.64 | 73 | 3 |
| Tila_brev | Tilapia brevimanus | Tilapia brevimanus | tilapiini | other | 1 | 1 | 1 | 2.96 | 100 | 3 |
| Tila_busu | Tilapia busumana | Tilapia busumana | tilapiini | other | 0 | 0 | 0 | 2.53 | 0 | 0 |
| Tila_guina | Tilapia guinasana | Tilapia guinasana | tilapiini | other | 1 | 1 | 1 | 2.55 | 86 | 3 |
| Tila_guin | Tilapia guineensis | Coptodon guineensis | tilapiini | other | 0 | 0 | 0 | 2.75 | 0 | 0 |
| Tila_ruwe | Tilapia ruweti | Tilapia ruweti | tilapiini | other | 0 | 0 | 0 | 2.79 | 0 | 0 |
| Tila_spar | Tilapia sparrmanii | Tilapia sparrmanii | tilapiini | malawi | 1 | 1 | 1 | 2.39 | 90 | 3 |
| Tram_brev | Tramitichromis brevis | Tramitichromis brevis | haplochromini | malawi | 0 | 0 | 0 | 2.91 | 100 | 4 |
| Tram_vari | Tramitichromis variabilis | Tramitichromis variabilis | haplochromini | malawi | 0 | 0 | 0 | 2.9 | 0 | 0 |
| Trem_unim | Trematocara unimaculatum | Trematocara unimaculatum | bathybatini | tanganyika | 0 | 0 | 0 | 3.21 | 0 | 0 |
| Trem_bent | Trematochromis benthicola | Trematochromis benthicola | haplochromini | tanganyika | 0 | 0 | 0 | 2.88 | 0 | 0 |
| Trem_plac | Trematocranus placodon | Trematocranus placodon | haplochromini | malawi | 0 | 0 | 0 | 3 | 28 | 1 |
| Trig_otos | Triglachromis otostigma | Triglachromis otostigma | limnochromini | tanganyika | 0 | 0 | 0 | 3.77 | 0 | 0 |
| Tris_simo | Tristramella simonis | Tristramella simonis | oreochromini | other | 0 | 0 | 0 | 2.58 | 0 | 0 |
| Trop_poll | Tropheus polli | Tropheus annectens (synonym) | tropheini | tanganyika | 0 | 0 | 0 | 2.73 | 0 | 0 |
| Trop_trop | Tropheops tropheops | Tropheops tropheops | tropheini | tanganyika | 0 | 0 | 0 | 2.75 | 0 | 0 |
| Trop_anne | Tropheus annectens | Tropheus annectens | tropheini | tanganyika | 0 | 0 | 0 | 2.86 | 0 | 0 |
| Trop_bric | Tropheus brichardi | Tropheus brichardi | tropheini | tanganyika | 0 | 0 | 0 | 2.54 | 0 | 0 |
| Trop_dubo | Tropheus duboisi | Tropheus duboisi | tropheini | tanganyika | 0 | 0 | 0 | 2.47 | 0 | 0 |
| Trop_moor | Tropheus moorii | Tropheus moorii | tropheini | tanganyika | 0 | 0 | 0 | 2.51 | 0 | 0 |
| Tylo_late | Tylochromis lateralis | Tylochromis lateralis | tylochromini | other | 0 | 0 | 0 | 2.54 | 0 | 0 |
| Tylo_poly | Tylochromis polylepis | Tylochromis polylepis | tylochromini | tanganyika | 0 | 0 | 0 | 3.11 | 0 | 0 |
| Tyra_macr | Tyrannochromis macrostoma | Tyrannochromis macrostoma | haplochromini | malawi | 1 | 1 | 1 | 3.52 | 94 | 3 |
| Tyra_macu | Tyrannochromis maculiceps | Tyrannochromis macrostoma (synonym) | haplochromini | malawi | 1 | 1 | 1 | 3.07 | 100 | 3 |
| Tyra_nigr | Tyrannochromis nigriventer | Tyrannochromis nigriventer | haplochromini | malawi | 1 | 1 | 1 | 3.36 | 78 | 3 |
| Vari_moor | Variabilichromis moorii | Variabilichromis moorii | lamprologini | tanganyika | 0 | 0 | 0 | 2.8 | 0 | 0 |
| Xeno_hecq | Xenochromis hecqui | Xenochromis hecqui | perissodini | tanganyika | 0 | 0 | 0 | 3.23 | 0 | 0 |
| Xeno_bath | Xenotilapia bathyphila | Xenotilapia bathyphilus | ectodini | tanganyika | 0 | 0 | 0 | 4.26 | 0 | 0 |
| Xeno_boul | Xenotilapia boulengeri | Xenotilapia boulengeri | ectodini | tanganyika | 0 | 0 | 0 | 3.6 | 0 | 0 |
| Xeno_caud | Xenotilapia caudafasciata | Xenotilapia caudafasciata | ectodini | tanganyika | 0 | 0 | 0 | 3.58 | 0 | 0 |
| Xeno_flav | Xenotilapia flavipinnis | Xenotilapia flavipinnis | ectodini | tanganyika | 0 | 0 | 0 | 4.36 | 0 | 0 |
| Xeno_lept | Xenotilapia leptura | Asprotilapia leptura | ectodini | tanganyika | 0 | 0 | 0 | 4.75 | 0 | 0 |
| Xeno_long | Xenotilapia longispinis | Xenotilapia longispinis | ectodini | tanganyika | 0 | 0 | 0 | 3.61 | 0 | 0 |
| Xeno_mela | Xenotilapia melanogenys | Enantiopus melanogenys | ectodini | tanganyika | 0 | 0 | 0 | 5.13 | 0 | 0 |
| Xeno_ochr | Xenotilapia ochrogenys | Xenotilapia ochrogenys | ectodini | tanganyika | 0 | 1 | 1 | 4.05 | 36 | 2 |
| Xeno_rotu | Xenotilapia rotundiventralis | Microdontochromis rotundiventralis | ectodini | tanganyika | 0 | 0 | 0 | 4.15 | 0 | 0 |
| Xeno_sima | Xenotilapia sima | Xenotilapia sima | ectodini | tanganyika | 1 | 1 | 1 | 3.39 | 91 | 3 |
| Xeno_spil | Xenotilapia spiloptera | Xenotilapia spilopterus | ectodini | tanganyika | 0 | 0 | 0 | 3.57 | 0 | 0 |
| Xeno_tenu | Xenotilapia tenuidentatus | Microdontochromis tenuidentatus | ectodini | tanganyika | 0 | 0 | 0 | 4.28 | 0 | 0 |

**Table S2.** Images were compiled from various online image databases and textbooks listed below.

| **ID** | **Species** | **Photograph credits** |
| --- | --- | --- |
| Alco_alca | Alcolapia alcalica | Fishbase 2017, pisces.at |
| Alti_geof | Alticorpus geoffreyi | Fishbase 2017 |
| Alti_macr | Alticorpus macrocleithrum | Fishbase 2017 |
| Alto_calv | Altolamprologus calvus | this paper |
| Alto_comp | Altolamprologus compressiceps | Fishbase 2017 |
| Aris_chri | Aristochromis christyi | Fishbase 2017, this paper |
| Asta_allu | Astatoreochromis alluaudi | Fishbase 2017 |
| Asta_stra | Astatoreochromis straeleni | Fishbase 2017 |
| Asta_bloy | Astatotilapia bloyeti | Fishbase 2017 |
| Asta_burt | Astatotilapia burtoni | pisces.at |
| Asta_call | Astatotilapia calliptera | Fishbase 2017 |
| Asta_desf | Astatotilapia desfontainii | Fishbase 2017, Alexandra Tyers |
| Asta_flav | Astatotilapia flaviijosephi | Fishbase 2017 |
| Asta_stap | Astatotilapia stappersii | Fishbase 2017, pisces.at |
| Asta_twed | Astatotilapia tweddlei | Fishbase 2017 |
| Aulo_baen | Aulonocara baenschi | Fishbase 2017 |
| Aulo_hans | Aulonocara hansbaenschi | Schraml 2005 |
| Aulo_jaco | Aulonocara jacobfreibergi | Schraml 2005, this paper |
| Aulo_minu | Aulonocara sp. minutus | Schraml 2005 |
| Aulo_yell | Aulonocara sp. yellow | Schraml 2005 |
| Aulo_stev | Aulonocara steveni | Fishbase 2017 |
| Aulo_stua | Aulonocara stuartgranti | Fishbase 2017 |
| Aulo_dewi | Aulonocranus dewindti | Fishbase 2017 |
| Bail_cent | Baileychromis centropomoides | Fishbase 2017 |
| Bath_fasc | Bathybates fasciatus | Fishbase 2017, https://www.francecichlid.com/fiches-cichlidae/picture.php?/389 |
| Bath_fero | Bathybates ferox | Pierre Brichard 1989, https://www.monsterfishkeepers.com/forums/threads/bathybates-ferox.635649/ |
| Bath_grau | Bathybates graueri | Fishbase 2017 |
| Bath_horn | Bathybates hornii | Brichard 1978b, Ad Konings |
| Bath_leo | Bathybates leo | Brichard 1978b, Ad Konings |
| Bath_mino | Bathybates minor | Brichard 1978b, Ad Konings |
| Bath_vitt | Bathybates vittatus | Brichard 1978b, Ad Konings |
| Bent_hori | Benthochromis horii | Takahashi 2008 |
| Bent_mela | Benthochromis melanoides | Takahashi 2008 |
| Bent_tric | Benthochromis tricoti | Fishbase 2017 |
| Boul_micr | Boulengerochromis microlepis | Fishbase 2017 |
| Bucc_hete | Buccochromis heterotaenia | Snoeks 2004 |
| Bucc_lept | Buccochromis lepturus | Fishbase 2017 |
| Bucc_noto | Buccochromis nototaenia | Fishbase 2017, this paper |
| Bucc_rhoa | Buccochromis rhoadesii | Fishbase 2017 |
| Call_macr | Callochromis macrops | Fishbase 2017 |
| Call_pleu | Callochromis pleurospilus | Fishbase 2017 |
| Card_scho | Cardiopharynx schoutedeni | Fishbase 2017 |
| Chal_bric | Chalinochromis brichardi | Fishbase 2017 |
| Chal_pope | Chalinochromis popelini | Fishbase 2017 |
| Cham_caer | Champsochromis caeruleus | Fishbase 2017 |
| Cham_spil | Champsochromis spilorhynchus | Fishbase 2017 |
| Chei_euch | Cheilochromis euchilus | Fishbase 2017, this paper |
| Chet_brev | Chetia brevicauda | Bills & Weyl, 2002 |
| Chet_brevis | Chetia brevis | Fishbase 2017 |
| Chet_flav | Chetia flaviventris | www.inaturalist.org/ |
| Chil_dupo | Chilochromis duponti | Fishbase 2017 |
| Chil_rhoa | Chilotilapia rhoadesii | this paper |
| Chro_gunt | Chromidotilapia guntheri | Fishbase 2017 |
| Coel_joka | Coelotilapia joka | Fishbase 2017 |
| Copa_borl | Copadichromis borleyi | Fishbase 2017, Konings 2008, this paper |
| Copa_jack | Copadichromis jacksoni | Fishbase 2017 |
| Copa_mben | Copadichromis mbenjii | Fishbase 2017 |
| Copa_mlot | Copadichromis mloto | Konings 2008 |
| Copa_quad | Copadichromis quadrimaculatus | Konings 2008 |
| Copa_trim | Copadichromis trimaculatus | Konings 2008 |
| Copa_virg | Copadichromis virginalis | Fishbase 2017, Konings 2008 |
| Copt_coff | Coptodon coffea | Fishbase 2017 |
| Copt_dage | Coptodon dageti | Fishbase 2017 |
| Copt_disc | Coptodon discolor | Fishbase 2017 |
| Copt_louk | Coptodon louka | Fishbase 2017 |
| Copt_nyon | Coptodon nyongana | Fishbase 2017 |
| Copt_rend | Coptodon rendalli | Fishbase 2017 |
| Copt_thol | Coptodon tholloni | Fishbase 2017 |
| Copt_walt | Coptodon walteri | Fishbase 2017 |
| Copt_zill | Coptodon zillii | Fishbase 2017 |
| Cten_hore | Ctenochromis horei | Fishbase 2017 |
| Cten_pect | Ctenochromis pectoralis | Fishbase 2017 |
| Cten_inte | Ctenopharynx intermedius | Konings 2008 |
| Cten_niti | Ctenopharynx nitidus | Fishbase 2017 |
| Cten_pict | Ctenopharynx pictus | Konings 2008 |
| Cunn_long | Cunningtonia longiventralis | Baensch 1998 |
| Cyat_obli | Cyathochromis obliquidens | Konings 2008 |
| Cyat_furc | Cyathopharynx furcifer | Fishbase 2017 |
| Cycl_schw | Cyclopharynx schwetzi | [https://megazoo.at](https://megazoo.at/) |
| Cyno_afra | Cynotilapia afra | Fishbase 2017, this paper |
| Cyno_axel | Cynotilapia axelrodi | Fishbase 2017 |
| Cyph_fron | Cyphotilapia frontosa | Fishbase 2017 |
| Cyph_gibb | Cyphotilapia gibberosa | Fishbase 2017 |
| Cypr_colo | Cyprichromis coloratus | Fishbase 2017 |
| Cypr_lept | Cyprichromis leptosoma | Fishbase 2017 |
| Cypr_micr | Cyprichromis microlepidotus | Fishbase 2017 |
| Cypr_pavo | Cyprichromis pavo | Baensch 1998 |
| Cypr_zona | Cyprichromis zonatus | Fishbase 2017, Takahashi, Hori, Nakaya, 2002 |
| Cyrt_moor | Cyrtocara moorii | Fishbase 2017, this paper |
| Dimi_comp | Dimidiochromis compressiceps | Fishbase 2017, this paper |
| Dimi_dimi | Dimidiochromis dimidiatus | Fishbase 2017 |
| Dimi_kiwi | Dimidiochromis kiwinge | Konings 2008 |
| Dimi_stri | Dimidiochromis strigatus | Fishbase 2017, this paper |
| Dipl_limn | Diplotaxodon limnothrissa | Konings 2008 |
| Dipl_macr | Diplotaxodon macrops | Konings 2008 |
| Dipl_simi | Diplotaxodon sp. similis | Konings 2008 |
| Doci_evel | Docimodus evelynae | Konings 2008 |
| Ecto_desc | Ectodus descampsii | Fishbase 2017 |
| Eret_cyan | Eretmodus cyanostictus | Fishbase 2017 |
| Eret_mark | Eretmodus marksmithi | Fishbase 2017, this paper |
| Etia_ngut | Etia nguti | Fishbase 2017 |
| Foss_rost | Fossorochromis rostratus | Fishbase 2017 |
| Geny_ment | Genyochromis mento | Konings 2008 |
| Gnat_perm | Gnathochromis permaxillaris | Fishbase 2017 |
| Gnat_pfef | Gnathochromis pfefferi | Fishbase 2017 |
| Gobi_ethe | Gobiocichla ethelwynnae | Fishbase 2017, http://aqualifestyle-sud.pro-forum.fr/t540-stock-liste-des-poissons-d-eau-douce-de-jardiland-istres-sur-place |
| Gobi_wond | Gobiocichla wonderi | Fishbase 2017 |
| Gram_lema | Grammatotria lemairii | Fishbase 2017 |
| Gree_bell | Greenwoodochromis bellcrossi | Fishbase 2017 |
| Gree_chri | Greenwoodochromis christyi | Fishbase 2017 |
| Hapl_aene | Haplochromis aeneocolor | Fishbase 2017, this paper |
| Hapl_chil | Haplochromis chilotes | this paper |
| Hapl_dege | Haplochromis degeni | Fishbase 2017, Fischlexikon eu (https://www.fischlexikon.eu/fischlexikon/fische-suchen.php?fisch_id=0000001774) |
| Hapl_eleg | Haplochromis elegans | https://www.cichlidae.com/species.php?id=766&content=profile#content |
| Hapl_fisc | Haplochromis fischeri | Fishbase 2017 |
| Hapl_grac | Haplochromis gracilior | Snoeks et al. 2012 |
| Hapl_insi | Haplochromis insidiae | Snoeks et al. 2012 |
| Hapl_lati | Haplochromis latifasciatus | Fishbase 2017, this paper |
| Hapl_macr | Haplochromis macrops | <http://data.nhm.ac.uk/> |
| Hapl_mela | Haplochromis melanopterus | Fishbase 2017, this paper |
| Hapl_micr | Haplochromis microchrysomelas | Snoeks et al. 2012 |
| Hapl_nubi | Haplochromis nubilus | pisces.at |
| Hapl_obli | Haplochromis obliquidens | Fishbase 2017 |
| Hapl_palu | Haplochromis paludinosus | Fishbase 2017 |
| Hapl_phyt | Haplochromis phytophagus | Fishbase 2017, http://www.african-cichlid.com/Phytophagus.htm |
| Hapl_pyrr | Haplochromis pyrrhocephalus | Fishbase 2017 |
| Hapl_rudo | Haplochromis rudolfianus | Fishbase 2017 |
| Hapl_sauv | Haplochromis sauvagei | this paper |
| Hapl_serr | Haplochromis serranus | this paper |
| Hapl_snoe | Haplochromis snoeksi | Schedel et al. 2010, https://www.aquaportail.com/ |
| Hapl_squa | Haplochromis squamipinnis | Fishbase 2017 |
| Hapl_ther | Haplochromis thereuterion | this paper, pisces.at |
| Hapl_vitt | Haplochromis vittatus | McGee et al. 2015, Snoeks et al. 2012 |
| Hapl_trif | Haplotaxodon trifasciatus | https://www.aquainfo.org/article/haplotaxodon-trifasciatus/ |
| Hemi_sten | Hemibates stenosoma | Fishbase 2018 |
| Hemi_cera | Hemichromis cerasogaster | Fishbase 2017 |
| Hemi_elon | Hemichromis elongatus | Fishbase 2017 |
| Hemi_oxyr | Hemitilapia oxyrhyncha | Fishbase 2017 |
| Hete_mult | Heterochromis multidens | Fishbase 2017 |
| Hete_butt | Heterotilapia buttikoferi | Fishbase 2017 |
| Hete_cess | Heterotilapia cessiana | Fishbase 2017 |
| Inte_looc | Interochromis loocki | Fishbase 2017 |
| Iodo_spre | Iodotropheus sprengerae | Fishbase 2017 |
| Juli_dick | Julidochromis dickfeldi | Fishbase 2017 |
| Juli_marl | Julidochromis marlieri | Fishbase 2017 |
| Juli_orna | Julidochromis ornatus | Fishbase 2017 |
| Juli_rega | Julidochromis regani | Fishbase 2017 |
| Juli_tran | Julidochromis transcriptus | Fishbase 2017 |
| Koni_diku | Konia dikume | Fishbase 2017 |
| Koni_eise | Konia eisentrauti | Fishbase 2017 |
| Labe_fuel | Labeotropheus fuelleborni | Fishbase 2017 |
| Labe_trew | Labeotropheus trewavasae | Fishbase 2017 |
| Labi_caer | Labidochromis caeruleus | Fishbase 2017 |
| Labi_giga | Labidochromis gigas | Konings 2008 |
| Labi_iant | Labidochromis ianthinus | Konings 2008 |
| Labi_mylo | Labidochromis mylodon | Konings 2008 |
| Labi_pall | Labidochromis pallidus | Konings 2008 |
| Labi_vell | Labidochromis vellicans | Konings 2008 |
| Lamp_call | Lamprologus callipterus | Pisces.at |
| Lamp_cong | Lamprologus congoensis | Pisces.at |
| Lamp_kung | Lamprologus kungweensis | Pisces.at |
| Lamp_lapa | Lamprologus laparogramma | Bills 1997 |
| Lamp_lema | Lamprologus lemairii | Pisces.at |
| Lamp_mele | Lamprologus meleagris | Fishbase 2017, this paper |
| Lamp_mocq | Lamprologus mocquardi | https://www.aqualog.de/en/lexikon/lamprologus-mocquardi-2/ |
| Lamp_ocel | Lamprologus ocellatus | Pisces.at |
| Lamp_orna | Lamprologus ornatipinnis | Pisces.at |
| Lamp_sign | Lamprologus signatus | Pisces.at |
| Lamp_spec | Lamprologus speciosus | Fishbase 2017 |
| Lamp_teug | Lamprologus teugelsi | Pisces.at |
| Lamp_tigr | Lamprologus tigripictilis | Pisces.at |
| Lamp_wern | Lamprologus werneri | Schelly 2004 |
| Lepi_atte | Lepidiolamprologus attenuatus | Fishbase 2017 |
| Lepi_boul | Lepidiolamprologus boulengeri | Pisces.at |
| Lepi_cunn | Lepidiolamprologus cunningtoni | Fishbase 2017 |
| Lepi_elon | Lepidiolamprologus elongatus | Pisces.at, Fishbase 2017 |
| Lepi_hecq | Lepidiolamprologus hecqui | Pisces.at |
| Lepi_kend | Lepidiolamprologus kendalli | this paper,Fishbase 2017 |
| Lepi_meel | Lepidiolamprologus meeli | Pisces.at |
| Lepi_nkam | Lepidiolamprologus nkambae | Pisces.at |
| Lepi_prof | Lepidiolamprologus profundicola | Fishbase 2017 |
| Lepi_vari | Lepidiolamprologus variostigma | http://philippe-burnel.fr/Photos/Lepidiolamprologus_variostigma.html |
| Lest_pers | Lestradea perspicax | Fishbase 2017 |
| Lest_stap | Lestradea stappersii | https://www.cichlidae.com/species.php?id=1959 |
| Leth_albu | Lethrinops albus | this paper, Konings 2008 |
| Leth_auri | Lethrinops auritus | Konings 2008 |
| Leth_furc | Lethrinops furcifer | Fishbase 2017 |
| Leth_goss | Lethrinops gossei | Konings 2008 |
| Leth_leth | Lethrinops lethrinus | Konings 2008 |
| Leth_longp | Lethrinops longipinnis | Konings 2008 |
| Leth_marg | Lethrinops marginatus | Fishbase 2017 |
| Leth_oliv | Lethrinops sp. oliveri | Konings 2008 |
| Limn_abee | Limnochromis abeelei | Fishbase 2017 |
| Limn_auri | Limnochromis auritus | Fishbase 2017, pisces.at |
| Limn_dard | Limnochromis dardennii | Pisces.at |
| Limn_stan | Limnochromis staneri | http://philippe-burnel.fr/Photos/Limnochromis_staneri.html |
| Lobo_labi | Lobochilotes labiatus | Fishbase 2017 |
| Mayl_auro | Maylandia aurora | Fishbase 2017 |
| Mayl_bene | Maylandia benetos | Konings 2008 |
| Mayl_call | Maylandia callainos | Fishbase 2017 |
| Mayl_chry | Maylandia chrysomallos | Konings 2008 |
| Mayl_esth | Maylandia estherae | Fishbase 2017 |
| Mayl_gres | Maylandia greshakei | Pisces.at |
| Mayl_hete | Maylandia heteropicta | Fishbase 2017 |
| Mayl_lani | Maylandia lanisticola | Pisces.at |
| Mayl_lomb | Maylandia lombardoi | Fishbase 2017 |
| Mayl_mben | Maylandia mbenjii | Fishbase 2017,http://philippe-burnel.fr/Photos/Maylandia_mbenjii.html |
| Mayl_pulp | Maylandia pulpican | Konings 2008 |
| Mayl_xans | Maylandia xanstomachus | Konings 2008 |
| Mayl_zebr | Maylandia zebra | this paper |
| Mche_euci | Mchenga eucinostomus | Konings 2008 |
| Mela_aura | Melanochromis auratus | this paper |
| Mela_kask | Melanochromis kaskazini | this paper |
| Mela_lori | Melanochromis loriae | https://www.cichlidae.com/species.php?id=909 |
| Mela_mela | Melanochromis melanopterus | Konings 2008 |
| Mela_verm | Melanochromis vermivorus | Fishbase 2017 |
| Myak_myak | Myaka myaka | Fishbase 2017, https://jscutler.wordpress.com/cameroon/volcanic-crater-lakes/lake-barombi-mbo/59-myaka-myaka/ |
| Mylo_anap | Mylochromis anaphyrmus | Fishbase 2017 |
| Mylo_epic | Mylochromis epichorialis | Konings 2008 |
| Mylo_eric | Mylochromis ericotaenia | Konings 2008 |
| Mylo_inco | Mylochromis incola | Konings 2008 |
| Mylo_mola | Mylochromis mola | this paper |
| Nano_pari | Nanochromis parilus | Fishbase 2017 |
| Neol_bifa | Neolamprologus bifasciatus | Fishbase 2017, https://www.cichlid-forum.com/profiles/species.php?id=1600 (Ad Konings) |
| Neol_brev | Neolamprologus brevis | Pisces.at |
| Neol_bric | Neolamprologus brichardi | this paper |
| Neol_bues | Neolamprologus buescheri | this paper |
| Neol_caud | Neolamprologus caudopunctatus | this paper |
| Neol_chri | Neolamprologus christyi | Fishbase 2017 |
| Neol_cyli | Neolamprologus cylindricus | this paper |
| Neol_devo | Neolamprologus devosi | Fishbase 2017, R. Schelly et al. 2003 |
| Neol_falc | Neolamprologus falcicula | Pisces.at |
| Neol_fasc | Neolamprologus fasciatus | this paper |
| Neol_furc | Neolamprologus furcifer | Fishbase 2017, SO Kullander et al. 2014, https://cichlidenschmidt.de, http://www.malawicichlidhomepage.com/ |
| Neol_grac | Neolamprologus gracilis | Fishbase 2017 |
| Neol_heli | Neolamprologus helianthus | Pisces.at |
| Neol_lele | Neolamprologus leleupi | Fishbase 2017 |
| Neol_lelo | Neolamprologus leloupi | Fishbase 2017 |
| Neol_long | Neolamprologus longicaudatus | this paper, Pisces.at |
| Neol_longi | Neolamprologus longior | Pisces.at |
| Neol_maru | Neolamprologus marunguensis | Fishbase 2017 |
| Neol_mode | Neolamprologus modestus | Fishbase 2017 |
| Neol_mond | Neolamprologus mondabu | Fishbase 2017 |
| Neol_mult | Neolamprologus multifasciatus | this paper |
| Neol_must | Neolamprologus mustax | Pisces.at |
| Neol_nige | Neolamprologus niger | Fishbase 2017 |
| Neol_nigr | Neolamprologus nigriventris | Fishbase 2017 |
| Neol_obsc | Neolamprologus obscurus | Fishbase 2017 |
| Neol_oliv | Neolamprologus olivaceous | Fishbase 2017 |
| Neol_pect | Neolamprologus pectoralis | Fishbase 2017 |
| Neol_petr | Neolamprologus petricola | Fishbase 2017 |
| Neol_proc | Neolamprologus prochilus | this paper |
| Neol_pulc | Neolamprologus pulcher | this paper |
| Neol_savo | Neolamprologus savoryi | Pisces.at |
| Neol_sexf | Neolamprologus sexfasciatus | Pisces.at |
| Neol_simi | Neolamprologus similis | this paper, Pisces.at |
| Neol_sple | Neolamprologus splendens | Fishbase 2017 |
| Neol_tetr | Neolamprologus tetracanthus | Pisces.at |
| Neol_timi | Neolamprologus timidus | SO Kullander et al. 2014 |
| Neol_toae | Neolamprologus toae | Fishbase 2017 |
| Neol_tret | Neolamprologus tretocephalus | Pisces.at |
| Neol_vent | Neolamprologus ventralis | http://tanganjika-cichlid.eu/de/gallery/neolamprologus-ventralis-chituta-wf/ |
| Neol_walt | Neolamprologus walteri | Fishbase 2017 |
| Neol_waut | Neolamprologus wauthioni | http://www.israquarium.co.il/Fish/FishIndex/Neolamprologus%20wauthioni.html |
| Nimb_fusc | Nimbochromis fuscotaeniatus | Fishbase 2017 |
| Nimb_linn | Nimbochromis linni | Fishbase 2017 |
| Nimb_livi | Nimbochromis livingstoni | this paper |
| Nimb_poly | Nimbochromis polystigma | Fishbase 2017 |
| Nimb_venu | Nimbochromis venustus | Fishbase 2017 |
| Nyas_pros | Nyassachromis prostoma | this paper |
| Opht_boop | Ophthalmotilapia boops | Fishbase 2017 |
| Opht_hete | Ophthalmotilapia heterodonta | https://www.cichlid-forum.com/profiles/species.php?id=1490 |
| Opht_nasu | Ophthalmotilapia nasuta | Fishbase 2017 |
| Opht_vent | Ophthalmotilapia ventralis | this paper |
| Oreo_amph | Oreochromis amphimelas | Fishbase 2017 |
| Oreo_ande | Oreochromis andersonii | Fishbase 2017 |
| Oreo_aure | Oreochromis aureus | Fishbase 2017 |
| Oreo_chun | Oreochromis chungruruensis | Fishbase 2017 |
| Oreo_escu | Oreochromis esculentus | Fishbase 2017 |
| Oreo_karo | Oreochromis karomo | Fishbase 2017, https://www.destin-tanganyika.com/galerie/picture.php?/2311/categories |
| Oreo_leuc | Oreochromis leucostictus | Fishbase 2017 |
| Oreo_macr | Oreochromis macrochir | Fishbase 2017 |
| Oreo_moss | Oreochromis mossambicus | Fishbase 2017 |
| Oreo_mwer | Oreochromis mweruensis | Fishbase 2017 |
| Oreo_nilo | Oreochromis niloticus | Fishbase 2017 |
| Oreo_schw | Oreochromis schwebischi | Fishbase 2017, pisces.at |
| Oreo_shir | Oreochromis shiranus | Fishbase 2017, pisces.at |
| Oreo_squa | Oreochromis squamipinnis | https://malawicichlids.com/mw10002.htm |
| Oreo_tang | Oreochromis tanganicae | pisces.at |
| Oreo_urol | Oreochromis urolepis | pisces.at |
| Oreo_vari | Oreochromis variabilis | Fishbase 2017, http://www.fishbiosystem.ru/PERCIFORMES/Cichlidae/Oreochromis_variabilis2.html |
| Orth_kalu | Orthochromis kalungwishiensis | https://cichlidae.com/species.php?id=672 |
| Orth_kasu | Orthochromis kasuluensis | Fishbase 2017 |
| Orth_luic | Orthochromis luichensis | Fishbase 2017 |
| Orth_mach | Orthochromis machadoi | Fishbase 2017 |
| Orth_mala | Orthochromis malagaraziensis | Fishbase 2017, pisces.at |
| Orth_mazi | Orthochromis mazimeroensis | Fishbase 2017 |
| Orth_moso | Orthochromis mosoensis | Fishbase 2017 |
| Orth_poly | Orthochromis polyacanthus | https://www.practicalfishkeeping.co.uk/features/articles/orthochromis-polyacanthus |
| Orth_rubr | Orthochromis rubrolabialis | Fishbase 2017 |
| Orth_rugu | Orthochromis rugufuensis | Fishbase 2017 |
| Orth_stor | Orthochromis stormsi | https://www.cichlid-forum.com/articles/Ortho_stormsi.php |
| Orth_torr | Orthochromis torrenticola | Fishbase 2017 |
| Orth_uvin | Orthochromis uvinzae | Fishbase 2017, pisces.at |
| Otop_broo | Otopharynx brooksi | Konings 2008 |
| Otop_hete | Otopharynx heterodon | Fishbase 2017 |
| Otop_lith | Otopharynx lithobates | this paper, Konings 2008 |
| Otop_spec | Otopharynx speciosus | Fishbase 2017 |
| Otop_tetr | Otopharynx tetrastigma | pisces.at |
| Pall_toko | Pallidochromis tokolosh | Konings 2008 |
| Para_brie | Paracyprichromis brieni | Fishbase 2017 |
| Para_nigr | Paracyprichromis nigripinnis | Fishbase 2017 |
| Pelm_buet | Pelmatochromis buettikoferi | Fishbase 2017 |
| Pelm_nigr | Pelmatochromis nigrofasciatus | Fishbase 2017 |
| Pelm_cabr | Pelmatolapia cabrae | Fishbase 2017 |
| Pelm_mari | Pelmatolapia mariae | Fishbase 2017 |
| Pelv_pulc | Pelvicachromis pulcher | Fishbase 2017 |
| Peri_ecce | Perissodus eccentricus | http://www.cichlids.ru/mcforum/index.php?/gallery/image/23325-perissodus-eccentricus/ |
| Peri_micr | Perissodus microlepis | this paper |
| Petr_ephi | Petrochromis ephippium | Fishbase 2017, pisces.at |
| Petr_famu | Petrochromis famula | Fishbase 2017, pisces.at |
| Petr_fasc | Petrochromis fasciolatus | Fishbase 2017, pisces.at |
| Petr_macr | Petrochromis macrognathus | https://www.aquainfo.org/article/petrochromis-macrognathus/ |
| Petr_orth | Petrochromis orthognathus | http://www.allfishes.net/photos%20poissons/photos%20tanga/Petrochromis%20orthognathus%20lkola.html |
| Petr_poly | Petrochromis polyodon | Fishbase 2017 |
| Petr_trew | Petrochromis trewavasae | Fishbase 2017 |
| Petr_gena | Petrotilapia genalutea | Fishbase 2017 |
| Petr_nigr | Petrotilapia nigra | Konings 2008 |
| Phar_acut | Pharyngochromis acuticeps | Fishbase 2017 |
| Plac_john | Placidochromis johnstoni | this paper, Konings 2008 |
| Plac_elec | Placidochromis electra | Fishbase 2017 |
| Plac_long | Placidochromis longimanus | Konings 2008 |
| Plac_milo | Placidochromis milomo | Fishbase 2017 |
| Plec_elav | Plecodus elaviae | https://www.ciklid.org/artregister/art.php?ID=1269 |
| Plec_mult | Plecodus multidentatus | http://www.passioneacquari.it/wp-content/uploads/Plecodus-multidentatus-1.jpg |
| Plec_para | Plecodus paradoxus | <https://www.fishipedia.fr/poissons/plecodus-paradoxus> |
| Plec_stra | Plecodus straeleni | Fishbase 2017 |
| Prot_anne | Protomelas annectens | this paper |
| Prot_fene | Protomelas fenestratus | this paper, Fishbase 2017 |
| Prot_orna | Protomelas ornatus | Fishbase 2017 |
| Prot_simi | Protomelas similis | [https://www.malawi-germany.de](https://www.malawi-germany.de/) |
| Prot_spil | Protomelas spilonotus | Fishbase 2017 |
| Prot_spilo | Protomelas spilopterus | Michael K. Oliver 2012 |
| Prot_taen | Protomelas taeniolatus | this paper, Fishbase 2017 |
| Pseu_mult | Pseudocrenilabrus multicolor | this paper |
| Pseu_nich | Pseudocrenilabrus nicholsi | Fishbase 2017 |
| Pseu_phil | Pseudocrenilabrus philander | Fishbase 2017 |
| Pseu_baba | Pseudosimochromis babaulti | Fishbase 2017, pisces.at |
| Pseu_curv | Pseudosimochromis curvifrons | pisces.at |
| Pseu_marg | Pseudosimochromis marginatus | Van Steenberge et al. 2015 |
| Pseu_crab | Pseudotropheus crabro | Fishbase 2017, https://my-fish.org/fishothek/zierfische/p/pseudotropheus-crabro/ |
| Pseu_elon | Pseudotropheus elongatus | Fishbase 2017 |
| Pseu_flav | Pseudotropheus flavus | this paper, Fishbase 2017 |
| Pseu_joha | Pseudotropheus johannii | this paper, Fishbase 2017 |
| Pseu_soco | Pseudotropheus socolofi | Fishbase 2017 |
| Pter_cong | Pterochromis congicus | https://akwa-mania.mud.pl/ryby-i-rosliny/atlas-ryb/ryby-p-2/pterochromis-congicus/, pisces.at |
| Pund_nyer | Pundamilia nyererei | this paper |
| Pund_pund | Pundamilia pundamilia | this paper |
| Pung_macl | Pungu maclareni | https://www.iucnredlist.org/species/18879/8653955, Fishbase 2017 |
| Rega_call | Reganochromis calliurus | Fishbase 2017, https://www.seriouslyfish.com/species/reganochromis-calliurus/ |
| Rham_esox | Rhamphochromis esox | Fishbase 2017, this paper |
| Rham_long | Rhamphochromis longiceps | Konings 2008 |
| Rham_wood | Rhamphochromis woodi | Konings 2008 |
| Sarg_carl | Sargochromis carlottae | https://www.fischlexikon.eu/fischlexikon/fische-suchen.php?fisch_id=0000001488 |
| Sarg_codr | Sargochromis codringtonii | Fishbase 2017 |
| Sarg_giar | Sargochromis giardi | https://de.wikipedia.org/wiki/Sargochromis_giardi#/media/File:Sargochromis_giardi.jpg |
| Sarg_mell | Sargochromis mellandi | https://bangweulufish.wordpress.com/fish/ |
| Saro_caro | Sarotherodon caroli | http://m.transshipet.webnode.com/products/sarotherodon-caroli/ |
| Saro_caud | Sarotherodon caudomarginatus | Fishbase 2017 |
| Saro_gali | Sarotherodon galilaeus | Fishbase 2017 |
| Saro_linn | Sarotherodon linnelli | Fishbase 2017 |
| Saro_lohb | Sarotherodon lohbergeri | Fishbase 2017 |
| Saro_mela | Sarotherodon melanotheron | Fishbase 2017 |
| Saro_mvog | Sarotherodon mvogoi | Fishbase 2017 |
| Saro_nigr | Sarotherodon nigripinnis | Fishbase 2017 |
| Saro_occi | Sarotherodon occidentalis | Fishbase 2017 |
| Saro_stei | Sarotherodon steinbachi | Fishbase 2017 |
| Schw_neod | Schwetzochromis neodon | Fishbase 2017 |
| Scia_bent | Sciaenochromis benthicola | Konings 2008 |
| Scia_frye | Sciaenochromis fryeri | this paper |
| Scia_psam | Sciaenochromis psammophilus | Konings 2008 |
| Serr_altu | Serranochromis altus | Fishbase 2017 |
| Serr_angu | Serranochromis angusticeps | http://v3.boldsystems.org/index.php/Taxbrowser_Taxonpage?taxid=256233 |
| Serr_macr | Serranochromis macrocephalus | Fishbase 2017 |
| Serr_robu | Serranochromis robustus | https://de.wikipedia.org/wiki/Serranochromis_robustus#/media/File:Serranochromis_robustus.jpg |
| Serr_stap | Serranochromis stappersi | https://www.cichlidae.com/species.php?id=1318 |
| Serr_thum | Serranochromis thumbergi | https://bangweulufish.wordpress.com/2011/03/31/first-fish-collection/ |
| Simo_diag | Simochromis diagramma | pisces.at |
| Spat_eryt | Spathodus erythrodon | http://animal-world.com/encyclo/fresh/cichlid/BlueGobyCichlid.php |
| Spat_marl | Spathodus marlieri | Fishbase 2017 |
| Stea_bleh | Steatocranus bleheri | https://www.cichlid-forum.com/articles/steato_bleheri_pt2.php |
| Stea_casu | Steatocranus casuarius | Pisces.at |
| Stea_gibb | Steatocranus gibbiceps | Pisces.at |
| Stea_glab | Steatocranus glaber | Pisces.at |
| Stea_irvi | Steatocranus irvinei | Fishbase 2017, Pisces.at |
| Stea_tina | Steatocranus tinanti | Fishbase 2017, Pisces.at |
| Stea_uban | Steatocranus ubanguiensis | https://www.cichlid-forum.com/profiles/species.php?id=1261 |
| Stig_gutt | Stigmatochromis guttatus | Konings 2008 |
| Stig_mode | Stigmatochromis modestus | Fishbase 2017 |
| Stig_wood | Stigmatochromis woodi | Fishbase 2017 |
| Stom_mari | Stomatepia mariae | Fishbase 2017 |
| Stom_mong | Stomatepia mongo | Fishbase 2017 |
| Stom_pind | Stomatepia pindu | Fishbase 2017 |
| Taen_holo | Taeniochromis holotaenia | Fishbase 2017 |
| Taen_furc | Taeniolethrinops furcicauda | Pisces.at |
| Taen_lati | Taeniolethrinops laticeps | Konings 2008 |
| Taen_prae | Taeniolethrinops praeorbitalis | this paper |
| Tang_irsa | Tanganicodus irsacae | this paper |
| Telm_bifr | Telmatochromis bifrenatus | https://www.destin-tanganyika.com/galerie/picture.php?/1877/tags/222-telmatochromis |
| Telm_brac | Telmatochromis brachygnathus | https://www.destin-tanganyika.com/galerie/picture.php?/1889/category/telmatochromis-brichardi |
| Telm_bric | Telmatochromis brichardi | Fishbase 2017 |
| Telm_dhon | Telmatochromis dhonti | Fishbase 2017 |
| Telm_temp | Telmatochromis temporalis | this paper |
| Telm_vitt | Telmatochromis vittatus | http://v3.boldsystems.org/index.php/Taxbrowser_Taxonpage?taxid=78219 |
| Thor_albo | Thoracochromis albolabris | this paper |
| Thor_brau | Thoracochromis brauschi | Fishbase 2017 |
| Thor_buys | Thoracochromis buysi | Pisces.at, https://www.youtube.com/watch?v=p5GXEFgImts |
| Thor_call | Thoracochromis callichromus | https://www.hippocampus-bildarchiv.de/tier_10561_Thoracochromisdemeusii.htm |
| Thor_deme | Thoracochromis demeusii | Fishbase 2017 |
| Thor_fasc | Thoracochromis fasciatus | <https://data.nhm.ac.uk/> |
| Thor_stig | Thoracochromis stigmatogenys | Boulenger (1902) |
| Thor_wing | Thoracochromis wingatii | Fishbase 2017, Pisces.at |
| Thys_anso | Thysochromis ansorgii | Fishbase 2017 |
| Tila_brev | Tilapia brevimanus | Fishbase 2017 |
| Tila_busu | Tilapia busumana | pisces.at |
| Tila_guina | Tilapia guinasana | Fishbase 2017 |
| Tila_guin | Tilapia guineensis | Fishbase 2017 |
| Tila_ruwe | Tilapia ruweti | pisces.at |
| Tila_spar | Tilapia sparrmanii | pisces.at |
| Tram_brev | Tramitichromis brevis | [http://www.onzemalawicichliden.eu](http://www.onzemalawicichliden.eu/) |
| Tram_vari | Tramitichromis variabilis | https://www.ciklid.org/artregister/art.php?ID=1284 |
| Trem_unim | Trematocara unimaculatum | Fishbase 2017 |
| Trem_bent | Trematochromis benthicola | Konings 2008 |
| Trem_plac | Trematocranus placodon | Fishbase 2017 |
| Trig_otos | Triglachromis otostigma | Fishbase 2017 |
| Tris_simo | Tristramella simonis | pisces.at |
| Trop_poll | Tropheus polli | Fishbase 2017 |
| Trop_trop | Tropheops tropheops | https://www.cichlid-forum.com/profiles/species.php?id=1838 |
| Trop_anne | Tropheus annectens | pisces.at |
| Trop_bric | Tropheus brichardi | pisces.at |
| Trop_dubo | Tropheus duboisi | pisces.at |
| Trop_moor | Tropheus moorii | pisces.at |
| Tylo_late | Tylochromis lateralis | pisces.at |
| Tylo_poly | Tylochromis polylepis | Konings 2008 |
| Tyra_macr | Tyrannochromis macrostoma | https://www.malawi-germany.de/index.php/arten/nonmbuna/tyrannochromis/548-tyrannochromis-maculiceps.html |
| Tyra_macu | Tyrannochromis maculiceps | this paper |
| Tyra_nigr | Tyrannochromis nigriventer | this paper |
| Vari_moor | Variabilichromis moorii | Fishbase 2017 |
| Xeno_hecq | Xenochromis hecqui | pisces.at |
| Xeno_bath | Xenotilapia bathyphila | Fishbase 2017 |
| Xeno_boul | Xenotilapia boulengeri | Fishbase 2017 |
| Xeno_caud | Xenotilapia caudafasciata | Fishbase 2017 |
| Xeno_flav | Xenotilapia flavipinnis | pisces.at |
| Xeno_lept | Xenotilapia leptura | http://www.cichlids.ru/mcforum/index.php?/gallery/image/19265-xenotilapia-longispinis/ |
| Xeno_long | Xenotilapia longispinis | Fishbase 2017 |
| Xeno_mela | Xenotilapia melanogenys | Fishbase 2017 |
| Xeno_ochr | Xenotilapia ochrogenys | pisces.at |
| Xeno_rotu | Xenotilapia rotundiventralis | Fishbase 2017 |
| Xeno_sima | Xenotilapia sima | pisces.at |
| Xeno_spil | Xenotilapia spiloptera | Fishbase 2017 |
| Xeno_tenu | Xenotilapia tenuidentatus | Fishbase 2017 |

**Table S3**. NCBI accession numbers for samples used in phylogenetic reconstruction.

| **Species** | **Abbreviated_name** | **dlx2_genbank** | **lws_genbank** | **mitf_genbank** | **nd2_genbank** | **rag1_genbank** | **rh2_genbank** | **sws2a_genbank** | **sws2b_genbank** | **nd2_plus** |
| --- | --- | --- | --- | --- | --- | --- | --- | --- | --- | --- |
| *abactochromis labrosus* | abac_labr |  |  |  |  |  |  |  |  | NA |
| *alcolapia alcalica* | alco_alca |  |  |  | GQ167781 |  |  |  |  | 0 |
| *alticorpus geoffreyi* | alti_geof |  |  |  |  |  |  |  |  | NA |
| *alticorpus macrocleithrum* | alti_macr |  |  |  |  |  |  |  |  | NA |
| *alticorpus mentale* | alti_ment |  |  |  |  |  |  |  |  | NA |
| *alticorpus peterdaviesi* | alti_pete |  |  |  | AF305287 |  |  |  |  | 0 |
| *alticorpus profundicola* | alti_prof |  |  |  |  |  |  |  |  | NA |
| *altolamprologus calvus* | alto_calv |  |  |  | EF191108 | FJ706499 |  |  |  | 1 |
| *altolamprologus compressiceps* | alto_comp | KP130551 | KP130509 | KP129837 | EF191105 | FJ706500 |  |  |  | 4 |
| *aristochromis christyi* | aris_chri |  |  |  | EF585282 | JQ073279 |  | JF262750 | HQ993472 | 1 |
| *astatoreochromis alluaudi* | asta_allu |  | AB090437 | KM263705 | AY930075 | DQ012217 |  |  |  | 3 |
| *astatoreochromis straeleni* | asta_stra |  |  |  | KJ176275 |  |  |  |  | 0 |
| *astatoreochromis vanderhorsti* | asta_vand |  |  |  |  |  |  |  |  | NA |
| *astatotilapia bloyeti* | asta_bloy |  |  |  | AY930058 | KF557106 |  |  |  | 1 |
| *astatotilapia burtoni* | asta_burt | KC285401 | KP130476 | KM263693 | AY930060 | DQ012245 | NM_001286317 |  |  | 4 |
| *astatotilapia calliptera* | asta_call | GU936498 |  | GU946332 | AY930090 |  |  |  |  | 2 |
| *astatotilapia desfontainii* | asta_desf |  |  |  | JQ950378 |  |  |  |  | 0 |
| *astatotilapia flaviijosephi* | asta_flav |  |  |  | JQ950380 |  |  |  |  | 0 |
| *astatotilapia stappersii* | asta_stap | KP130557 | KP130514 | KP129841 | AY930046 | KP131333 |  |  |  | 4 |
| *astatotilapia swynnertoni* | asta_swyn |  |  |  |  |  |  |  |  | NA |
| *astatotilapia tweddlei* | asta_twed |  |  |  | KJ413180 |  |  |  |  | 0 |
| *aulonocara aquilonium* | aulo_aqui |  |  |  |  |  |  |  |  | NA |
| *aulonocara auditor* | aulo_audi |  |  |  |  |  |  |  |  | NA |
| *aulonocara baenschi* | aulo_baen |  | GQ452104 |  | KJ176269 |  | GQ422499 | GQ422527 | GQ422528 | 1 |
| *aulonocara brevinidus* | aulo_brev |  |  |  |  |  |  |  |  | NA |
| *aulonocara brevirostre* | aulo_brevi |  |  |  |  |  |  |  |  | NA |
| *aulonocara ethelwynnae* | aulo_ethe |  |  |  |  |  |  |  |  | NA |
| *aulonocara gertrudae* | aulo_gert |  |  |  |  |  |  |  |  | NA |
| *aulonocara guentheri* | aulo_guen |  |  |  |  |  |  |  |  | NA |
| *aulonocara hansbaenschi* | aulo_hans |  |  |  | GQ422572 |  |  |  |  | 0 |
| *aulonocara hueseri* | aulo_hues |  | AY780517 |  |  |  | AY775090 | AY775074 | AY775083 | NA |
| *aulonocara jacobfreibergi* | aulo_jaco |  |  | JX193878 |  |  |  |  |  | NA |
| *aulonocara kandeensis* | aulo_kand |  |  |  |  |  |  |  |  | NA |
| *aulonocara koningsi* | aulo_koni |  |  |  |  |  |  |  |  | NA |
| *aulonocara korneliae* | aulo_korn |  |  |  |  |  |  |  |  | NA |
| *aulonocara maylandi* | aulo_mayl |  |  |  |  |  |  |  |  | NA |
| *aulonocara nyassae* | aulo_nyas |  |  |  |  |  |  |  |  | NA |
| *aulonocara rostratum* | aulo_rost |  |  |  |  |  |  |  |  | NA |
| *aulonocara saulosi* | aulo_saul |  |  |  |  |  |  |  |  | NA |
| *aulonocara steveni* | aulo_stev |  |  |  |  |  |  |  |  | NA |
| *aulonocara stonemani* | aulo_ston |  |  |  |  |  |  |  |  | NA |
| *aulonocara stuartgranti* | aulo_stua | GU936499 | AB090443 | GU946333 | EU661720 |  |  |  |  | 3 |
| *aulonocara trematocephalum* | aulo_trem |  |  |  |  |  |  |  |  | NA |
| *aulonocranus dewindti* | aulo_dewi | KP130520 | KP130477 | KM263694 | AY337782 | DQ012240 |  |  |  | 4 |
| *baileychromis centropomoides* | bail_cent |  |  |  | AY682511 |  |  |  |  | 0 |
| *bathybates fasciatus* | bath_fasc |  |  |  | AY663734 | KF557094 |  |  |  | 1 |
| *bathybates ferox* | bath_fero |  |  |  | AY663737 |  |  |  |  | 0 |
| *bathybates graueri* | bath_grau | KC285403 | KP130493 | KP129826 | AY663726 | KP131318 |  |  |  | 4 |
| *bathybates hornii* | bath_horn |  |  |  | AY663735 |  |  |  |  | 0 |
| *bathybates leo* | bath_leo |  |  |  | AY663731 |  |  |  |  | 0 |
| *bathybates minor* | bath_mino |  |  |  | AY663722 |  |  |  |  | 0 |
| *bathybates vittatus* | bath_vitt |  |  |  | AY663728 |  |  |  |  | 0 |
| *benthochromis horii* | bent_hori |  |  |  | KM288915 |  |  |  |  | 0 |
| *benthochromis melanoides* | bent_mela |  |  |  | AY682513 |  |  |  |  | 0 |
| *benthochromis tricoti* | bent_tric |  |  |  | AY682515 | KF557096 |  |  |  | 1 |
| *boulengerochromis microlepis* | boul_micr | KC285404 | KP130490 | KP129823 | AF317229 | DQ012235 |  |  |  | 4 |
| *buccochromis atritaeniatus* | bucc_atri |  |  |  |  |  |  |  |  | NA |
| *buccochromis heterotaenia* | bucc_hete | GU936500 |  | GU946334 | EU661719 |  |  |  |  | 2 |
| *buccochromis lepturus* | bucc_lept |  |  |  | U07241 |  |  |  |  | 0 |
| *buccochromis nototaenia* | bucc_noto |  |  |  |  |  |  |  |  | NA |
| *buccochromis oculatus* | bucc_ocul |  |  |  | AF305300 |  |  |  |  | 0 |
| *buccochromis rhoadesii* | bucc_rhoa |  |  |  |  |  |  |  |  | NA |
| *buccochromis spectabilis* | bucc_spec |  |  |  |  |  |  |  |  | NA |
| *callochromis macrops* | call_macr | KC285399 | KP130478 | KM263695 | AY337795 | KM263620 |  |  |  | 4 |
| *callochromis melanostigma* | call_mela |  |  |  |  |  |  |  |  | NA |
| *callochromis pleurospilus* | call_pleu |  |  |  | AY337771 |  |  |  |  | 0 |
| *caprichromis liemi* | capr_liem |  |  |  |  |  |  |  |  | NA |
| *caprichromis orthognathus* | capr_orth |  |  |  |  |  |  |  |  | NA |
| *cardiopharynx schoutedeni* | card_scho |  |  |  | AY337791 | KF557098 |  |  |  | 1 |
| *chalinochromis brichardi* | chal_bric |  | HM135130 |  | HM623820 | KJ399572 | HM135121 |  |  | 2 |
| *chalinochromis cyanophleps* | chal_cyan |  |  |  |  |  |  |  |  | NA |
| *chalinochromis popelini* | chal_pope |  |  |  | U07244 |  |  |  |  | 0 |
| *champsochromis caeruleus* | cham_caer |  |  |  |  |  |  |  |  | NA |
| *champsochromis spilorhynchus* | cham_spil |  |  |  | U07245 |  |  |  |  | 0 |
| *cheilochromis euchilus* | chei_euch | GU936501 |  | GU94633 | AY930092 |  |  |  |  | 2 |
| *chetia brevicauda* | chet_brev |  |  |  | EU753924 |  |  |  |  | 0 |
| *chetia brevis* | chet_brevi |  |  |  | EU753925 |  |  |  |  | 0 |
| *chetia flaviventris* | chet_flav |  |  |  | EU753927 |  |  |  |  | 0 |
| *chetia gracilis* | chet_grac |  |  |  |  |  |  |  |  | NA |
| *chetia mola* | chet_mola |  |  |  |  | KF557099 |  |  |  | NA |
| *chetia welwitschi* | chet_welw |  |  |  |  |  |  |  |  | NA |
| *chilochromis duponti* | chil_dupo |  |  |  | GQ167776 |  |  |  |  | 0 |
| *chilotilapia rhoadesii* | chil_rhoa |  |  |  | JX122940 | KF557100 |  |  |  | 1 |
| *chromidotilapia guntheri guntheri* | chro_gunt |  |  |  | AF317270 | KF359816 |  |  |  | 1 |
| *copadichromis atripinnis* | copa_atri |  |  |  |  |  |  |  |  | NA |
| *copadichromis azureus* | copa_azur |  |  |  |  |  |  |  |  | NA |
| *copadichromis borleyi* | copa_borl |  |  |  | AF305308 |  | AY775071 | AY775061 | AY775065 | 0 |
| *copadichromis chizumuluensis* | copa_chiz |  |  |  |  |  |  |  |  | NA |
| *copadichromis chrysonotus* | copa_chry |  |  |  |  |  |  |  |  | NA |
| *copadichromis cyaneus* | copa_cyan |  | AY673697 |  |  |  |  |  | AY673717 | NA |
| *copadichromis cyanocephalus* | copa_cyan |  |  |  |  |  |  |  |  | NA |
| *copadichromis diplostigma* | copa_dipl |  |  |  |  |  |  |  |  | NA |
| *copadichromis geertsi* | copa_geer |  |  |  |  |  |  |  |  | NA |
| *copadichromis ilesi* | copa_iles |  |  |  |  |  |  |  |  | NA |
| *copadichromis insularis* | copa_insu |  |  |  |  |  |  |  |  | NA |
| *copadichromis jacksoni* | copa_jack |  |  |  | GQ422593 |  |  |  | JF727647 | 0 |
| *copadichromis likomae* | copa_liko |  |  |  |  |  |  |  |  | NA |
| *copadichromis mbenjii* | copa_mben | GU936504 |  | GU946338 | EF585255 |  |  |  |  | 2 |
| *copadichromis melas* | copa_mela |  |  |  |  |  |  |  |  | NA |
| *copadichromis mloto* | copa_mlot |  |  |  |  |  |  |  |  | NA |
| *copadichromis nkatae* | copa_nkat |  |  |  |  |  |  |  |  | NA |
| *copadichromis parvus* | copa_parv |  |  |  |  |  |  |  |  | NA |
| *copadichromis pleurostigma* | copa_pleu |  |  |  |  |  |  |  |  | NA |
| *copadichromis pleurostigmoides* | copa_pleu |  |  |  |  |  |  |  |  | NA |
| *copadichromis quadrimaculatus* | copa_quad |  |  |  | AF305314 |  |  |  |  | 0 |
| *copadichromis trewavasae* | copa_trew |  |  |  |  |  |  |  |  | NA |
| *copadichromis trimaculatus* | copa_trim |  |  | JX193877 | JX122956 |  |  |  |  | 1 |
| *copadichromis verduyni* | copa_verd |  |  |  |  |  |  |  |  | NA |
| *copadichromis virginalis* | copa_virg |  |  |  | AF305283 |  |  |  |  | 0 |
| *corematodus shiranus* | core_shir |  |  |  |  |  |  |  |  | NA |
| *corematodus taeniatus* | core_taen |  |  |  |  |  |  |  |  | NA |
| *ctenochromis benthicola* | cten_bent | KP130539 | KP130496 | KP129828 | KJ176273 | KP131320 |  |  |  | 4 |
| *ctenochromis horei* | cten_hore | KC285402 | KP130498 | KM263698 | AY930100 | DQ012250 |  |  |  | 4 |
| *ctenochromis luluae* | cten_lulu |  |  |  |  |  |  |  |  | NA |
| *ctenochromis oligacanthus* | cten_olig |  |  |  | AF416779 |  |  |  |  | 0 |
| *ctenochromis pectoralis* | cten_pect |  |  |  | EU753939 |  |  |  |  | 0 |
| *ctenochromis polli* | cten_poll |  |  |  | EU753941 |  |  |  |  | 0 |
| *ctenopharynx intermedius* | cten_inte |  |  |  |  |  |  |  |  | NA |
| *ctenopharynx nitidus* | cten_niti |  |  |  |  |  |  |  |  | NA |
| *ctenopharynx pictus* | cten_pict | GU936525 |  | GU946359 | GQ422587 |  |  |  |  | 2 |
| *cunningtonia longiventralis* | cunn_long |  |  |  | AY682516 | DQ012243 |  |  |  | 1 |
| *cyathochromis obliquidens* | cyat_obli | GU936503 |  | GU946337 | GU946220 |  |  |  |  | 2 |
| *cyathopharynx furcifer* | cyat_furc | KC285400 |  |  | AY337781 | DQ012241 |  |  |  | 2 |
| *cyclopharynx fwae* | cycl_fwae |  |  |  | AY930099 |  |  |  |  | 0 |
| *cyclopharynx schwetzi* | cycl_schw |  |  |  | JX157103 |  |  |  |  | 0 |
| *cynotilapia afra* | cyno_afra | GU936505 | AY780521 | GU946339 | EF585264 |  | AY775094 | AY775079 | AY775088 | 3 |
| *cynotilapia axelrodi* | cyno_axel |  |  |  |  |  |  |  |  | NA |
| *cynotilapia pulpican* | cyno_pulp | KP130560 | KP130517 |  | KJ955414 |  |  |  |  | 2 |
| *cyphotilapia frontosa* | cyph_fron | KC285397 |  | JX135300 | U07247 | DQ012219 |  |  |  | 3 |
| *cyphotilapia gibberosa* | cyph_gibb | KP130545 | KP130502 | KP129832 | KJ176263 | KP131324 |  |  |  | 4 |
| *cyprichromis coloratus* | cypr_colo |  |  |  | AB588096 |  |  |  |  | 0 |
| *cyprichromis leptosoma* | cypr_lept | KP130525 | KP130482 | KP129815 | AY337786 | DQ012234 |  |  |  | 4 |
| *cyprichromis microlepidotus* | cypr_micr |  |  |  | AY740353 |  |  |  |  | 0 |
| *cyprichromis pavo* | cypr_pavo |  |  |  | AY740382 |  |  |  |  | 0 |
| *cyprichromis zonatus* | cypr_zona |  |  |  | AY740377 |  |  |  |  | 0 |
| *cyrtocara moorii* | cyrt_moor | GU936506 |  | GU946340 | AY930089 | KF557102 |  |  |  | 3 |
| *dimidiochromis compressiceps* | dimi_comp | GU936507 | AF247125 | GU946341 | EF585267 | AB915557 | AF247121 |  | HQ993467 | 4 |
| *dimidiochromis dimidiatus* | dimi_dimi |  |  |  |  |  |  |  |  | NA |
| *dimidiochromis kiwinge* | dimi_kiwi | GU936509 |  | GU946343 | AF305322 |  |  |  |  | 2 |
| *dimidiochromis strigatus* | dimi_stri |  |  |  |  |  |  |  |  | NA |
| *diplotaxodon aeneus* | dipl_aene |  |  |  |  |  |  |  |  | NA |
| *diplotaxodon apogon* | dipl_apog |  |  |  |  |  |  |  |  | NA |
| *diplotaxodon argenteus* | dipl_arge |  |  |  |  |  |  |  |  | NA |
| *diplotaxodon ecclesi* | dipl_eccl |  |  |  |  |  |  |  |  | NA |
| *diplotaxodon greenwoodi* | dipl_gree |  |  | DQ239804 | AF305270 |  |  |  |  | 1 |
| *diplotaxodon limnothrissa* | dipl_limn |  |  |  | AF305261 |  |  |  |  | 0 |
| *diplotaxodon macrops* | dipl_macr |  |  |  | AF305268 |  |  |  |  | 0 |
| *docimodus evelynae* | doci_evel | GU936508 |  | GU946342 | EF585252 |  |  |  |  | 2 |
| *docimodus johnstoni* | doci_john |  |  |  |  |  |  |  |  | NA |
| *eclectochromis lobochilus* | ecle_lobo |  |  |  |  |  |  |  |  | NA |
| *eclectochromis ornatus* | ecle_orna | GU936537 |  | GU946371 | EU661717 |  |  |  |  | 2 |
| *ectodus descampsii* | ecto_desc |  |  |  | AY337790 | DQ012232 |  |  |  | 1 |
| *eretmodus cyanostictus* | eret_cyan | KP130537 | KP130494 | KP129827 | EF679244 | DQ012236 |  |  |  | 4 |
| *eretmodus marksmithi* | eret_mark |  |  |  |  |  |  |  |  | NA |
| *etia nguti* | etia_ngut |  |  |  | GQ167777 |  |  |  |  | 0 |
| *exochochromis anagenys* | exoc_anag |  |  |  | AF305315 |  |  |  |  | 0 |
| *fossorochromis rostratus* | foss_rost | GU936510 |  | GU946344 | EF585281 |  |  |  |  | 2 |
| *genyochromis mento* | geny_ment | GU936511 |  | GU946345 | AF305297 |  |  |  |  | 2 |
| *gephyrochromis lawsi* | geph_laws |  |  |  |  |  |  |  |  | NA |
| *gephyrochromis moorii* | geph_moor |  |  |  |  |  |  |  |  | NA |
| *gnathochromis permaxillaris* | gnat_perm | KP130547 | KP130505 | KM263701 | AY682522 | KM263626 |  |  |  | 4 |
| *gnathochromis pfefferi* | gnat_pfef | KP130542 | KP130499 | KP129830 | U07248 | KP131322 |  |  |  | 4 |
| *gobiocichla ethelwynnae* | gobi_ethe |  |  |  | JX910893 | KF557105 |  |  |  | 1 |
| *gobiocichla wonderi* | gobi_wond |  |  |  | GQ167778 |  |  |  |  | 0 |
| *grammatotria lemairii* | gram_lema | KP130522 | KP130479 | KP129813 | AY337787 | DQ012242 |  |  |  | 4 |
| *greenwoodochromis bellcrossi* | gree_bell |  |  |  | AY682524 |  |  |  |  | 0 |
| *greenwoodochromis christyi* | gree_chri |  |  |  | AY682528 |  |  |  |  | 0 |
| *haplochromis acidens* | hapl_acid |  |  |  |  |  |  |  |  | NA |
| *haplochromis adolphifrederici* | hapl_adol |  |  |  |  |  |  |  |  | NA |
| *haplochromis aelocephalus* | hapl_aelo |  |  |  |  |  |  |  |  | NA |
| *haplochromis aeneocolor* | hapl_aene |  | AY673777 |  | JQ950374 |  |  |  |  | 1 |
| *haplochromis akika* | hapl_akik |  |  |  |  |  |  |  |  | NA |
| *haplochromis albertianus* | hapl_albe |  |  |  |  |  |  |  |  | NA |
| *haplochromis altigenis* | hapl_alti |  |  |  |  |  |  |  |  | NA |
| *haplochromis ampullarostratus* | hapl_ampu |  |  |  |  |  |  |  |  | NA |
| *haplochromis angustifrons* | hapl_angu |  | AY673776 |  |  |  |  |  |  | NA |
| *haplochromis annectidens* | hapl_anne |  |  |  |  |  |  |  |  | NA |
| *haplochromis antleter* | hapl_antl |  |  |  |  |  |  |  |  | NA |
| *haplochromis apogonoides* | hapl_apog |  |  |  |  |  |  |  |  | NA |
| *haplochromis arcanus* | hapl_arca |  |  |  |  |  |  |  |  | NA |
| *haplochromis argens* | hapl_arge |  |  |  |  |  |  |  |  | NA |
| *haplochromis argenteus* | hapl_arge |  |  |  |  |  |  |  |  | NA |
| *haplochromis artaxerxes* | hapl_arta |  |  |  |  |  |  |  |  | NA |
| *haplochromis astatodon* | hapl_asta |  |  |  |  |  |  |  |  | NA |
| *haplochromis avium* | hapl_aviu |  |  |  |  |  |  |  |  | NA |
| *haplochromis azureus* | hapl_azur |  |  |  |  |  |  |  |  | NA |
| *haplochromis barbarae* | hapl_barb |  |  |  |  |  |  |  |  | NA |
| *haplochromis bareli* | hapl_bare |  |  |  |  |  |  |  |  | NA |
| *haplochromis bartoni* | hapl_bart |  |  |  |  |  |  |  |  | NA |
| *haplochromis bayoni* | hapl_bayo |  |  |  |  |  |  |  |  | NA |
| *haplochromis beadlei* | hapl_bead |  | AB090398 |  |  |  |  |  |  | NA |
| *haplochromis bicolor* | hapl_bico |  |  |  |  |  |  |  |  | NA |
| *haplochromis boops* | hapl_boop |  |  |  |  |  |  |  |  | NA |
| *haplochromis brownae* | hapl_brow |  |  |  |  |  |  |  |  | NA |
| *haplochromis bullatus* | hapl_bull |  |  |  |  |  |  |  |  | NA |
| *haplochromis bwathondii* | hapl_bwat |  |  |  |  |  |  |  |  | NA |
| *haplochromis cassius* | hapl_cass |  |  |  |  |  |  |  |  | NA |
| *haplochromis cavifrons* | hapl_cavi |  |  |  |  |  |  |  |  | NA |
| *haplochromis chilotes* | hapl_chil |  | AB326169 |  | KJ413182 |  | AY673706 | AY673726 | AY673716 | 1 |
| *haplochromis chlorochrous* | hapl_chlo |  |  |  |  |  |  |  |  | NA |
| *haplochromis chromogynos* | hapl_chro |  |  |  |  |  |  |  |  | NA |
| *haplochromis chrysogynaion* | hapl_chry |  |  |  |  |  |  |  |  | NA |
| *haplochromis cinctus* | hapl_cinc |  |  |  |  |  |  |  |  | NA |
| *haplochromis cinereus* | hapl_cine |  |  |  |  |  |  |  |  | NA |
| *haplochromis cnester* | hapl_cnes |  |  |  |  |  |  |  |  | NA |
| *haplochromis commutabilis* | hapl_comm |  |  |  |  |  |  |  |  | NA |
| *haplochromis coprologus* | hapl_copr |  |  |  |  |  |  |  |  | NA |
| *haplochromis crassilabris* | hapl_cras |  |  |  |  |  |  |  |  | NA |
| *haplochromis crebridens* | hapl_creb |  |  |  |  |  |  |  |  | NA |
| *haplochromis crocopeplus* | hapl_croc |  |  |  |  |  |  |  |  | NA |
| *haplochromis cronus* | hapl_cron |  |  |  |  |  |  |  |  | NA |
| *haplochromis cryptodon* | hapl_cryp |  |  |  |  |  |  |  |  | NA |
| *haplochromis cryptogramma* | hapl_cryp |  |  |  |  |  |  |  |  | NA |
| *haplochromis cyaneus* | hapl_cyan |  |  |  |  |  |  |  |  | NA |
| *haplochromis decticostoma* | hapl_dect |  |  |  |  |  |  |  |  | NA |
| *haplochromis degeni* | hapl_dege |  |  |  | AY930064 |  |  |  |  | 0 |
| *haplochromis dentex* | hapl_dent |  |  |  |  |  |  |  |  | NA |
| *haplochromis dichrourus* | hapl_dich |  |  |  |  |  |  |  |  | NA |
| *haplochromis diplotaenia* | hapl_dipl |  |  |  |  |  |  |  |  | NA |
| *haplochromis dolichorhynchus* | hapl_doli |  |  |  |  |  |  |  |  | NA |
| *haplochromis dolorosus* | hapl_dolo |  |  |  |  |  |  |  |  | NA |
| *haplochromis eduardianus* | hapl_edua |  |  |  |  |  |  |  |  | NA |
| *haplochromis eduardii* | hapl_edua |  |  |  |  |  |  |  |  | NA |
| *haplochromis elegans* | hapl_eleg |  | AY673778 |  | JQ950379 |  |  |  |  | 1 |
| *haplochromis empodisma* | hapl_empo |  |  |  |  |  |  |  |  | NA |
| *haplochromis engystoma* | hapl_engy |  |  |  |  |  |  |  |  | NA |
| *haplochromis erythrocephalus* | hapl_eryt |  |  |  |  |  |  |  |  | NA |
| *haplochromis erythromaculatus* | hapl_eryt |  |  |  |  |  |  |  |  | NA |
| *haplochromis estor* | hapl_esto |  |  |  |  |  |  |  |  | NA |
| *haplochromis eutaenia* | hapl_euta |  |  |  |  |  |  |  |  | NA |
| *haplochromis exspectatus* | hapl_exsp |  |  |  |  |  |  |  |  | NA |
| *haplochromis fischeri* | hapl_fisc |  | AB666986 |  | AY930063 |  |  | AB666651 | AB666718 | 1 |
| *haplochromis flavipinnis* | hapl_flav |  |  |  |  |  |  |  |  | NA |
| *haplochromis flavus* | hapl_flav |  | AY673780 |  |  |  |  |  |  | NA |
| *haplochromis fuelleborni* | hapl_fuel |  |  |  |  |  |  |  |  | NA |
| *haplochromis fuscus* | hapl_fusc |  |  |  |  |  |  |  |  | NA |
| *haplochromis fusiformis* | hapl_fusi |  |  |  |  |  |  |  |  | NA |
| *haplochromis gigas* | hapl_giga |  |  |  |  |  |  |  |  | NA |
| *haplochromis gigliolii* | hapl_gigl |  |  |  |  |  |  |  |  | NA |
| *haplochromis gilberti* | hapl_gilb |  |  |  |  |  |  |  |  | NA |
| *haplochromis goldschmidti* | hapl_gold |  |  |  |  |  |  |  |  | NA |
| *haplochromis gowersii* | hapl_gowe |  |  |  |  |  |  |  |  | NA |
| *haplochromis gracilior* | hapl_grac |  |  |  | AY930079 |  |  |  |  | 0 |
| *haplochromis granti* | hapl_gran |  |  |  |  |  |  |  |  | NA |
| *haplochromis graueri* | hapl_grau |  |  |  |  |  |  |  |  | NA |
| *haplochromis greenwoodi* | hapl_gree |  |  |  |  |  |  |  |  | NA |
| *haplochromis guiarti* | hapl_guia |  |  |  |  |  |  |  |  | NA |
| *haplochromis harpakteridion* | hapl_harp |  |  |  |  |  |  |  |  | NA |
| *haplochromis heusinkveldi* | hapl_heus |  |  |  |  |  |  |  |  | NA |
| *haplochromis hiatus* | hapl_hiat |  | AB448577 |  |  |  |  |  |  | NA |
| *haplochromis howesi* | hapl_howe |  |  |  |  |  |  |  |  | NA |
| *haplochromis humilior* | hapl_humi |  |  |  |  |  |  |  |  | NA |
| *haplochromis humilis* | hapl_humi |  |  |  |  |  |  |  |  | NA |
| *haplochromis igneopinnis* | hapl_igne |  |  |  |  |  |  |  |  | NA |
| *haplochromis insidiae* | hapl_insi |  |  |  | AY930077 |  |  |  |  | 0 |
| *haplochromis iris* | hapl_iris |  | AB448575 |  |  |  |  |  |  | NA |
| *haplochromis ishmaeli* | hapl_ishm |  |  |  |  |  |  |  |  | NA |
| *haplochromis kamiranzovu* | hapl_kami |  |  |  |  |  |  |  |  | NA |
| *haplochromis katavi* | hapl_kata |  | AB090695 |  |  |  |  |  |  | NA |
| *haplochromis katonga* | hapl_kato |  |  |  |  |  |  |  |  | NA |
| *haplochromis katunzii* | hapl_katu |  |  |  |  |  |  |  |  | NA |
| *haplochromis kujunjui* | hapl_kuju |  |  |  |  |  |  |  |  | NA |
| *haplochromis labiatus* | hapl_labi |  |  |  |  |  |  |  |  | NA |
| *haplochromis labriformis* | hapl_labr |  |  |  |  |  |  |  |  | NA |
| *haplochromis lacrimosus* | hapl_lacr |  |  |  |  |  |  |  |  | NA |
| *haplochromis laparogramma* | hapl_lapa |  | AB448561 |  |  |  |  |  |  | NA |
| *haplochromis latifasciatus* | hapl_lati |  |  |  |  |  |  |  |  | NA |
| *haplochromis limax* | hapl_lima |  |  |  |  |  |  |  |  | NA |
| *haplochromis lividus* | hapl_livi |  |  |  |  |  |  |  |  | NA |
| *haplochromis loati* | hapl_loat |  |  |  |  |  |  |  |  | NA |
| *haplochromis longirostris* | hapl_long |  |  |  |  |  |  |  |  | NA |
| *haplochromis luteus* | hapl_lute |  |  |  |  |  |  |  |  | NA |
| *haplochromis macconneli* | hapl_macc |  |  |  |  |  |  |  |  | NA |
| *haplochromis macrocephalus* | hapl_macr |  |  |  |  |  |  |  |  | NA |
| *haplochromis macrognathus* | hapl_macro |  |  |  |  |  |  |  |  | NA |
| *haplochromis macrops* | hapl_macrop |  |  |  |  |  |  |  |  | NA |
| *haplochromis macropsoides* | hapl_macrops |  |  |  | JQ950381 |  |  |  |  | 0 |
| *haplochromis maculipinna* | hapl_macu |  |  |  |  |  |  |  |  | NA |
| *haplochromis mahagiensis* | hapl_maha |  |  |  |  |  |  |  |  | NA |
| *haplochromis maisomei* | hapl_mais |  |  |  |  |  |  |  |  | NA |
| *haplochromis malacophagus* | hapl_mala |  |  |  |  |  |  |  |  | NA |
| *haplochromis mandibularis* | hapl_mand |  |  |  |  |  |  |  |  | NA |
| *haplochromis martini* | hapl_mart |  |  |  |  |  |  |  |  | NA |
| *haplochromis maxillaris* | hapl_maxi |  |  |  |  |  |  |  |  | NA |
| *haplochromis mbipi* | hapl_mbip |  |  |  |  |  |  |  |  | NA |
| *haplochromis megalops* | hapl_mega |  |  |  |  |  |  |  |  | NA |
| *haplochromis melanopterus* | hapl_mela |  |  |  |  |  |  |  |  | NA |
| *haplochromis melanopus* | hapl_mela |  |  |  |  |  |  |  |  | NA |
| *haplochromis melichrous* | hapl_meli |  |  |  |  |  |  |  |  | NA |
| *haplochromis mentatus* | hapl_ment |  |  |  |  |  |  |  |  | NA |
| *haplochromis mento* | hapl_ment |  |  |  |  |  |  |  |  | NA |
| *haplochromis michaeli* | hapl_mich |  |  |  |  |  |  |  |  | NA |
| *haplochromis microchrysomelas* | hapl_micro |  |  |  |  |  |  |  |  | NA |
| *haplochromis microdon* | hapl_microd |  |  |  |  |  |  |  |  | NA |
| *haplochromis multiocellatus* | hapl_mult |  |  |  |  |  |  |  |  | NA |
| *haplochromis mylergates* | hapl_myle |  |  |  |  |  |  |  |  | NA |
| *haplochromis mylodon* | hapl_mylo |  |  |  |  |  |  |  |  | NA |
| *haplochromis nanoserranus* | hapl_nano |  |  |  |  |  |  |  |  | NA |
| *haplochromis nigrescens* | hapl_nigr |  |  |  |  |  |  |  |  | NA |
| *haplochromis nigricans* | hapl_nigr |  |  |  |  |  |  |  |  | NA |
| *haplochromis nigripinnis* | hapl_nigr |  |  |  |  |  |  |  |  | NA |
| *haplochromis nigroides* | hapl_nigr |  |  |  |  |  |  |  |  | NA |
| *haplochromis niloticus* | hapl_nilo |  |  |  |  |  |  |  |  | NA |
| *haplochromis nubilus* | hapl_nubi |  | AB090648 |  | AF305242 |  |  |  |  | 1 |
| *haplochromis nuchisquamulatus* | hapl_nuch |  |  |  |  |  |  |  |  | NA |
| *haplochromis nyanzae* | hapl_nyan |  |  |  |  |  |  |  |  | NA |
| *haplochromis nyererei* | hapl_nyer |  |  |  |  |  |  |  |  | NA |
| *haplochromis obesus* | hapl_obes |  |  |  |  |  |  |  |  | NA |
| *haplochromis obliquidens* | hapl_obli |  |  |  | AY930097 |  |  |  |  | 0 |
| *haplochromis obtusidens* | hapl_obtu |  |  |  |  |  |  |  |  | NA |
| *haplochromis occultidens* | hapl_occu |  |  |  |  |  |  |  |  | NA |
| *haplochromis oligolepis* | hapl_olig |  |  |  |  |  |  |  |  | NA |
| *haplochromis olivaceus* | hapl_oliv |  |  |  |  |  |  |  |  | NA |
| *haplochromis omnicaeruleus* | hapl_omni |  |  |  |  |  |  |  |  | NA |
| *haplochromis oregosoma* | hapl_oreg |  |  |  |  |  |  |  |  | NA |
| *haplochromis orthostoma* | hapl_orth |  |  |  |  |  |  |  |  | NA |
| *haplochromis pachycephalus* | hapl_pach |  |  |  |  |  |  |  |  | NA |
| *haplochromis pallidus* | hapl_pall |  |  |  |  |  |  |  |  | NA |
| *haplochromis paludinosus* | hapl_palu |  |  |  | AY930107 | DQ012233 |  |  |  | 1 |
| *haplochromis pancitrinus* | hapl_panc |  |  |  |  |  |  |  |  | NA |
| *haplochromis pappenheimi* | hapl_papp |  |  |  |  |  |  |  |  | NA |
| *haplochromis paradoxus* | hapl_para |  |  |  |  |  |  |  |  | NA |
| *haplochromis paraguiarti* | hapl_para |  |  |  |  |  |  |  |  | NA |
| *haplochromis paraplagiostoma* | hapl_para |  |  |  |  |  |  |  |  | NA |
| *haplochromis paropius* | hapl_paro |  |  |  |  |  |  |  |  | NA |
| *haplochromis parorthostoma* | hapl_paro |  |  |  |  |  |  |  |  | NA |
| *haplochromis parvidens* | hapl_parv |  |  |  |  |  |  |  |  | NA |
| *haplochromis paucidens* | hapl_pauc |  |  |  |  |  |  |  |  | NA |
| *haplochromis pellegrini* | hapl_pell |  |  |  |  |  |  |  |  | NA |
| *haplochromis percoides* | hapl_perc |  |  |  |  |  |  |  |  | NA |
| *haplochromis perrieri* | hapl_perr |  |  |  |  |  |  |  |  | NA |
| *haplochromis petronius* | hapl_petr |  |  |  |  |  |  |  |  | NA |
| *haplochromis pharyngalis* | hapl_phar |  | AY673784 |  |  |  |  |  |  | NA |
| *haplochromis pharyngomylus* | hapl_phar |  |  |  |  |  |  |  |  | NA |
| *haplochromis phytophagus* | hapl_phyt |  |  |  | EU753940 |  |  |  |  | 0 |
| *haplochromis piceatus* | hapl_pice |  |  |  |  |  |  |  |  | NA |
| *haplochromis pitmani* | hapl_pitm |  |  |  |  |  |  |  |  | NA |
| *haplochromis placodus* | hapl_plac |  |  |  |  |  |  |  |  | NA |
| *haplochromis plagiodon* | hapl_plag |  | AB090547 |  |  |  |  |  |  | NA |
| *haplochromis plagiostoma* | hapl_plag |  |  |  |  |  |  |  |  | NA |
| *haplochromis plutonius* | hapl_plut |  |  |  |  |  |  |  |  | NA |
| *haplochromis prodromus* | hapl_prod |  |  |  |  |  |  |  |  | NA |
| *haplochromis prognathus* | hapl_prog |  |  |  |  |  |  |  |  | NA |
| *haplochromis pseudopellegrini* | hapl_pseu |  |  |  |  |  |  |  |  | NA |
| *haplochromis ptistes* | hapl_ptis |  |  |  |  |  |  |  |  | NA |
| *haplochromis pundamilia* | hapl_pund |  |  |  |  |  |  |  |  | NA |
| *haplochromis pyrrhocephalus* | hapl_pyrr |  | AB666959 |  | AB915420 | AB915558 |  | AB666625 | AB666693 | 2 |
| *haplochromis pyrrhopteryx* | hapl_pyrrh |  |  |  |  |  |  |  |  | NA |
| *haplochromis retrodens* | hapl_retr |  |  |  |  |  |  |  |  | NA |
| *haplochromis riponianus* | hapl_ripo |  | AB667472 |  |  |  |  |  |  | NA |
| *haplochromis rubescens* | hapl_rube |  |  |  |  |  |  |  |  | NA |
| *haplochromis rubripinnis* | hapl_rubr |  |  |  |  |  |  |  |  | NA |
| *haplochromis rudolfianus* | hapl_rudo |  |  |  | EU753942 |  |  |  |  | 0 |
| *haplochromis rufocaudalis* | hapl_rufo |  |  |  |  |  |  |  |  | NA |
| *haplochromis rufus* | hapl_rufu |  |  |  |  |  |  |  |  | NA |
| *haplochromis sauvagei* | hapl_sauv | KP130556 | KP130513 | KP129840 | KJ955418 | KP131332 |  |  |  | 4 |
| *haplochromis saxicola* | hapl_saxi |  |  |  |  |  |  |  |  | NA |
| *haplochromis scheffersi* | hapl_sche |  |  |  |  |  |  |  |  | NA |
| *haplochromis schubotzi* | hapl_schu |  |  |  |  |  |  |  |  | NA |
| *haplochromis schubotziellus* | hapl_schu |  |  |  |  |  |  |  |  | NA |
| *haplochromis serranus* | hapl_serr |  |  |  |  |  |  |  |  | NA |
| *haplochromis serridens* | hapl_serr |  |  |  |  |  |  |  |  | NA |
| *haplochromis simotes* | hapl_simo |  |  |  |  |  |  |  |  | NA |
| *haplochromis simpsoni* | hapl_simp |  | AB090795 |  |  |  |  |  |  | NA |
| *haplochromis smithii* | hapl_smit |  |  |  |  |  |  |  |  | NA |
| *haplochromis snoeksi* | hapl_snoe |  |  |  | JX157100 |  |  |  |  | 0 |
| *haplochromis sp pink anal* | hapl_pink |  |  |  |  |  |  |  |  | NA |
| *haplochromis sp rockkribensis* | hapl_rock |  |  |  |  |  |  |  |  | NA |
| *haplochromis sp short snout scraper* | hapl_shor |  |  |  |  |  |  |  |  | NA |
| *haplochromis sp stone* | hapl_ston |  |  |  |  |  |  |  |  | NA |
| *haplochromis sp unicuspid scraper* | hapl_unic |  |  |  |  |  |  |  |  | NA |
| *haplochromis spekii* | hapl_spek |  |  |  |  |  |  |  |  | NA |
| *haplochromis sphex* | hapl_sphe |  |  |  |  |  |  |  |  | NA |
| *haplochromis squamipinnis* | hapl_squa |  |  |  | AY930083 |  |  |  |  | 0 |
| *haplochromis squamulatus* | hapl_squa |  |  |  |  |  |  |  |  | NA |
| *haplochromis sulphureus* | hapl_sulp |  |  |  |  |  |  |  |  | NA |
| *haplochromis tanaos* | hapl_tana |  |  |  |  |  |  |  |  | NA |
| *haplochromis taurinus* | hapl_taur |  |  |  |  |  |  |  |  | NA |
| *haplochromis teegelaari* | hapl_teeg |  |  |  |  |  |  |  |  | NA |
| *haplochromis teunisrasi* | hapl_teun |  |  |  |  |  |  |  |  | NA |
| *haplochromis theliodon* | hapl_thel |  |  |  |  |  |  |  |  | NA |
| *haplochromis thereuterion* | hapl_ther |  |  |  | JX157085 |  |  |  |  | 0 |
| *haplochromis thuragnathus* | hapl_thur |  |  |  |  |  |  |  |  | NA |
| *haplochromis tridens* | hapl_trid |  |  |  |  |  |  |  |  | NA |
| *haplochromis turkanae* | hapl_turk |  |  |  |  |  |  |  |  | NA |
| *haplochromis tyrianthinus* | hapl_tyri |  |  |  |  |  |  |  |  | NA |
| *haplochromis ushindi* | hapl_ushi |  |  |  |  |  |  |  |  | NA |
| *haplochromis vanheusdeni* | hapl_vanh |  |  |  |  |  |  |  |  | NA |
| *haplochromis vanoijeni* | hapl_vano |  |  |  |  |  |  |  |  | NA |
| *haplochromis velifer* | hapl_veli |  | AB090614 |  |  |  |  |  |  | NA |
| *haplochromis venator* | hapl_vena |  |  |  |  |  |  |  |  | NA |
| *haplochromis vicarius* | hapl_vica |  |  |  |  |  |  |  |  | NA |
| *haplochromis victoriae* | hapl_vict |  |  |  |  |  |  |  |  | NA |
| *haplochromis victorianus* | hapl_vict |  |  |  |  |  |  |  |  | NA |
| *haplochromis vittatus* | hapl_vitt |  |  |  |  |  |  |  |  | NA |
| *haplochromis vonlinnei* | hapl_vonl |  |  |  |  |  |  |  |  | NA |
| *haplochromis welcommei* | hapl_welc |  |  |  |  |  |  |  |  | NA |
| *haplochromis worthingtoni* | hapl_wort |  |  |  |  |  |  |  |  | NA |
| *haplochromis xanthopteryx* | hapl_xant |  |  |  |  |  |  |  |  | NA |
| *haplochromis xenognathus* | hapl_xeno |  | AB326150 |  |  |  |  |  |  | NA |
| *haplochromis xenostoma* | hapl_xeno |  |  |  |  |  |  |  |  | NA |
| *haplotaxodon microlepis* | hapl_micr | KP130548 | KP130506 | KP129834 | AY682529 | KP131326 |  |  |  | 4 |
| *haplotaxodon trifasciatus* | hapl_trif |  |  |  | EF437492 |  |  |  |  | 0 |
| *hemibates stenosoma* | hemi_sten |  |  |  | AY663719 |  |  |  |  | 0 |
| *hemichromis elongatus* | hemi_elon |  |  |  | AY663714 | KF557109 |  |  |  | 1 |
| *hemitaeniochromis brachyrhynchus* | hemi_brac |  |  |  |  |  |  |  |  | NA |
| *hemitaeniochromis urotaenia* | hemi_urot |  |  |  |  |  |  |  |  | NA |
| *hemitilapia oxyrhyncha* | hemi_oxyr |  |  |  | EF585277 |  |  |  |  | 0 |
| *herichthys cyanoguttatus* | heri_cyan |  |  |  | GU946250 |  |  |  |  | 0 |
| *heterochromis multidens* | hete_mult |  |  |  | AF317269 | JX189868 |  |  |  | 1 |
| *interochromis loocki* | inte_looc |  |  |  | JF900322 |  |  |  |  | 0 |
| *iodotropheus declivitas* | iodo_decl |  |  |  |  |  |  |  |  | NA |
| *iodotropheus sprengerae* | iodo_spre |  |  | JX193875 |  |  |  |  |  | NA |
| *iodotropheus stuartgranti* | iodo_stua |  |  |  |  |  |  |  |  | NA |
| *julidochromis dickfeldi* | juli_dick |  |  |  | HM623790 |  |  |  |  | 0 |
| *julidochromis marlieri* | juli_marl |  |  |  | DQ055039 |  |  |  |  | 0 |
| *julidochromis ornatus* | juli_orna | KP130552 |  | KM263702 | DQ093111 | DQ012237 |  |  |  | 3 |
| *julidochromis regani* | juli_rega |  | HM135132 |  | HM623818 |  | HM135125 |  |  | 1 |
| *julidochromis transcriptus* | juli_tran |  |  |  | HM623792 | AB915552 |  |  |  | 1 |
| *konia dikume* | koni_diku |  |  |  | AJ845105 |  |  |  |  | 0 |
| *konia eisentrauti* | koni_eise |  |  |  | AJ845102 | KF557111 |  |  |  | 1 |
| *labeotropheus fuelleborni* | labe_fuel | GU192280 | AF247127 | GU946346 | GU192139 |  | AF247123 |  |  | 3 |
| *labeotropheus trewavasae* | labe_trew | GU936514 | AB090451 | GU946347 | GQ422578 |  |  |  | JF727646 | 3 |
| *labidochromis caeruleus* | labi_caer | KP130558 | HM049457 | KP129842 | AY740383 | AB915556 | HM049421 | HM049272 | HM049234 | 4 |
| *labidochromis chisumulae* | labi_chis |  | HM049460 |  |  |  | AY775069 | AY775081 | HM049237 | NA |
| *labidochromis flavigulis* | labi_flav |  | HM049461 |  |  |  | HM049386 | HM049276 | HM049238 | NA |
| *labidochromis freibergi* | labi_frei |  |  |  |  |  |  |  |  | NA |
| *labidochromis gigas* | labi_giga | GU936512 | HM049462 | GU946349 | EF585276 |  | HM049407 | HM049278 | HM049240 | 3 |
| *labidochromis heterodon* | labi_hete |  |  |  |  |  |  |  |  | NA |
| *labidochromis ianthinus* | labi_iant |  | HM049463 |  | JX122946 |  | HM049389 | HM049280 | HM049241 | 1 |
| *labidochromis lividus* | labi_livi |  |  |  |  |  |  |  |  | NA |
| *labidochromis maculicauda* | labi_macu |  |  |  |  |  |  |  |  | NA |
| *labidochromis mathotho* | labi_math |  |  |  |  |  |  |  |  | NA |
| *labidochromis mbenjii* | labi_mben |  |  |  |  |  |  |  |  | NA |
| *labidochromis mylodon* | labi_mylo |  |  | JX193862 | JX122959 |  |  |  |  | 1 |
| *labidochromis pallidus* | labi_pall |  |  | JX193880 | JX122935 |  |  |  |  | 1 |
| *labidochromis shiranus* | labi_shir |  |  |  |  |  |  |  |  | NA |
| *labidochromis strigatus* | labi_stri |  |  |  |  |  |  |  |  | NA |
| *labidochromis textilis* | labi_text |  |  |  |  |  |  |  |  | NA |
| *labidochromis vellicans* | labi_vell |  | HM049465 | JX193870 | JX122936 |  | HM049391 | HM049283 | HM049243 | 2 |
| *labidochromis zebroides* | labi_zebr |  |  |  |  |  |  |  |  | NA |
| *lamprologus callipterus* | lamp_call | KP130527 | KP130484 | KP129817 | EF191085 | FJ706526 |  |  |  | 4 |
| *lamprologus congoensis* | lamp_cong |  |  |  | AY740385 | DQ012230 |  |  |  | 1 |
| *lamprologus finalimus* | lamp_fina |  |  |  |  |  |  |  |  | NA |
| *lamprologus kungweensis* | lamp_kung |  |  |  | EF191084 |  |  |  |  | 0 |
| *lamprologus laparogramma* | lamp_lapa |  |  |  | EF191087 |  |  |  |  | 0 |
| *lamprologus lemairii* | lamp_lema |  |  |  | EF191093 | FJ706498 |  |  |  | 1 |
| *lamprologus lethops* | lamp_leth |  |  |  |  |  |  |  |  | NA |
| *lamprologus markerti* | lamp_mark |  |  |  |  |  |  |  |  | NA |
| *lamprologus meleagris* | lamp_mele |  |  |  | EF191098 |  |  |  |  | 0 |
| *lamprologus mocquardi* | lamp_mocq |  |  |  | AF398225 | DQ012226 |  |  |  | 1 |
| *lamprologus ocellatus* | lamp_ocel |  |  |  | EF191115 | FJ706487 |  |  |  | 1 |
| *lamprologus ornatipinnis* | lamp_orna |  |  |  | EF191112 | FJ706538 |  |  |  | 1 |
| *lamprologus signatus* | lamp_sign |  |  |  | EF191086 | FJ706536 |  |  |  | 1 |
| *lamprologus speciosus* | lamp_spec |  |  |  | EF191102 |  |  |  |  | 0 |
| *lamprologus stappersi* | lamp_stap |  |  |  |  |  |  |  |  | NA |
| *lamprologus symoensi* | lamp_symo |  |  |  |  |  |  |  |  | NA |
| *lamprologus teugelsi* | lamp_teug |  |  |  | HM623815 |  |  |  |  | 0 |
| *lamprologus tigripictilis* | lamp_tigr |  |  |  | JX157061 |  |  |  |  | 0 |
| *lamprologus tumbanus* | lamp_tumb |  |  |  |  |  |  |  |  | NA |
| *lamprologus werneri* | lamp_wern |  |  |  | JF961463 |  |  |  |  | 0 |
| *lepidiolamprologus attenuatus* | lepi_atte |  |  |  | AY740387 | FJ706496 |  |  |  | 1 |
| *lepidiolamprologus cunningtoni* | lepi_cunn |  |  |  | HM135113 | FJ706501 |  |  |  | 1 |
| *lepidiolamprologus elongatus* | lepi_elon | KP130528 | KP130485 | KP129818 | EF191092 | FJ706502 |  |  |  | 4 |
| *lepidiolamprologus kamambae* | lepi_kama |  |  |  |  |  |  |  |  | NA |
| *lepidiolamprologus kendalli* | lepi_kend |  |  |  | DQ055043 |  |  |  |  | 0 |
| *lepidiolamprologus mimicus* | lepi_mimi |  |  |  |  |  |  |  |  | NA |
| *lepidiolamprologus profundicola* | lepi_prof |  |  |  | HM623830 | FJ746984 |  |  |  | 1 |
| *lestradea perspicax* | lest_pers |  |  |  | AY337765 |  |  |  |  | 0 |
| *lestradea stappersii* | lest_stap |  |  |  | AY337792 |  |  |  |  | 0 |
| *lethrinops albus* | leth_albu |  |  |  |  |  |  |  |  | NA |
| *lethrinops altus* | leth_altu |  |  |  |  |  |  |  |  | NA |
| *lethrinops argenteus* | leth_arge |  |  |  |  |  |  |  |  | NA |
| *lethrinops auritus* | leth_auri |  |  |  | GQ422586 |  |  |  | HQ993479 | 0 |
| *lethrinops christyi* | leth_chri |  |  |  |  |  |  |  |  | NA |
| *lethrinops furcifer* | leth_furc |  |  |  | AF305317 |  |  |  |  | 0 |
| *lethrinops gossei* | leth_goss |  |  | DQ239803 | AF305291 |  |  |  |  | 1 |
| *lethrinops leptodon* | leth_lept |  |  |  |  |  |  |  |  | NA |
| *lethrinops lethrinus* | leth_leth |  |  |  |  |  |  |  |  | NA |
| *lethrinops longimanus* | leth_long |  |  |  |  |  |  |  |  | NA |
| *lethrinops longipinnis* | leth_long |  |  |  | AF305296 |  |  |  |  | 0 |
| *lethrinops lunaris* | leth_luna |  |  |  |  |  |  |  |  | NA |
| *lethrinops macracanthus* | leth_macr |  |  |  |  |  |  |  |  | NA |
| *lethrinops macrochir* | leth_macr |  |  |  |  |  |  |  |  | NA |
| *lethrinops macrophthalmus* | leth_macr |  |  |  |  |  |  |  |  | NA |
| *lethrinops marginatus* | leth_marg |  |  |  |  |  |  |  |  | NA |
| *lethrinops micrentodon* | leth_micr |  |  |  |  |  |  |  |  | NA |
| *lethrinops microdon* | leth_micr |  |  |  | AF305293 |  |  |  |  | 0 |
| *lethrinops microstoma* | leth_micr |  |  |  |  |  |  |  |  | NA |
| *lethrinops mylodon* | leth_mylo |  |  |  |  |  |  |  |  | NA |
| *lethrinops oculatus* | leth_ocul |  |  |  |  |  |  |  |  | NA |
| *lethrinops parvidens* | leth_parv |  | AY780519 |  |  |  | AY775092 | AY775077 | AY775087 | NA |
| *lethrinops stridei* | leth_stri |  |  |  |  |  |  |  |  | NA |
| *lethrinops turneri* | leth_turn |  |  |  |  |  |  |  |  | NA |
| *lichnochromis acuticeps* | lich_acut |  |  |  |  |  |  |  |  | NA |
| *limnochromis abeelei* | limn_abee |  |  | KM263700 | AY682535 | KM263625 |  |  |  | 2 |
| *limnochromis auritus* | limn_auri |  |  |  | AY682537 | DQ012246 |  |  |  | 1 |
| *limnochromis staneri* | limn_stan |  |  |  | AY682542 |  |  |  |  | 0 |
| *limnotilapia dardennei* | limn_dard | KC285392 |  |  | DQ093109 |  |  |  |  | 1 |
| *lobochilotes labiatus* | lobo_labi | KP130543 | KP130500 | KM263699 | GQ995728 | DQ012210 |  |  |  | 4 |
| *maylandia aurora* | mayl_auro | GU936517 |  | GU946351 | GQ422569 |  |  |  |  | 2 |
| *maylandia barlowi* | mayl_barl |  | HM049468 |  |  |  | HM049394 | HM049286 | HM049248 | NA |
| *maylandia benetos* | mayl_bene |  | HM049471 | JX193866 | JX122961 |  | HM049396 | HM049288 | HM049251 | 2 |
| *maylandia callainos* | mayl_call | GU936516 | HM049474 | AY196319 | GQ422570 |  | HM049409 | HM049291 | HM049254 | 3 |
| *maylandia chrysomallos* | mayl_chry |  |  | JX193864 | JX122954 |  |  |  |  | 1 |
| *maylandia cyneusmarginata* | mayl_cyne |  |  |  |  |  |  |  |  | NA |
| *maylandia elegans* | mayl_eleg |  |  |  |  |  |  |  |  | NA |
| *maylandia emmiltos* | mayl_emmi |  |  |  |  |  |  |  |  | NA |
| *maylandia estherae* | mayl_esth |  |  |  | KJ413177 | JQ073278 |  |  |  | 1 |
| *maylandia flavifemina* | mayl_flav |  |  |  |  |  |  |  |  | NA |
| *maylandia glaucos* | mayl_glau |  |  |  |  |  |  |  |  | NA |
| *maylandia greshakei* | mayl_gres |  |  |  | JX122962 |  |  |  |  | 0 |
| *maylandia hajomaylandi* | mayl_hajo |  |  |  |  |  |  |  |  | NA |
| *maylandia heteropicta* | mayl_hete |  |  |  | GQ422584 |  |  |  |  | 0 |
| *maylandia lanisticola* | mayl_lani |  |  | JX193865 | JX122932 |  |  |  |  | 1 |
| *maylandia livingstonii* | mayl_livi |  |  |  | GQ422582 |  |  |  |  | 0 |
| *maylandia lombardoi* | mayl_lomb |  | HM049480 | JX193872 | JX122944 |  | HM049400 | HM049297 | HM049260 | 2 |
| *maylandia mbenjii* | mayl_mben |  | HM049483 | JX193873 | JX122945 |  | HM049403 | HM049300 | HM049263 | 2 |
| *maylandia melabranchion* | mayl_mela |  |  |  |  |  |  |  |  | NA |
| *maylandia mossambica* | mayl_moss |  |  |  |  |  |  |  |  | NA |
| *maylandia nkhunguensis* | mayl_nkhu |  |  |  |  |  |  |  |  | NA |
| *maylandia phaeos* | mayl_phae |  | HM049486 |  |  |  | HM049406 | HM049303 | HM049266 | NA |
| *maylandia pursa* | mayl_purs |  |  |  |  |  |  |  |  | NA |
| *maylandia pyrsonotos* | mayl_pyrs |  | HM049489 |  |  |  | HM049415 | HM049306 | HM049269 | NA |
| *maylandia sandaracinos* | mayl_sand |  |  |  |  |  |  |  |  | NA |
| *maylandia sciasma* | mayl_scia |  |  |  |  |  |  |  |  | NA |
| *maylandia thapsinogen* | mayl_thap |  |  |  |  |  |  |  |  | NA |
| *maylandia xanstomachus* | mayl_xans |  |  |  | JX122934 |  |  |  |  | 0 |
| *maylandia xanthos* | mayl_xant |  |  |  |  |  |  |  |  | NA |
| *maylandia zebra* | mayl_zebr | GU192357 | AF247126 | AY196318 | GU192222 | DQ012249 | AF247122 |  |  | 4 |
| *mchenga conophoros* | mche_cono |  |  |  |  |  |  |  |  | NA |
| *mchenga cyclicos* | mche_cycl |  |  |  |  |  |  |  |  | NA |
| *mchenga eucinostomus* | mche_euci | GU936502 |  | GU946336 | EF585268 |  |  |  |  | 2 |
| *mchenga flavimanus* | mche_flav |  |  |  |  |  |  |  |  | NA |
| *mchenga inornata* | mche_inor |  |  |  |  |  |  |  |  | NA |
| *mchenga thinos* | mche_thin |  |  |  |  |  |  |  |  | NA |
| *melanochromis auratus* | mela_aura | GU936518 | AY780518 | GU946352 | AY930069 | DQ012220 | AY775091 | AY775076 | AY775084 | 4 |
| *melanochromis baliodigma* | mela_bali |  |  |  |  |  |  |  |  | NA |
| *melanochromis chipokae* | mela_chip |  |  |  |  |  |  |  |  | NA |
| *melanochromis dialeptos* | mela_dial |  |  |  |  |  |  |  |  | NA |
| *melanochromis heterochromis* | mela_hete |  |  |  |  |  |  |  |  | NA |
| *melanochromis kaskazini* | mela_kask |  |  |  |  |  |  |  |  | NA |
| *melanochromis lepidiadaptes* | mela_lepi |  |  |  |  |  |  |  |  | NA |
| *melanochromis loriae* | mela_lori |  | AB090459 | JX193860 | GQ422592 |  |  |  |  | 2 |
| *melanochromis melanopterus* | mela_mela |  |  | JX193876 | JX122957 |  |  |  |  | 1 |
| *melanochromis mossambiquensis* | mela_moss |  |  |  |  |  |  |  |  | NA |
| *melanochromis mpoto* | mela_mpot |  |  |  |  |  |  |  |  | NA |
| *melanochromis robustus* | mela_robu |  |  |  |  |  |  |  |  | NA |
| *melanochromis simulans* | mela_simu |  |  |  |  |  |  |  |  | NA |
| *melanochromis vermivorus* | mela_verm |  | DQ088628 | DQ239797 | EF585270 |  | DQ088631 | DQ088637 | DQ088640 | 2 |
| *melanochromis wochepa* | mela_woch |  |  |  |  |  |  |  |  | NA |
| *myaka myaka* | myak_myak |  |  |  | AJ845107 | KF557115 |  |  |  | 1 |
| *mylochromis anaphyrmus* | mylo_anap |  |  |  | AF305321 |  |  |  |  | 0 |
| *mylochromis balteatus* | mylo_balt |  |  |  |  |  |  |  |  | NA |
| *mylochromis chekopae* | mylo_chek |  |  |  |  |  |  |  |  | NA |
| *mylochromis ensatus* | mylo_ensa |  |  |  |  |  |  |  |  | NA |
| *mylochromis epichorialis* | mylo_epic |  |  | JX193871 | JX122950 |  |  |  |  | 1 |
| *mylochromis ericotaenia* | mylo_eric |  |  |  |  |  |  |  |  | NA |
| *mylochromis formosus* | mylo_form |  |  |  |  |  |  |  |  | NA |
| *mylochromis gracilis* | mylo_grac |  |  |  |  |  |  |  |  | NA |
| *mylochromis guentheri* | mylo_guen |  |  |  |  |  |  |  |  | NA |
| *mylochromis incola* | mylo_inco | GU936519 |  | GU946353 | GU946228 |  |  |  |  | 2 |
| *mylochromis labidodon* | mylo_labi |  |  |  |  |  |  |  |  | NA |
| *mylochromis lateristriga* | mylo_late |  | AY780522 |  |  |  | AY775095 | AY775075 | AY775085 | NA |
| *mylochromis melanonotus* | mylo_mela |  |  |  |  |  |  |  |  | NA |
| *mylochromis melanotaenia* | mylo_mela |  |  |  |  |  |  |  |  | NA |
| *mylochromis mola* | mylo_mola | GU936520 |  | GU946354 | EF585274 |  |  |  |  | 2 |
| *mylochromis mollis* | mylo_moll |  |  |  |  |  |  |  |  | NA |
| *mylochromis obtusus* | mylo_obtu |  |  |  |  |  |  |  |  | NA |
| *mylochromis plagiotaenia* | mylo_plag |  |  |  |  |  |  |  |  | NA |
| *mylochromis semipalatus* | mylo_semi |  |  |  |  |  |  |  |  | NA |
| *mylochromis sphaerodon* | mylo_spha |  |  |  |  |  |  |  |  | NA |
| *mylochromis spilostichus* | mylo_spil |  |  |  |  |  |  |  |  | NA |
| *naevochromis chrysogaster* | naev_chry |  |  |  |  |  |  |  |  | NA |
| *nanochromis parilus* | nano_pari |  |  |  | GQ167814 | KF557116 |  |  |  | 1 |
| *neolamprologus bifasciatus* | neol_bifa |  |  |  | HM623809 |  |  |  |  | 0 |
| *neolamprologus boulengeri* | neol_boul |  |  |  | DQ055040 |  |  |  |  | 0 |
| *neolamprologus brevis* | neol_brev |  |  |  | EF191095 | KF557112 |  |  |  | 1 |
| *neolamprologus brichardi* | neol_bric |  | HM135140 |  | EF679251 |  | AY775068 | AY775072 | AY775062 | 1 |
| *neolamprologus buescheri* | neol_bues |  |  |  | HM623803 |  |  |  |  | 0 |
| *neolamprologus cancellatus* | neol_canc |  |  |  |  |  |  |  |  | NA |
| *neolamprologus caudopunctatus* | neol_caud | KP130526 | KP130483 | KP129816 | AY740388 | KP131308 |  |  |  | 4 |
| *neolamprologus chitamwebwai* | neol_chit |  |  |  |  |  |  |  |  | NA |
| *neolamprologus christyi* | neol_chri |  |  |  | AY740389 |  |  |  |  | 0 |
| *neolamprologus crassus* | neol_cras |  |  |  |  |  |  |  |  | NA |
| *neolamprologus cylindricus* | neol_cyli |  |  |  | DQ093115 | DQ012251 |  |  |  | 1 |
| *neolamprologus devosi* | neol_devo |  |  |  | EF437476 |  |  |  |  | 0 |
| *neolamprologus falcicula* | neol_falc |  |  |  | HM623817 |  |  |  |  | 0 |
| *neolamprologus fasciatus* | neol_fasc | KC285393 |  |  | EF191120 | FJ706492 |  |  |  | 2 |
| *neolamprologus furcifer* | neol_furc | KC285406 | HM135133 |  | HM623812 | KJ399570 |  |  |  | 3 |
| *neolamprologus gracilis* | neol_grac |  |  |  | HM623816 |  |  |  |  | 0 |
| *neolamprologus hecqui* | neol_hecq |  |  |  | DQ055041 |  |  |  |  | 0 |
| *neolamprologus helianthus* | neol_heli |  |  |  | DQ055013 |  |  |  |  | 0 |
| *neolamprologus leleupi* | neol_lele |  |  |  | DQ093113 | DQ012248 |  |  |  | 1 |
| *neolamprologus leloupi* | neol_lelo |  |  |  | EF191104 | FJ706497 |  |  |  | 1 |
| *neolamprologus longicaudatus* | neol_longi |  |  |  | EF462250 |  |  |  |  | 0 |
| *neolamprologus longior* | neol_long |  |  |  | HM623793 |  |  |  |  | 0 |
| *neolamprologus marunguensis* | neol_maru |  |  |  | AY740390 |  |  |  |  | 0 |
| *neolamprologus meeli* | neol_meel |  |  |  | DQ055051 |  |  |  |  | 0 |
| *neolamprologus modestus* | neol_mode | KP130529 | KP130486 | KP129819 | HM623821 | KP131311 |  |  |  | 4 |
| *neolamprologus mondabu* | neol_mond |  | HM135134 |  | EF462242 |  |  |  |  | 1 |
| *neolamprologus multifasciatus* | neol_mult |  |  |  | EF191091 | FJ706506 |  |  |  | 1 |
| *neolamprologus mustax* | neol_must |  |  |  | HM623811 |  |  |  |  | 0 |
| *neolamprologus niger* | neol_nige |  |  |  | AY740391 |  |  |  |  | 0 |
| *neolamprologus nigriventris* | neol_nigr |  |  |  | AY740392 |  |  |  |  | 0 |
| *neolamprologus obscurus* | neol_obsc |  |  |  | HM623824 |  |  |  |  | 0 |
| *neolamprologus olivaceous* | neol_oliv |  |  |  | AY740393 |  |  |  |  | 0 |
| *neolamprologus pectoralis* | neol_pect |  |  |  | EF462238 |  |  |  |  | 0 |
| *neolamprologus petricola* | neol_petr |  |  |  | HM623827 |  |  |  |  | 0 |
| *neolamprologus pleuromaculatus* | neol_pleu |  |  |  |  |  |  |  |  | NA |
| *neolamprologus prochilus* | neol_pruc | KP130540 | KP130497 | KP129829 | HM623825 | KP131321 |  |  |  | 4 |
| *neolamprologus pulcher* | neol_pulc | KP130531 |  | KP129821 | AY740395 | KP131313 |  |  |  | 3 |
| *neolamprologus savoryi* | neol_savo |  |  |  | HM623810 | FJ706539 |  |  |  | 1 |
| *neolamprologus schreyeni* | neol_schr |  |  |  |  |  |  |  |  | NA |
| *neolamprologus sexfasciatus* | neol_sexf |  |  |  | HM623828 |  |  |  |  | 0 |
| *neolamprologus similis* | neol_simi |  |  |  | EF191100 | FJ706477 |  |  |  | 1 |
| *neolamprologus splendens* | neol_sple |  |  |  | HM623799 |  |  |  |  | 0 |
| *neolamprologus tetracanthus* | neol_tetr | KP130530 | KP130487 | KP129820 | HM623822 | KP131312 |  |  |  | 4 |
| *neolamprologus timidus* | neol_timi |  |  |  | KJ187240 | KJ399568 |  |  |  | 1 |
| *neolamprologus toae* | neol_toae |  |  |  | AY682543 | KJ399566 |  |  |  | 1 |
| *neolamprologus tretocephalus* | neol_tret |  | HM135135 |  | EF679253 |  |  |  |  | 1 |
| *neolamprologus variostigma* | neol_vari |  |  |  | DQ055029 |  |  |  |  | 0 |
| *neolamprologus ventralis* | neol_vent |  |  |  | KJ187234 | KJ399562 |  |  |  | 1 |
| *neolamprologus walteri* | neol_walt |  |  |  | HM623808 |  |  |  |  | 0 |
| *neolamprologus wauthioni* | neol_waut |  |  |  | EF191118 |  |  |  |  | 0 |
| *nimbochromis fuscotaeniatus* | nimb_fusc | GU936521 |  | GU946355 | GU946229 |  |  |  |  | 2 |
| *nimbochromis linni* | nimb_linn | GU936522 |  | GU946356 | EF585279 |  |  |  | HQ993475 | 2 |
| *nimbochromis livingstonii* | nimb_livi |  |  | JX193879 | EU753948 |  |  |  |  | 1 |
| *nimbochromis polystigma* | nimb_poly | GU936523 |  | GU946357 | EF585262 |  |  |  |  | 2 |
| *nimbochromis venustus* | nimb_venu |  |  |  | EU753947 | EF033041 |  |  |  | 1 |
| *nyassachromis boadzulu* | nyas_boad |  |  |  |  |  |  |  |  | NA |
| *nyassachromis breviceps* | nyas_brev |  |  |  |  |  |  |  |  | NA |
| *nyassachromis leuciscus* | nyas_leuc |  |  |  |  |  |  |  |  | NA |
| *nyassachromis microcephalus* | nyas_micr |  |  |  |  |  |  |  |  | NA |
| *nyassachromis nigritaeniatus* | nyas_nigr |  |  |  |  |  |  |  |  | NA |
| *nyassachromis prostoma* | nyas_pros | GU936524 |  | GU946358 | EU661715 |  |  |  |  | 2 |
| *nyassachromis purpurans* | nyas_purp |  |  |  |  |  |  |  |  | NA |
| *nyassachromis serenus* | nyas_sere |  |  |  |  |  |  |  |  | NA |
| *ophthalmotilapia boops* | opht_boop |  |  |  | AY337773 |  |  |  |  | 0 |
| *ophthalmotilapia heterodonta* | opht_hete |  |  |  | EF679254 |  |  |  |  | 0 |
| *ophthalmotilapia nasuta* | opht_nasu |  |  |  | AY337783 | DQ012239 |  |  |  | 1 |
| *ophthalmotilapia ventralis* | opht_vent | KP130523 | AY780512 | KM263696 | AY337774 | KF557118 | AY775067 | AY775060 | AY775063 | 4 |
| [*oreochromis amphimelas*](http://fishbase.de/summary/SpeciesSummary.php?id=2035) | oreo_amph |  |  |  | AF317230 |  |  |  |  | 0 |
| [*oreochromis andersonii*](http://fishbase.de/summary/SpeciesSummary.php?id=1399) | oreo_ande |  |  |  | AF317231 |  |  |  |  | 0 |
| [*oreochromis angolensis*](http://fishbase.de/summary/SpeciesSummary.php?id=2036) | oreo_ango |  |  |  |  |  |  |  |  | NA |
| [*oreochromis aureus*](http://fishbase.de/summary/SpeciesSummary.php?id=1387) | oreo_aure | AF534539 |  |  | DQ465029 |  |  |  |  | 1 |
| [*oreochromis chungruruensis*](http://fishbase.de/summary/SpeciesSummary.php?id=2088) | oreo_chun |  |  |  | KF772215 |  |  |  |  | 0 |
| [*oreochromis esculentus*](http://fishbase.de/summary/SpeciesSummary.php?id=1431) | oreo_escu |  |  |  | AF317232 |  |  |  |  | 0 |
| *oreochromis hunteri* | oreo_hunt |  |  |  |  |  |  |  |  | NA |
| [*oreochromis ismailiaensis*](http://fishbase.de/summary/SpeciesSummary.php?id=61388) | oreo_isma |  |  |  |  |  |  |  |  | NA |
| [*oreochromis jipe*](http://fishbase.de/summary/SpeciesSummary.php?id=1422) | oreo_jipe |  |  |  |  |  |  |  |  | NA |
| [*oreochromis karomo*](http://fishbase.de/summary/SpeciesSummary.php?id=1446) | oreo_karo |  |  |  |  |  |  |  |  | NA |
| [*oreochromis karongae*](http://fishbase.de/summary/SpeciesSummary.php?id=2042) | oreo_karo |  |  |  | DQ465030 |  |  |  |  | 0 |
| [*oreochromis korogwe*](http://fishbase.de/summary/SpeciesSummary.php?id=1448) | oreo_koro |  |  |  |  |  |  |  |  | NA |
| [*oreochromis lepidurus*](http://fishbase.de/summary/SpeciesSummary.php?id=2037) | oreo_lepi |  |  |  |  |  |  |  |  | NA |
| [*oreochromis leucostictus*](http://fishbase.de/summary/SpeciesSummary.php?id=1413) | oreo_leuc |  |  |  | AF317233 |  |  |  |  | 0 |
| [*oreochromis lidole*](http://fishbase.de/summary/SpeciesSummary.php?id=2045) | oreo_lido |  |  |  |  |  |  |  |  | NA |
| [*oreochromis macrochir*](http://fishbase.de/summary/SpeciesSummary.php?id=1396) | oreo_macr |  |  |  | AF317235 |  |  |  |  | 0 |
| [*oreochromis mortimeri*](http://fishbase.de/summary/SpeciesSummary.php?id=1415) | oreo_mort |  |  |  |  |  |  |  |  | NA |
| [*oreochromis mossambicus*](http://fishbase.de/summary/SpeciesSummary.php?id=3) | oreo_moss |  |  |  | DQ465032 |  |  |  |  | 0 |
| [*oreochromis mweruensis*](http://fishbase.de/summary/SpeciesSummary.php?id=2502) | oreo_mwer |  |  |  | AF317236 |  |  |  |  | 0 |
| [*oreochromis niloticus*](http://fishbase.de/summary/SpeciesSummary.php?id=2) | oreo_nilo | AF534538 | AF247128 |  | DQ465033 | AB915550 | AF247124 |  |  | 3 |
| [*oreochromis placidus*](http://fishbase.de/summary/SpeciesSummary.php?id=2029) | oreo_plac |  |  |  |  |  |  |  |  | NA |
| [*oreochromis rukwaensis*](http://fishbase.de/summary/SpeciesSummary.php?id=2040) | oreo_rukw |  |  |  |  |  |  |  |  | NA |
| [*oreochromis saka*](http://fishbase.de/summary/SpeciesSummary.php?id=2043) | oreo_saka |  |  |  |  |  |  |  |  | NA |
| [*oreochromis salinicola*](http://fishbase.de/summary/SpeciesSummary.php?id=2041) | oreo_sali |  |  |  |  |  |  |  |  | NA |
| [*oreochromis schwebischi*](http://fishbase.de/summary/SpeciesSummary.php?id=1447) | oreo_schw |  |  |  | AF317238 |  |  |  |  | 0 |
| [*oreochromis shiranus*](http://fishbase.de/summary/SpeciesSummary.php?id=1432) | oreo_shir |  |  |  | KF772216 |  |  |  |  | 0 |
| [*oreochromis spilurus*](http://fishbase.de/summary/SpeciesSummary.php?id=1416) | oreo_spil |  |  |  |  |  |  |  |  | NA |
| [*oreochromis squamipinnis*](http://fishbase.de/summary/SpeciesSummary.php?id=2044) | oreo_squa |  |  |  | KF772214 |  |  |  |  | 0 |
| [*oreochromis tanganicae*](http://fishbase.de/summary/SpeciesSummary.php?id=1440) | oreo_tang | KP130534 | KP130491 | KP129824 | AF317240 | KF557119 |  |  |  | 4 |
| [*oreochromis upembae*](http://fishbase.de/summary/SpeciesSummary.php?id=2038) | oreo_upem |  |  |  |  |  |  |  |  | NA |
| [*oreochromis urolepis*](http://fishbase.de/summary/SpeciesSummary.php?id=1420) | oreo_urol |  |  |  | AF317239 |  |  |  |  | 0 |
| [*oreochromis variabilis*](http://fishbase.de/summary/SpeciesSummary.php?id=1430) | oreo_vari |  |  |  | AF317241 |  |  |  |  | 0 |
| *orthochromis kalungwishiensis* | orth_kalu |  |  |  | KJ176256 |  |  |  |  | 0 |
| *orthochromis kasuluensis* | orth_kasu |  |  |  | AY930049 |  |  |  |  | 0 |
| *orthochromis luichensis* | orth_luic |  |  |  | AY930052 |  |  |  |  | 0 |
| *orthochromis luongoensis* | orth_luon |  |  |  |  | KF557120 |  |  |  | NA |
| *orthochromis machadoi* | orth_mach |  |  |  | EU753936 |  |  |  |  | 0 |
| *orthochromis malagaraziensis* | orth_mala |  |  |  | AY930054 | DQ012229 |  |  |  | 1 |
| *orthochromis mazimeroensis* | orth_mazi |  |  |  | AY930053 |  |  |  |  | 0 |
| *orthochromis mosoensis* | orth_moso |  |  |  | AY930055 |  |  |  |  | 0 |
| *orthochromis polyacanthus* | orth_poly |  |  |  | AF398231 |  |  |  |  | 0 |
| *orthochromis rubrolabialis* | orth_rubr |  |  |  | AY930051 |  |  |  |  | 0 |
| *orthochromis rugufuensis* | orth_rugu |  |  |  | AY930050 |  |  |  |  | 0 |
| *orthochromis stormsi* | orth_stor |  |  |  | AY930057 |  |  |  |  | 0 |
| *orthochromis torrenticola* | orth_torr |  |  |  | JX157111 |  |  |  |  | 0 |
| *orthochromis uvinzae* | orth_uvin |  |  |  | AY930048 | DQ012214 |  |  |  | 1 |
| *otopharynx antron* | otop_antr |  |  |  |  |  |  |  |  | NA |
| *otopharynx argyrosoma* | otop_argy |  |  |  |  |  |  |  |  | NA |
| *otopharynx auromarginatus* | otop_auro |  |  |  |  |  |  |  |  | NA |
| *otopharynx brooksi* | otop_broo |  |  |  | AF305303 |  |  |  |  | 0 |
| *otopharynx decorus* | otop_deco |  |  |  |  |  |  |  |  | NA |
| *otopharynx heterodon* | otop_hete |  |  |  | EF585278 |  |  |  |  | 0 |
| *otopharynx lithobates* | otop_lith | GU936526 |  | GU946360 | EU661716 |  |  |  |  | 2 |
| *otopharynx ovatus* | otop_ovat |  |  |  |  |  |  |  |  | NA |
| *otopharynx pachycheilus* | otop_pach |  |  |  |  |  |  |  |  | NA |
| *otopharynx selenurus* | otop_sele |  |  |  |  |  |  |  |  | NA |
| *otopharynx speciosus* | otop_spec |  |  |  | AF305323 |  |  |  |  | 0 |
| *otopharynx spelaeotes* | otop_spel |  |  |  |  |  |  |  |  | NA |
| *otopharynx tetraspilus* | otop_tetr |  |  |  |  |  |  |  |  | NA |
| *otopharynx tetrastigma* | otop_tetr |  |  |  |  |  |  |  |  | NA |
| *oxylapia polli* | oxyl_poll |  |  |  | AF317275 |  |  |  |  | 0 |
| *pallidochromis tokolosh* | pall_toko |  |  |  | AF305276 |  |  |  |  | 0 |
| *paracyprichromis brieni* | para_brie |  |  |  | AY740378 | KF557121 |  |  |  | 1 |
| *paracyprichromis nigripinnis* | para_nigr |  |  |  | AY740339 |  |  |  |  | 0 |
| *pelmatochromis buettikoferi* | pelm_buet |  |  |  | GQ167783 |  |  |  |  | 0 |
| *pelmatochromis nigrofasciatus* | pelm_nigr |  |  |  | GQ167784 |  |  |  |  | 0 |
| *pelmatochromis ocellifer* | pelm_ocel |  |  |  |  |  |  |  |  | NA |
| *pelvicachromis pulcher* | pelv_pulc |  |  |  | AF317271 |  |  |  |  | 0 |
| *perissodus eccentricus* | peri_ecce |  |  |  | EF437511 |  |  |  |  | 0 |
| *perissodus microlepis* | peri_micr | KP130549 | HM135136 | KP129835 | DQ055006 | DQ012244 |  |  |  | 4 |
| *petrochromis ephippium* | petr_ephi |  |  |  | GQ995736 |  |  |  |  | 0 |
| *petrochromis famula* | petr_famu | KC285405 |  |  | HM135114 |  |  |  |  | 1 |
| *petrochromis fasciolatus* | petr_fasc |  |  |  | GQ995802 |  |  |  |  | 0 |
| *petrochromis horii* | petr_hori |  |  |  |  |  |  |  |  | NA |
| *petrochromis macrognathus* | petr_macr |  |  |  | AY930068 |  |  |  |  | 0 |
| *petrochromis orthognathus* | petr_orth |  |  |  | U07262 |  |  |  |  | 0 |
| *petrochromis polyodon* | petr_poly |  |  |  | GQ995775 | DQ012216 |  |  |  | 1 |
| *petrochromis trewavasae* | petr_trew |  |  |  | GQ995759 |  |  |  |  | 0 |
| *petrotilapia chrysos* | petr_chry |  |  |  |  |  |  |  |  | NA |
| *petrotilapia flaviventris* | petr_flav |  |  |  |  |  |  |  |  | NA |
| *petrotilapia genalutea* | petr_gena |  |  | JX193867 | JX122964 |  |  |  |  | 1 |
| *petrotilapia microgalana* | petr_micr |  |  |  |  |  |  |  |  | NA |
| *petrotilapia mumboensis* | petr_mumb |  |  |  |  |  |  |  |  | NA |
| *petrotilapia nigra* | petr_nigr | GU936536 |  | GU946370 | EU661721 |  |  |  |  | 2 |
| *petrotilapia palingnathos* | petr_pali |  |  |  |  |  |  |  |  | NA |
| *petrotilapia pyroscelos* | petr_pyro |  |  |  |  |  |  |  |  | NA |
| *petrotilapia tridentiger* | petr_trid |  |  |  |  |  |  |  |  | NA |
| *petrotilapia xanthos* | petr_xant |  |  |  |  |  |  |  |  | NA |
| *pharyngochromis acuticeps* | phar_acut |  |  |  | AY930094 |  |  |  |  | 0 |
| *pharyngochromis darlingi* | phar_darl |  |  |  |  |  |  |  |  | NA |
| *placidochromis acuticeps* | plac_acut |  |  |  |  |  |  |  |  | NA |
| *placidochromis acutirostris* | plac_acut |  |  |  |  |  |  |  |  | NA |
| *placidochromis argyrogaster* | plac_argy |  |  |  |  |  |  |  |  | NA |
| *placidochromis boops* | plac_boop |  |  |  |  |  |  |  |  | NA |
| *placidochromis borealis* | plac_bore |  |  |  |  |  |  |  |  | NA |
| *placidochromis chilolae* | plac_chil |  |  |  |  |  |  |  |  | NA |
| *placidochromis communis* | plac_comm |  |  |  |  |  |  |  |  | NA |
| *placidochromis domirae* | plac_domi |  |  |  |  |  |  |  |  | NA |
| *placidochromis ecclesi* | plac_eccl |  |  |  |  |  |  |  |  | NA |
| *placidochromis electra* | plac_elec |  |  |  | JX122952 |  |  |  |  | 0 |
| *placidochromis elongatus* | plac_elon |  |  |  |  |  |  |  |  | NA |
| *placidochromis fuscus* | plac_fusc |  |  |  |  |  |  |  |  | NA |
| *placidochromis hennydaviesae* | plac_henn |  |  |  |  |  |  |  |  | NA |
| *placidochromis intermedius* | plac_inte |  |  |  |  |  |  |  |  | NA |
| *placidochromis johnstoni* | plac_john | GU936528 |  | GU946362 | EF585269 |  |  | JF262744 | HQ993468 | 2 |
| *placidochromis koningsi* | plac_koni |  |  |  |  |  |  |  |  | NA |
| *placidochromis lineatus* | plac_line |  |  |  |  |  |  |  |  | NA |
| *placidochromis longimanus* | plac_long |  |  |  |  |  |  |  |  | NA |
| *placidochromis longirostris* | plac_long |  |  |  |  |  |  |  |  | NA |
| *placidochromis longus* | plac_long |  |  |  |  |  |  |  |  | NA |
| *placidochromis lukomae* | plac_luko |  |  |  |  |  |  |  |  | NA |
| *placidochromis macroceps* | plac_macr |  |  |  |  |  |  |  |  | NA |
| *placidochromis macrognathus* | plac_macr |  |  |  |  |  |  |  |  | NA |
| *placidochromis mbunoides* | plac_mbun |  |  |  |  |  |  |  |  | NA |
| *placidochromis milomo* | plac_milo | GU936529 |  | GU946363 | EF585251 |  |  |  |  | 2 |
| *placidochromis minor* | plac_mino |  |  |  |  |  |  |  |  | NA |
| *placidochromis minutus* | plac_minu |  |  |  |  |  |  |  |  | NA |
| *placidochromis msakae* | plac_msak |  |  |  |  |  |  |  |  | NA |
| *placidochromis nigribarbis* | plac_nigr |  |  |  |  |  |  |  |  | NA |
| *placidochromis nkhatae* | plac_nkha |  |  |  |  |  |  |  |  | NA |
| *placidochromis nkhotakotae* | plac_nkho |  |  |  |  |  |  |  |  | NA |
| *placidochromis obscurus* | plac_obsc |  |  |  |  |  |  |  |  | NA |
| *placidochromis ordinarius* | plac_ordi |  |  |  |  |  |  |  |  | NA |
| *placidochromis orthognathus* | plac_orth |  |  |  |  |  |  |  |  | NA |
| *placidochromis pallidus* | plac_pall |  |  |  |  |  |  |  |  | NA |
| *placidochromis phenochilus* | plac_phen |  |  |  |  |  |  |  |  | NA |
| *placidochromis platyrhynchos* | plac_plat |  |  |  |  |  |  |  |  | NA |
| *placidochromis polli* | plac_poll |  |  |  |  |  |  |  |  | NA |
| *placidochromis rotundifrons* | plac_rotu |  |  |  |  |  |  |  |  | NA |
| *placidochromis subocularis* | plac_subo |  |  |  |  |  |  |  |  | NA |
| *placidochromis trewavasae* | plac_trew |  |  |  |  |  |  |  |  | NA |
| *placidochromis turneri* | plac_turn |  |  |  |  |  |  |  |  | NA |
| *placidochromis vulgaris* | plac_vulg |  |  |  |  |  |  |  |  | NA |
| *plecodus elaviae* | plec_elav |  |  |  | EF437504 |  |  |  |  | 0 |
| *plecodus multidentatus* | plec_mult |  |  |  | EF437505 |  |  |  |  | 0 |
| *plecodus paradoxus* | plec_para |  |  |  | EF437502 |  |  |  |  | 0 |
| *plecodus straeleni* | plec_stra | KC285408 |  |  | EF679258 | KF557123 |  |  |  | 2 |
| *protomelas annectens* | prot_anne | GU936533 |  | GU946367 | EU661718 |  |  |  |  | 2 |
| *protomelas dejunctus* | prot_deju |  |  |  |  |  |  |  |  | NA |
| *protomelas fenestratus* | prot_fene | GU936531 | AB090469 | GU946365 | GU946230 |  |  |  | HQ993469 | 3 |
| *protomelas insignis* | prot_insi |  |  |  |  |  |  |  |  | NA |
| *protomelas kirkii* | prot_kirk |  |  |  |  |  |  |  |  | NA |
| *protomelas labridens* | prot_labr |  |  |  |  |  |  |  |  | NA |
| *protomelas macrodon* | prot_macr |  |  |  |  |  |  |  |  | NA |
| *protomelas marginatus* | prot_marg |  |  |  |  |  |  |  |  | NA |
| *protomelas pleurotaenia* | prot_pleu |  |  |  |  |  |  |  |  | NA |
| *protomelas similis* | prot_simi | GU936538 |  | GU946372 | EU661714 |  |  |  |  | 2 |
| *protomelas spilonotus* | prot_spil | GU936532 |  | GU946366 | GU946231 |  |  |  |  | 2 |
| *protomelas spilopterus* | prot_spilo |  |  | GU946364 | EF585253 |  |  |  |  | 1 |
| *protomelas taeniolatus* | prot_taen | GU936534 |  | GU946368 | AF305302 |  |  |  |  | 2 |
| *protomelas triaenodon* | prot_tria |  |  |  |  |  |  |  |  | NA |
| *protomelas virgatus* | prot_virg |  |  |  |  |  |  |  |  | NA |
| *pseudocrenilabrus multicolor* | pseu_mult |  |  |  | AY930106 | DQ012215 |  |  |  | 1 |
| *pseudocrenilabrus multicolor victoriae* | pseu_multi |  |  |  | AY930070 |  |  |  |  | 0 |
| *pseudocrenilabrus nicholsi* | pseu_nich |  |  |  | AY602994 |  |  |  |  | 0 |
| *pseudocrenilabrus philander* | pseu_phil | KP130538 | KP130495 | KM263697 | AY602993 | KM263622 |  |  |  | 4 |
| *pseudosimochromis curvifrons* | pseu_curv | KC285396 |  |  | GQ995779 | KF557125 |  |  |  | 2 |
| *pseudotropheus ater* | pseu_ater |  |  |  |  |  |  |  |  | NA |
| *pseudotropheus benetos* | pseu_bene |  |  |  |  |  |  |  |  | NA |
| *pseudotropheus brevis* | pseu_brev |  |  |  |  |  |  |  |  | NA |
| *pseudotropheus crabro* | pseu_crab | GU936527 |  | GU946361 | EF585256 |  |  |  |  | 2 |
| *pseudotropheus cyaneorhabdos* | pseu_cyan |  |  |  |  |  |  |  |  | NA |
| *pseudotropheus cyaneus* | pseu_cyaneus |  |  |  |  |  |  |  |  | NA |
| *pseudotropheus demasoni* | pseu_dema |  |  |  |  |  |  |  |  | NA |
| *pseudotropheus elongatus* | pseu_elon |  |  |  | EF585272 |  |  |  |  | 0 |
| *pseudotropheus fainzilberi* | pseu_fain |  | HM049477 |  |  |  | HM049412 | HM049294 | HM049257 | NA |
| *pseudotropheus flavus* | pseu_flav |  |  | JX193869 |  |  |  |  |  | NA |
| *pseudotropheus fuscoides* | pseu_fusco |  |  |  |  |  |  |  |  | NA |
| *pseudotropheus fuscus* | pseu_fusc |  |  |  |  |  |  |  |  | NA |
| *pseudotropheus galanos* | pseu_gala |  |  |  |  |  |  |  |  | NA |
| *pseudotropheus interruptus* | pseu_inte |  |  |  |  |  |  |  |  | NA |
| *pseudotropheus joanjohnsonae* | pseu_joan |  |  |  |  |  |  |  |  | NA |
| *pseudotropheus johannii* | pseu_joha |  |  |  | GQ422574 |  |  |  | HQ993480 | 0 |
| *pseudotropheus longior* | pseu_long |  |  |  |  |  |  |  |  | NA |
| *pseudotropheus minutus* | pseu_minu |  |  |  |  |  |  |  |  | NA |
| *pseudotropheus perileucos* | pseu_peri |  |  |  |  |  |  |  |  | NA |
| *pseudotropheus perspicax* | pseu_pers |  |  |  |  |  |  |  |  | NA |
| *pseudotropheus purpuratus* | pseu_purp |  |  |  |  |  |  |  |  | NA |
| *pseudotropheus saulosi* | pseu_saul |  |  |  |  |  |  |  |  | NA |
| *pseudotropheus socolofi* | pseu_soco |  |  |  | JX157074 |  |  |  |  | 0 |
| *pseudotropheus tursiops* | pseu_turs |  |  |  |  |  |  |  |  | NA |
| *pseudotropheus williamsi* | pseu_will |  |  |  |  |  |  |  |  | NA |
| *pterochromis congicus* | pter_cong |  |  |  | GQ167807 |  |  |  |  | 0 |
| *pungu maclareni* | pung_macl |  |  |  | AJ845101 | KF557126 |  |  |  | 1 |
| *reganochromis calliurus* | rega_call |  |  |  | AY682544 | KF557127 |  |  |  | 1 |
| *rhamphochromis esox* | rham_esox | KP130561 |  | KP129844 | AF305252 | KP131336 |  |  | HQ993470 | 3 |
| *rhamphochromis ferox* | rham_fero |  |  |  |  |  |  |  |  | NA |
| *rhamphochromis longiceps* | rham_long |  |  |  | AF305246 |  |  |  |  | 0 |
| *rhamphochromis lucius* | rham_luci |  |  |  |  |  |  |  |  | NA |
| *rhamphochromis macrophthalmus* | rham_macr |  |  |  | AF305250 |  |  |  |  | 0 |
| *rhamphochromis woodi* | rham_wood |  |  |  |  |  |  |  |  | NA |
| *sargochromis carlottae* | sarg_carl |  |  |  | EF393683 |  |  |  |  | 0 |
| *sargochromis codringtonii* | sarg_codr |  |  |  | EF393717 |  |  |  |  | 0 |
| *sargochromis coulteri* | sarg_coul |  |  |  | EU753955 |  |  |  |  | 0 |
| *sargochromis giardi* | sarg_giar |  |  |  | EF393714 |  |  |  |  | 0 |
| *sargochromis mellandi* | sarg_mell |  |  |  | EF393702 |  |  |  |  | 0 |
| *sarotherodon caroli* | saro_caro |  |  |  | AJ845113 |  |  |  |  | 0 |
| *sarotherodon caudomarginatus* | saro_caud |  |  |  | GQ167819 |  |  |  |  | 0 |
| *sarotherodon galilaeus* | saro_gali |  |  |  | AJ845093 | KF557128 |  |  |  | 1 |
| *sarotherodon linnellii* | saro_linn |  |  |  | AJ845115 |  |  |  |  | 0 |
| *sarotherodon lohbergeri* | saro_lohb |  |  |  | AJ845109 |  |  |  |  | 0 |
| *sarotherodon melanotheron* | saro_mela |  |  |  | AF317245 |  |  |  |  | 0 |
| *sarotherodon mvogoi* | saro_mvog |  |  |  | GQ167811 |  |  |  |  | 0 |
| *sarotherodon nigripinnis* | saro_nigr |  |  |  | GQ167787 |  |  |  |  | 0 |
| *sarotherodon occidentalis* | saro_occi |  |  |  | AF317246 |  |  |  |  | 0 |
| *sarotherodon steinbachi* | saro_stei |  |  |  | AJ845111 |  |  |  |  | 0 |
| *schwetzochromis neodon* | schw_neod |  |  |  | EU753957 |  |  |  |  | 0 |
| *sciaenochromis ahli* | scia_ahli |  |  |  |  |  |  |  |  | NA |
| *sciaenochromis benthicola* | scia_bent |  |  |  | AF305298 |  |  |  |  | 0 |
| *sciaenochromis fryeri* | scia_frye |  |  |  | JX157077 |  |  |  |  | 0 |
| *sciaenochromis psammophilus* | scia_psam |  |  |  | AF305324 |  |  |  |  | 0 |
| *serranochromis altus* | serr_altu |  |  |  | EF393719 |  |  |  |  | 0 |
| *serranochromis angusticeps* | serr_angu |  |  |  | EF393710 |  |  |  |  | 0 |
| *serranochromis jallae* | serr_jall |  |  |  |  |  |  |  |  | NA |
| *serranochromis janus* | serr_janu |  |  |  |  |  |  |  |  | NA |
| *serranochromis longimanus* | serr_long |  |  |  |  |  |  |  |  | NA |
| *serranochromis macrocephalus* | serr_macr | KP130550 | KP130508 | KP129836 | EF393705 | KP131328 |  |  |  | 4 |
| *serranochromis meridianus* | serr_meri |  |  |  |  |  |  |  |  | NA |
| *serranochromis robustus* | serr_robu |  |  |  | EF393712 |  |  |  |  | 0 |
| *serranochromis spei* | serr_spei |  |  |  |  |  |  |  |  | NA |
| *serranochromis stappersi* | serr_stap |  |  |  | EF393699 |  |  |  |  | 0 |
| *serranochromis thumbergi* | serr_thum |  |  |  | EF393704 |  |  |  |  | 0 |
| *simochromis babaulti* | simo_baba |  |  |  | DQ093110 | DQ012224 |  |  |  | 1 |
| *simochromis diagramma* | simo_diag |  |  |  | AY930087 | DQ012228 |  |  |  | 1 |
| *simochromis margaretae* | simo_marg |  |  |  |  |  |  |  |  | NA |
| *simochromis marginatus* | simo_margi |  |  |  | AY930088 |  |  |  |  | 0 |
| *simochromis pleurospilus* | simo_pleu |  |  |  | GQ995782 |  |  |  |  | 0 |
| *spathodus erythrodon* | spat_eryt |  |  |  | AF317267 | DQ012218 |  |  |  | 1 |
| *spathodus marlieri* | spat_marl |  |  |  | HM623786 | KF557129 |  |  |  | 1 |
| *steatocranus bleheri* | stea_bleh |  |  |  | GQ167789 |  |  |  |  | 0 |
| *steatocranus casuarius* | stea_casu |  |  |  | AF317247 |  |  |  |  | 0 |
| *steatocranus gibbiceps* | stea_gibb |  |  |  | GQ167791 |  |  |  |  | 0 |
| *steatocranus glaber* | stea_glab |  |  |  | GQ167816 |  |  |  |  | 0 |
| *steatocranus irvinei* | stea_irvi |  |  |  | GQ167806 |  |  |  |  | 0 |
| *steatocranus tinanti* | stea_tina |  |  |  | AF317248 | KF557130 |  |  |  | 1 |
| *steatocranus ubanguiensis* | stea_uban |  |  |  | GQ167826 | KF557131 |  |  |  | 1 |
| *stigmatochromis macrorhynchos* | stig_macr |  |  |  |  |  |  |  |  | NA |
| *stigmatochromis melanchros* | stig_mela |  |  |  |  |  |  |  |  | NA |
| *stigmatochromis modestus* | stig_mode |  | AY780523 |  |  |  | AY775070 | AY775080 | AY775066 | NA |
| *stigmatochromis pholidophorus* | stig_phol |  |  |  |  |  |  |  |  | NA |
| *stigmatochromis pleurospilus* | stig_pleu |  |  |  |  |  |  |  |  | NA |
| *stigmatochromis woodi* | stig_wood |  |  |  | AF305299 |  |  |  |  | 0 |
| *stomatepia mariae* | stom_mari |  |  |  | AJ845097 |  |  |  |  | 0 |
| *stomatepia mongo* | stom_mong |  |  |  | AJ845095 |  |  |  |  | 0 |
| *stomatepia pindu* | stom_pind |  |  |  | AJ845099 | KF557132 |  |  |  | 1 |
| *taeniochromis holotaenia* | taen_holo |  |  |  |  |  |  |  |  | NA |
| *taeniolethrinops cyrtonotus* | taen_cyrt |  |  |  |  |  |  |  |  | NA |
| *taeniolethrinops furcicauda* | taen_furc |  |  |  | EF585263 |  |  |  |  | 0 |
| *taeniolethrinops laticeps* | taen_lati |  |  |  | AF305306 |  |  |  |  | 0 |
| *taeniolethrinops praeorbitalis* | taen_prae | GU936545 |  | GU946379 | AF305318 |  |  |  |  | 2 |
| *tangachromis dhanisi* | tang_dhan |  |  |  |  |  |  |  |  | NA |
| *tanganicodus irsacae* | tang_irsa |  |  |  | KJ176271 |  |  |  |  | 0 |
| *teleogramma depressa* | tele_depr |  |  |  | HM101360 |  |  |  |  | 0 |
| *telmatochromis bifrenatus* | telm_bifr |  |  |  | EF679239 |  |  |  |  | 0 |
| *telmatochromis brachygnathus* | telm_brac |  |  |  | KJ187229 | KJ399557 |  |  |  | 1 |
| *telmatochromis brichardi* | telm_bric |  |  |  | EF462236 |  |  |  |  | 0 |
| *telmatochromis dhonti* | telm_dhon |  |  |  | HM623804 |  |  |  |  | 0 |
| *telmatochromis temporalis* | telm_temp |  |  |  | HM623789 |  |  |  |  | 0 |
| *telmatochromis vittatus* | telm_vitt |  |  |  | AY740396 |  |  |  |  | 0 |
| *thoracochromis albolabris* | thor_albo |  |  |  | EU753929 |  |  |  |  | 0 |
| *thoracochromis bakongo* | thor_bako |  |  |  |  |  |  |  |  | NA |
| *thoracochromis brauschi* | thor_brau |  |  | KM263707 | AY930095 | KF557133 |  |  |  | 2 |
| *thoracochromis buysi* | thor_buys |  |  |  | EU753933 |  |  |  |  | 0 |
| *thoracochromis callichromus* | thor_call |  |  |  | JX157101 |  |  |  |  | 0 |
| *thoracochromis demeusii* | thor_deme |  |  |  | JX157095 |  |  |  |  | 0 |
| *thoracochromis fasciatus* | thor_fasc |  |  |  | JX157093 |  |  |  |  | 0 |
| *thoracochromis lucullae* | thor_lucu |  |  |  |  |  |  |  |  | NA |
| *thoracochromis moeruensis* | thor_moer |  |  |  |  |  |  |  |  | NA |
| *thoracochromis schwetzi* | thor_schw |  |  |  |  |  |  |  |  | NA |
| *thoracochromis stigmatogenys* | thor_stig |  |  |  | JX157107 |  |  |  |  | 0 |
| *thoracochromis wingatii* | thor_wing |  |  |  | JQ950400 |  |  |  |  | 0 |
| *thysochromis ansorgii* | thys_anso |  |  |  | AF317263 | KF359789 |  |  |  | 1 |
| *tilapia brevimanus* | tila_brev |  |  |  | AF317249 |  |  |  |  | 0 |
| *tilapia busumana* | tila_busu |  |  |  | AF317250 |  |  |  |  | 0 |
| *tilapia buttikoferi* | tila_butt |  |  |  | AF317251 | FJ616727 |  |  |  | 1 |
| *tilapia cabrae* | tila_cabr |  |  |  | AF317252 |  |  |  |  | 0 |
| *tilapia cessiana* | tila_cess |  |  |  | AF317253 |  |  |  |  | 0 |
| *tilapia coffea* | tila_coff |  |  |  | AF317254 |  |  |  |  | 0 |
| *tilapia dageti* | tila_dage |  |  |  | GQ167821 |  |  |  |  | 0 |
| *tilapia discolor* | tila_disc |  |  |  | AF317255 |  |  |  |  | 0 |
| *tilapia guinasana* | tila_guina |  |  |  | GQ167802 |  |  |  |  | 0 |
| *tilapia guineensis* | tila_guin |  |  |  | AF317256 |  |  |  |  | 0 |
| *tilapia joka* | tila_joka |  |  |  | GQ167803 |  |  |  |  | 0 |
| *tilapia louka* | tila_louk |  |  |  | AF317257 |  |  |  |  | 0 |
| *tilapia mariae* | tila_mari |  |  |  | AF317258 | KF557134 |  |  |  | 1 |
| *tilapia nyongana* | tila_nyon |  |  |  | GQ167827 |  |  |  |  | 0 |
| *tilapia rendalli* | tila_rend |  |  |  | AF317259 |  |  |  |  | 0 |
| *tilapia rheophila* | tila_rheo |  |  |  | GQ167825 |  |  |  |  | 0 |
| *tilapia ruweti* | tila_ruwe |  |  |  | GQ167799 | KF557135 |  |  |  | 1 |
| *tilapia sparrmanii* | tila_spar | KP130562 | KP130518 | KP129845 | AF317260 | KF557136 |  |  |  | 4 |
| *tilapia tholloni* | tila_thol |  |  |  | GQ167804 |  |  |  |  | 0 |
| *tilapia walteri* | tila_walt |  |  |  | AF317261 |  |  |  |  | 0 |
| *tilapia zillii* | tila_zill |  |  |  | AF317262 |  |  |  |  | 0 |
| *tramitichromis brevis* | tram_brev |  |  |  | AF305320 |  |  |  |  | 0 |
| *tramitichromis intermedius* | tram_inte |  | DQ088629 |  |  |  | GQ422501 | DQ088638 | DQ088641 | NA |
| *tramitichromis lituris* | tram_litu |  |  |  |  |  |  |  |  | NA |
| *tramitichromis trilineatus* | tram_tril |  |  |  |  |  |  |  |  | NA |
| *tramitichromis variabilis* | tram_vari |  |  |  | AF305319 |  |  |  |  | 0 |
| *trematocara unimaculatum* | trem_unim |  |  |  | AF317268 | DQ012227 |  |  |  | 1 |
| *trematocranus labifer* | trem_labi |  |  |  |  |  |  |  |  | NA |
| *trematocranus microstoma* | trem_micr |  |  |  |  |  |  |  |  | NA |
| *trematocranus placodon* | trem_plac |  |  |  | EF585261 |  |  |  | HQ993471 | 0 |
| *triglachromis otostigma* | trig_otos |  |  |  | AY682546 |  |  |  |  | 0 |
| *tristramella simonis* | tris_simo |  |  |  | AF317276 |  |  |  |  | 0 |
| *tropheops broad mouth* | trop_broa |  |  |  | EF599101 |  |  |  |  | 0 |
| *tropheops red cheek* | trop_red |  |  |  | GQ422568 |  |  |  |  | 0 |
| *tropheops tropheops* | trop_trop | GU936541 |  | DQ239800 | AY740384 |  |  |  |  | 2 |
| *tropheus annectens* | trop_anne |  |  |  |  |  |  |  |  | NA |
| *tropheus brichardi* | trop_bric |  |  |  | AY930086 |  |  |  |  | 0 |
| *tropheus duboisi* | trop_dubo |  | AY780516 |  | AY930085 | KF557137 | AY775089 | AY775073 | AY775082 | 2 |
| *tropheus kasabae* | trop_kasa |  |  |  |  |  |  |  |  | NA |
| *tropheus moorii* | trop_moor | KC285391 | KP130501 | JX135302 | AY930093 | KP131323 |  |  |  | 4 |
| *tropheus polli* | trop_poll |  |  |  | AY930084 |  |  |  |  | 0 |
| *tylochromis leonensis* | tylo_leon |  |  |  | AF317274 |  |  |  |  | 0 |
| *tylochromis polylepis* | tylo_poly |  | KP130504 | KP129833 | AB018973 | KP131325 |  |  |  | 3 |
| *tyrannochromis macrostoma* | tyra_macr | GU936543 | AB090476 | GU946377 | EF585257 |  |  |  | HQ993476 | 3 |
| *tyrannochromis maculiceps* | tyra_macu | GU936544 | AY780520 | GU946378 | GQ422571 |  | AY775093 | AY775078 | AY775086 | 3 |
| *tyrannochromis nigriventer* | tyra_nigr |  |  |  | AF305307 |  |  |  |  | 0 |
| *tyrannochromis polyodon* | tyra_poly |  |  |  |  |  |  |  |  | NA |
| *variabilichromis moorii* | vari_moor | KC285395 | KP130489 | KP129822 | GQ167833 | FJ706503 |  |  |  | 4 |
| *xenochromis hecqui* | xeno_hecq |  |  |  | EF437514 |  |  |  |  | 0 |
| *xenotilapia albini* | xeno_albi |  |  |  |  |  |  |  |  | NA |
| *xenotilapia bathyphila* | xeno_bath |  | HM135141 |  | AY337789 |  | HM135119 |  |  | 1 |
| *xenotilapia boulengeri* | xeno_boul |  | HM135137 |  | HM135111 |  | HM135123 |  |  | 1 |
| *xenotilapia burtoni* | xeno_burt |  |  |  |  |  |  |  |  | NA |
| *xenotilapia caudafasciata* | xeno_caud |  |  |  | AY337777 |  |  |  |  | 0 |
| *xenotilapia flavipinnis* | xeno_flav |  | HM135138 |  | AY337794 |  | HM135126 |  |  | 1 |
| *xenotilapia leptura* | xeno_lept |  | HM135129 |  | EF679238 |  | HM135118 |  |  | 1 |
| *xenotilapia longispinis* | xeno_long |  |  |  | AY337779 |  |  |  |  | 0 |
| *xenotilapia melanogenys* | xeno_mela |  | HM135131 |  | AY337770 | DQ012238 | HM135115 |  |  | 2 |
| *xenotilapia nasus* | xeno_nasu |  |  |  |  |  |  |  |  | NA |
| *xenotilapia nigrolabiata* | xeno_nigr |  |  |  |  |  |  |  |  | NA |
| *xenotilapia ochrogenys* | xeno_ochr |  | HM135142 |  | AY337767 | DQ012221 | HM135128 |  |  | 2 |
| *xenotilapia ornatipinnis* | xeno_orna |  |  |  |  |  |  |  |  | NA |
| *xenotilapia papilio* | xeno_papi |  |  |  |  |  |  |  |  | NA |
| *xenotilapia rotundiventralis* | xeno_rotu |  |  |  | AY337793 |  |  |  |  | 0 |
| *xenotilapia sima* | xeno_sima |  |  |  | AY337785 |  |  |  |  | 0 |
| *xenotilapia spiloptera* | xeno_spil | KP130524 | KP130481 | KP129814 | AY337788 | KP131306 | HM135117 |  |  | 4 |
| *xenotilapia tenuidentata* | xeno_tenu |  |  |  | AY337784 |  |  |  |  | 0 |
